# Design, Synthesis, Dynamic Docking, Biochemical Characterization, and *in Vivo* Pharmacokinetics Studies of Novel Topoisomerase II Poisons with Promising Antiproliferative Activity

**DOI:** 10.1021/acs.jmedchem.9b01760

**Published:** 2020-03-20

**Authors:** Jose M. Arencibia, Nicoletta Brindani, Sebastian Franco-Ulloa, Michela Nigro, Jissy Akkarapattiakal Kuriappan, Giuliana Ottonello, Sine Mandrup Bertozzi, Maria Summa, Stefania Girotto, Rosalia Bertorelli, Andrea Armirotti, Marco De Vivo

**Affiliations:** † Molecular Modeling and Drug Discovery Lab, Istituto Italiano di Tecnologia, Via Morego 30, 16163 Genova, Italy; ‡ Analytical Chemistry and in Vivo Pharmacology, Istituto Italiano di Tecnologia, Via Morego 30, 16163 Genova, Italy

## Abstract

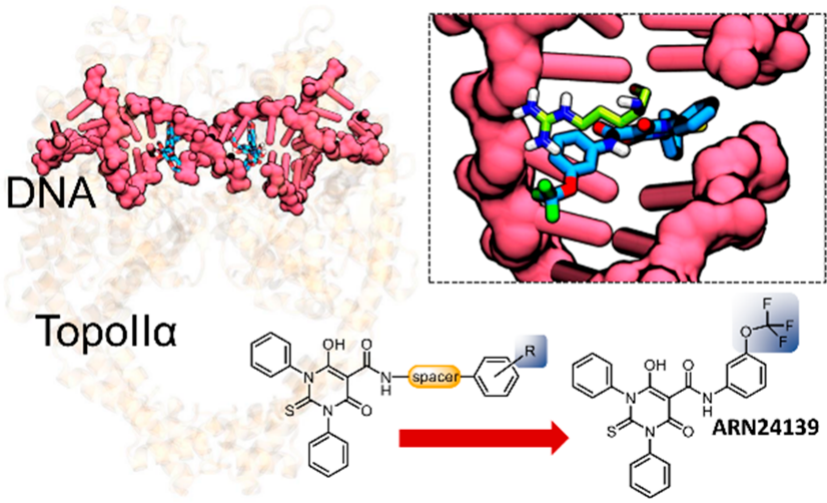

We previously reported a first set of hybrid topoisomerase II (topoII) poisons whose chemical core merges key pharmacophoric elements of etoposide and merbarone, which are two well-known topoII blockers. Here, we report on the expansion of this hybrid molecular scaffold and present 16 more hybrid derivatives that have been designed, synthesized, and characterized for their ability to block topoII and for their overall drug-like profile. Some of these compounds act as topoII poison and exhibit good solubility, metabolic (microsomal) stability, and promising cytotoxicity in three cancer cell lines (DU145, HeLa, A549). Compound **3f** (ARN24139) is the most promising drug-like candidate, with a good pharmacokinetics profile *in vivo*. Our results indicate that this hybrid new chemical class of topoII poisons deserves further exploration and that **3f** is a favorable lead candidate as a topoII poison, meriting future studies to test its efficacy in *in vivo* tumor models.

## Introduction

Human topoisomerase II (topoII) enzymes are a validated target to treat cancer because of their role in modifying the topology of entangled DNA strands during vital cellular processes like replication and transcription.^1−4^ Several topoII anticancer inhibitors are clinically available. One example is etoposide, which is used to treat a variety of cancers, including leukemia and ovarian cancer.^5−9^ However, drug resistance and the possibility of severe side effects of topoII-targeting drugs mean that researchers continue to seek novel safer topoII inhibitors.^6,10−17^

**Figure 1:**
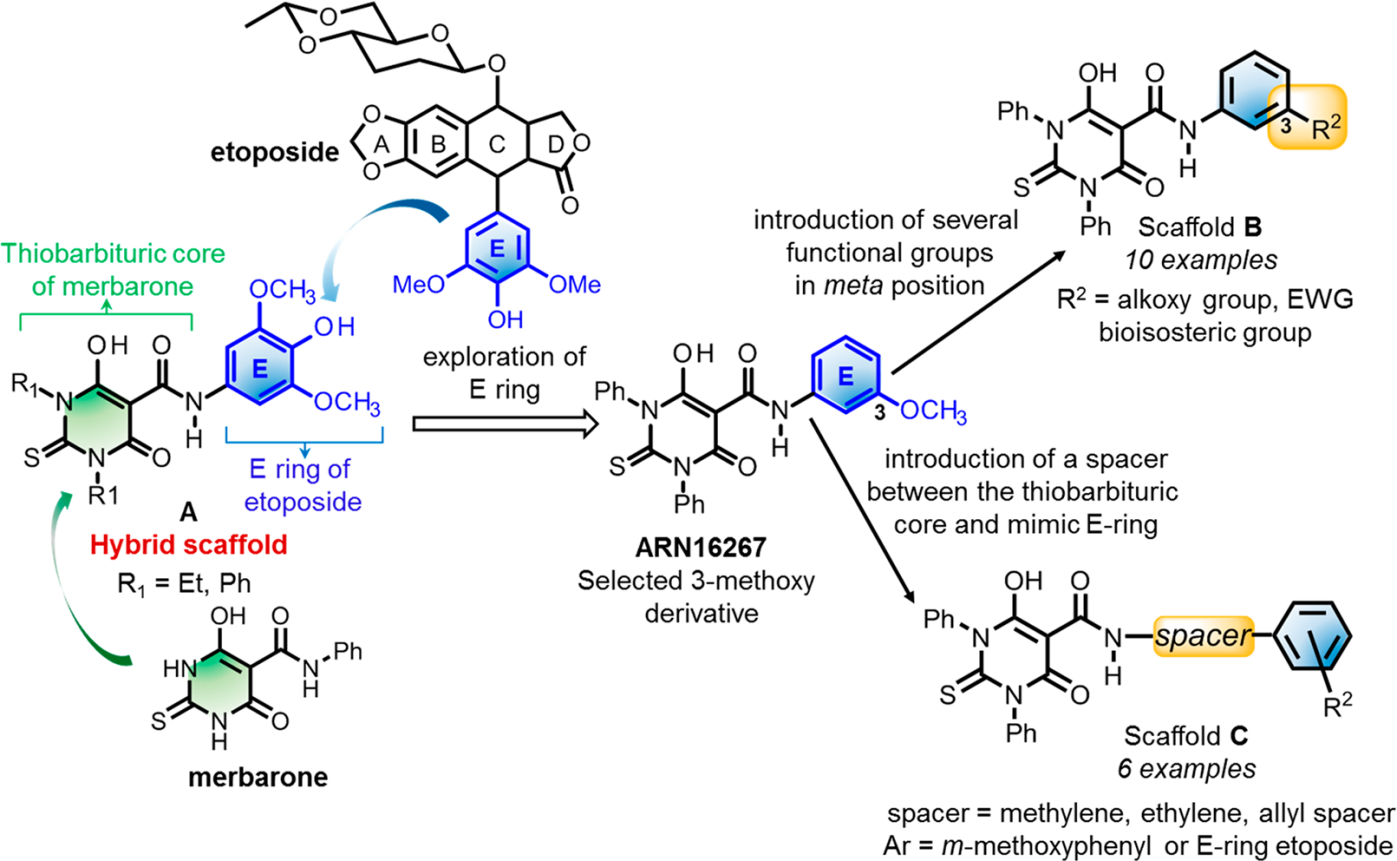
Hybrid topoII poison with the scaffold **A** (left) was explored to discover ARN16267 as a potent topoII blocker.^39^ Here, the hybrid scaffold **A** was expanded to generate structures of type **B** (upper right), with several functional groups introduced in the *meta* position. Structures of type **C** (lower right) were generated by introducing a spacer between the thiobarbituric core and the mimic E-ring.

Small molecules targeting topoII are classified as either topoII poisons or topoII catalytic inhibitors.^18−20^ These two classes of topoII blockers differ in their mode of action. TopoII poisons act by trapping the covalent topoII/DNA cleavage complex, which is formed during the catalytic cycle required for DNA topology modification. A covalent and stable topoII/DNA cleavage complex eventually leads to the accumulation of double-strand breaks, causing cell death.^1,12,13,18,21−23^ The chemotherapy drug etoposide (Figure 1) acts via this mechanism, although its pharmacological action can lead to severe side effects.^24−26^ Additional examples of anticancer drugs^27−30^ that act as a topoII poison are doxorubicin, mitoxantrone, salvicine, and teniposide. These drugs are frontline therapies for a wide range of solid and hematological malignancies.^31−33^

TopoII catalytic inhibitors act differently than poisons and do not generate an accumulation of topoII/DNA cleavage complex. Instead, topoII catalytic inhibitors act, for example, by inhibiting DNA binding and/or by blocking the ATP-binding site in topoII, thus preventing ATP hydrolysis, which is needed for topoII function.^19^ One notable example is merbarone, which was one of the first and most promising topoII inhibitors (Figure 1).^34−37^ Merbarone is a thiobarbituric derivative (6-hydroxy-4-oxo-*N*- phenyl-2-thioxo-1*H*-pyrimidine-5-carboxamide) that blocks topoII catalysis and inhibits proliferation of several cancer cell lines.^35^ Notably, merbarone underwent clinical trials as a chemotherapy drug.^34,36^ These trials failed because merbarone displayed nephrotoxicity issues and did not generate the expected efficacy.^38^

Recently, we used a pharmacophore hybridization strategy to realize a first set of new topoII poisons.^39^ They were rationally designed by combining key pharmacophoric elements of etoposide and merbarone to generate a new etoposide-merbarone hybrid active scaffold.^39,40^ In particular, we designed, synthesized, and characterized a first set of compounds that feature the thiobarbituric core of merbarone linked via an amide bond to the E-ring of etoposide (type A structure, Figure 1). This design generated new *N*,*N*′-diphenyl derivatives that potently block human topoII.^39^ In addition, our SAR studies clarified the effect of ethyl and phenyl substitutions at each nitrogen of the thiourea moiety, as well as the influence of the number and/or position of hydroxyl and methoxy substituents on the mimic E-ring.^39^ Importantly, we identified compound ARN16267 (IC_50_ = 30 ± 6 μM, structure in Figure 1, which was originally named compound **3** in ref (39)), which is a more potent topoII blocker than the template compounds, i.e., etoposide (IC_50_ = 120 ± 10 μM) and merbarone (IC_50_ = 120 ± 12 μM).^39^ Intriguingly, we found that ARN16267 was the most efficient of this new chemical class in generating accumulation of topoII/DNA cleavage complex. This suggests that ARN16267 may act as a topoII poison, although this mechanism was less marked than that of etoposide.^39^

**Scheme 1:**
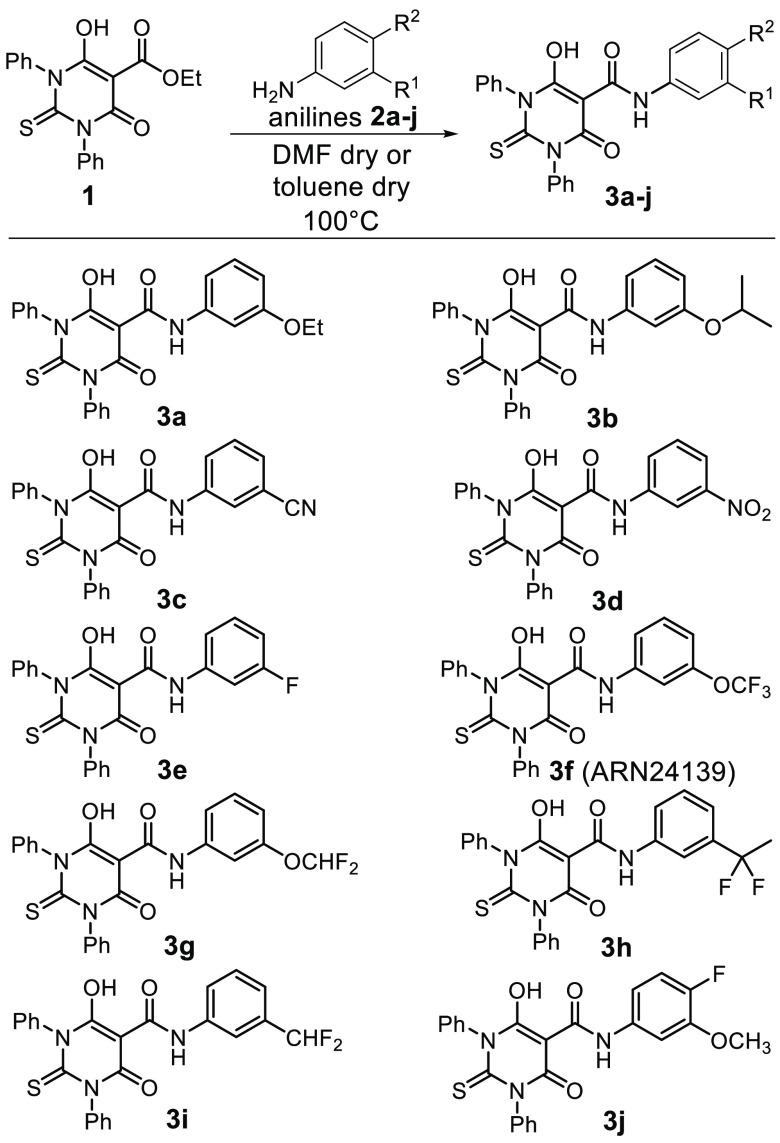
Synthesis of Compounds **3a**–**j** Our lead compound **3f** (ARN24139) is in the right column.

These results prompted us to investigate the SAR of these new hybrid topoII blockers. Here we present an additional 16 derivatives that expand the initial panel of merbarone–etoposide hybrid molecules.^39^ As described in Figure 1, we used ARN16267 as our best starting point for further derivatization of its core scaffold, generating scaffolds of types B and C (Figure 1). We thus identified a new hybrid derivative (**3f**, ARN24139; see Scheme 1) with improved human topoII inhibitory activity (7.3 ± 1.5 μM). In addition, our results confirm that this new class of hybrid compounds acts as topoII poisons, generating accumulation of topoII/DNA cleavage complex. Our dynamic docking simulations support binding of **3f** at the cleavage complex. Additionally, **3f** showed high kinetic solubility and metabolic stability, as well as a promising antiproliferative activity in the low μM range against DU145, HeLa, and A549 cancer cell lines. Finally, we found **3f** to have a good pharmacokinetic profile *in vivo*. Thus, **3f** can be added to the pipeline of compounds that are active against topoII with promising anticancer activity.^41−43^

## Results and Discussion

### Exploring the Structure of the New Hybrid Scaffold

For our new set of hybrid analogues and based on our previous work and results,^39^ we initially expanded our SAR studies by exploring the effect of diverse functional groups in the *meta* position on the mimic E-ring (**3a**–**j**, Scheme 1). First, we synthesized two new compounds with an ethoxy (**3a**) or isopropoxy (**3b**) group in the *meta* position on the mimic E-ring. We previously found that replacing the methoxy in ARN16267 with a hydroxyl group significantly decreased topoII inhibitory activity.^39^ We thus substituted the original methoxy in ARN16267 with a cyano (**3c**), nitro (**3d**), or fluoro (**3e**) to modulate the electron density of the mimic E-ring. Additionally, we investigated the bioisosteric replacement of the methoxy group of ARN16267 and generated three additional new hybrid compounds, each bearing a trifluoromethoxy (**3f**), difluoromethoxy (**3g**), or difluoroethoxy (**3h**) group in the *meta* position on the mimic E-ring. Similarly, we synthesized a derivative with the difluoromethyl group in the *meta* position on the mimic E-aromatic ring (**3i**). Finally, given that a fluorine proximal to a methoxy can influence the overall electronic behavior of the aromatic ring,^44^ we inserted a fluoro in the *para* position of ARN16267 to obtain **3j** (Scheme 1).

**Scheme 2:**
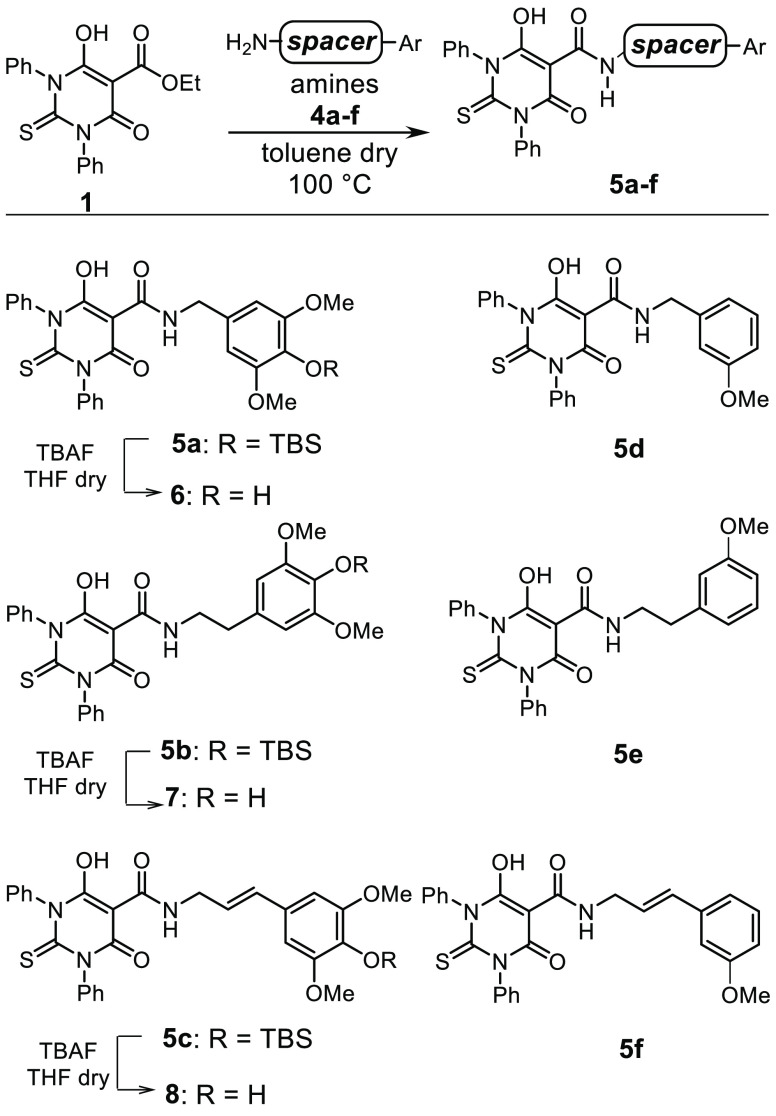
Synthesis of Compounds **6**–**8** and **5d**–**f**

In the crystal structure of the ternary topoII/DNA/etoposide complex, the drug molecule is stabilized by interactions with Asp463 and Arg487.^25^ To favor the formation of these interactions for our hybrid compounds, we introduced a flexible spacer between the thiobarbituric core and the mimic E-ring (compounds **6**–**8** and **5d**–**f**, Scheme 2).^25,45−47^ To test this hypothesis, we generated an additional set of six compounds with a link-mediated increased flexibility (**6**–**8**, **5d**–**f**).

### Chemistry

**Scheme 3:**
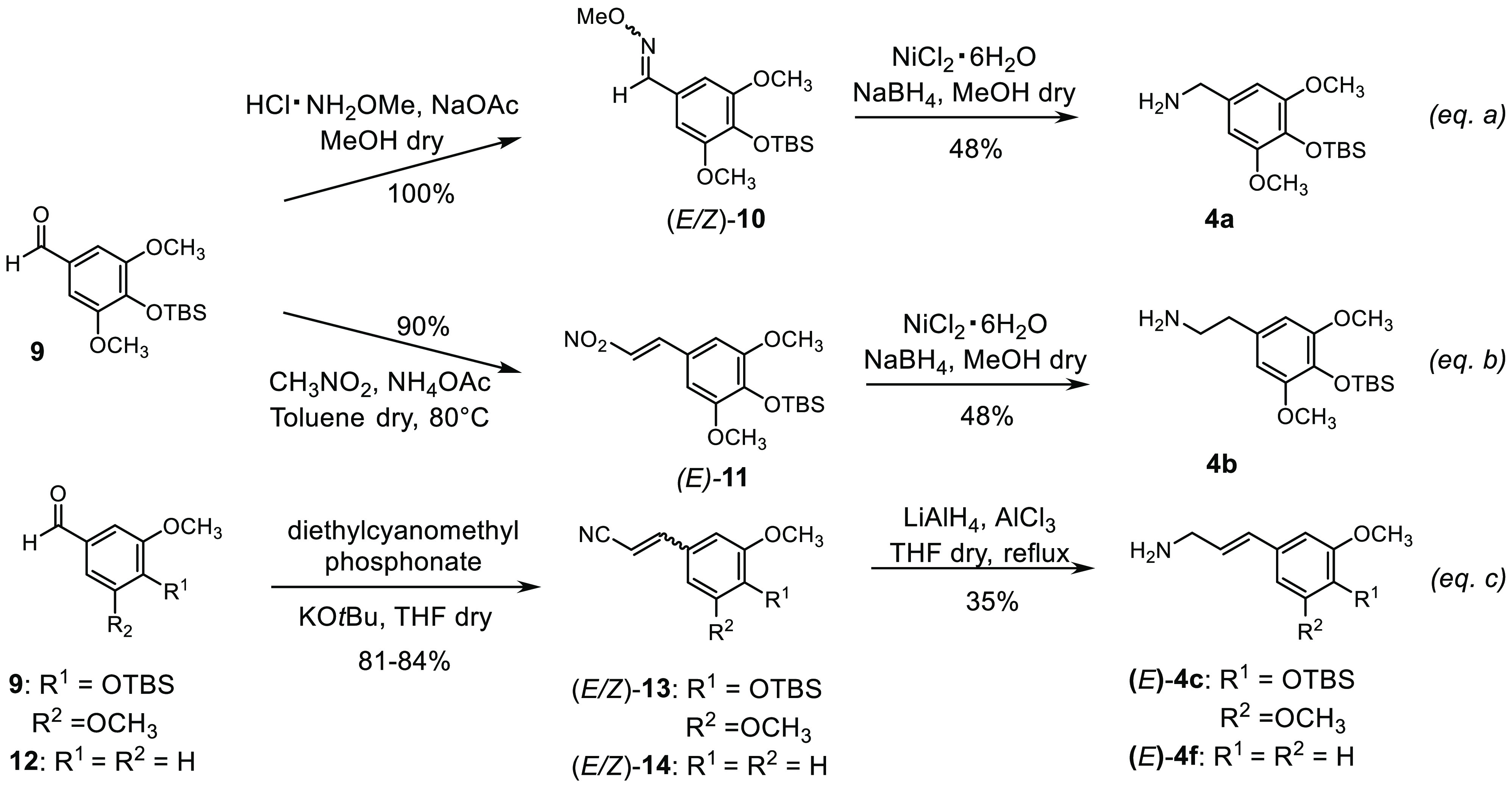
Synthesis of Amines **4a**–**c** and **4f**

The 16 new derivatives were synthesized through regular amidation of ester **1**(39) with amines **2a**–**j** and **4a**–**f** using either DMF or toluene dry as a solvent, with yields that ranged from 34% to 72% (Schemes 1 and 2). As previously described,^48^ in the presence of amine **2j** substituted with a fluorine in *para* and a methoxy group in *meta* position, the formylation side reaction performed by DMF was preferred over the alternative and desired reaction with ester **1**.^48,49^ To circumvent this problem, the reaction was conducted in toluene dry at 100 °C, obtaining **3j** with good yield (72%). The same strategy was used to synthesize **6**–**8** and **5d**–**f** (Scheme 2), where different hydrocarbon chains were introduced between the amide and the aromatic ring. In particular, amines **4a**–**c** and **4f** (used to prepare **6**–**8** and **5f**) were synthesized in two steps starting from silyl protected syringaldehyde **9** and *m*-anisaldehyde **12**, respectively (Scheme 3). Compound **4a** is a benzylamine featuring an aromatic ring with the same functionalization of the E ring of etoposide. This was obtained through the quantitative conversion of aldehyde **9** in the related *O*-methyl oxime **10**, which was reduced into the desired amine **4a** with NiCl_2_ and NaBH_4_ with 48% yield (Scheme 3, eq a).

The Henry reaction between protected syringaldehyde **9**(50) and nitromethane in the presence of ammonium acetate gave (*E*)-nitrostyrene **11** with an excellent 90% yield. Compound **11** was then completely reduced with NiCl_2_ and NaBH_4_ into phenylethanamine **4b** with 48% yield (Scheme 3, eq b). The Horner–Wadsworth–Emmons (HWE) reaction between aldehyde **9**(50) and diethyl cyanomethylphosphonate in the presence of potassium *tert*-butoxide in THF gave a 1:0.12 mixture of (*E*)- and (*Z*)-acrylonitrile **13** in 84% yield. The chemoselective nitrile reduction with LiAlH_4_ and aluminum trichloride afforded (*E*)-allylamine **4c** after chromatography purification, with an acceptable 35% yield (Scheme 3, eq c). The same sequential transformations of olefination and reduction allowed the introduction of an allylic spacer for amine **4f**, starting from *m*-anisaldehyde **12**, with a 22% overall yield (Scheme 3, eq c). In this case, too, the (*E*)-isomer was obtained pure after chromatography purification. Thus, the key amidation reaction between silylated amines **4a**–**c** with ester **1** in toluene dry at 100 °C generated amides **5a**–**c**, which were deprotected using TBAF. This yielded our final targets **6**–**8** with a free hydroxylic group in *para* position, with 22–38% yield after two steps (Scheme 2). Similarly, *m*-methoxyamide counterparts **5d**–**f** were obtained with 38–62% yield using related amines **4d**–**f** (Scheme 2).

### TopoII Inhibitory Activity of the Novel Hybrid Compounds

We measured the inhibitor activity of our new compounds against human topoIIα, using a topoII decatenation assay (Table 1). Notably, in our assay, etoposide returned an IC_50_ of 47.5 ± 2.2 μM, which agrees with the activity reported by the manufacturer of the decatenation assay kit.^51^ Additionally, merbarone showed an IC_50_ of 26.0 ± 4.7 μM, which is in line with that reported previously (IC_50_ = 40 μM) using a plasmid relaxation assay.^35^

**Table 1:** Inhibitory and Antiproliferative Activities of 16 New Derivatives **3a**–**j**, **6**–**9**, and **5d**–**f**^a^

compd	IC_50_ (μM)	DU145 (μM)	HeLa (μM)	A549 (μM)
etoposide	47.5 ± 2.2	1.0 ± 0.8	2.4 ± 0.9	1.3 ± 0.1
merbarone	26.0 ± 4.7	18.9 ± 2.0	62.3 ± 6.4	40.0 ± 2.7
ARN16267	16.1 ± 2.4	7.6 ± 0.8	5.5 ± 1.3	4.7 ± 0.3
**3a**	12.4 ± 3.7	5.7 ± 1.4	5.2 ± 0.9	3.6 ± 0.4
**3b**	9.7 ± 2.6	5.5 ± 0.1	4.2 ± 0.3	3.0 ± 0.3
**3c**	14.4 ± 3.8	14.4 ± 4.1	12.4 ± 1.3	16.9 ± 0.3
**3d**	10.2 ± 4.6	6.9 ± 0.3	11.9 ± 2.6	18.5 ± 3.1
**3e**	15.1 ± 4.2	5.6 ± 0.6	5.6 ± 0.5	4.1 ± 0.2
**3f**	7.3 ± 1.5	4.7 ± 0.1	3.8 ± 0.3	3.1 ± 0.1
**3g**	10.2 ± 1.9	6.5 ± 0.9	6.4 ± 0.5	4.8 ± 0.4
**3h**	9.2 ± 0.2	2.7 ± 2.5	2.5 ± 0.4	3.0 ± 1.1
**3i**	11.4 ± 2.4	7.8 ± 0.2	4.9 ± 1.4	4.4 ± 0.2
**3j**	22.5 ± 5.8	7.8 ± 0.6	5.3 ± 1.0	4.6 ± 0.1
**5d**	22.5 ± 7.2	7.7 ± 0.1	6.1 ± 1.1	4.8 ± 0.4
**5e**	15.8 ± 3.4	3.3 ± 2.5	3.4 ± 0.1	3.2 ± 1.2
**5f**	8.3 ± 2.3	5.0 ± 2.9	5.5 ± 1.1	4.4 ± 0.6
**6**	107.8 ± 10.1	8.8 ± 0.3	19.1 ± 4.8	14.7 ± 0.3
**7**	74.4 ± 13.6	13.9 ± 7.6	13.3 ± 1.7	14.8 ± 1.4
**8**	28.0 ± 4.4	9.8 ± 2.1	9.3 ± 0.2	9.5 ± 1.5

a Antiproliferative activity in cancer cells of etoposide and merbarone was measured in ref (39).

Interestingly, greater bulkiness of the alkyl chain on the oxygen in the *meta* position, as in **3a** and **3b**, improved the potency of these compounds, as compared to ARN16267, which has an IC_50_ of 16.1 ± 2.4 μM, as measured in the decatenation assay used in this study. In fact, **3b**, with the bulkier substituent, had an IC_50_ of 9.7 ± 2.6 μM, while the ethoxy substitution in **3a** returned an IC_50_ of 12.4 ± 3.7 μM (Table 1). We then found that the electron-withdrawing nitro group, in the *meta* position on the mimic E-ring in **3d**, returned an IC_50_ of 10.2 ± 4.6 μM. Other electron-withdrawing groups such as the cyano (**3c**) and the fluoro (**3e**) returned a comparable activity to that of ARN16267 (Table 1). This is in line with our previous demonstration of the unfavorability of an electron-donating group, such as a hydroxyl substitution, at this position.^39^ Similarly, introducing a fluorine proximal to the methoxy group (**3j**) was detrimental for activity, with an IC_50_ of 22.5 ± 5.8 μM.

We then investigated the bioisosteric replacement of the methoxy group in *meta* position on the mimic E-ring. Interestingly, all the bioisosteric analogues **3f**–**h** displayed a better activity than ARN16267: introducing a trifluoromethoxy group in **3f** returned a 2-fold increased activity with an IC_50_ of 7.3 ± 1.5 μM, while difluoroethyl in **3h** returned an IC_50_ of 9.2 ± 0.2 μM, and the difluoromethoxy group in **3g** returned an IC_50_ of 11.4 ± 2.4 μM. Notably, the difluoromethyl analogue **3i** also had this improved activity, which confirms that the additional interactions provided by fluorinated groups (also more lipophilic) can compensate the loss of the oxy-moiety in *meta* position of the aromatic (mimic) E-ring.

After evaluating the inhibitory activity of this first subset of derivatives, we assessed the activity of the hybrid molecules with a flexible spacer connecting the thiobarbituric core and the mimic E-ring (**5d**–**f** and **6**–**8**, Scheme 2). We started evaluating the activity of the compound **6**, where we inserted a methylene substitution that contains the exact E-ring of etoposide. This first modification reduced the potency (IC_50_ = 107.8 ± 10.1 μM), with a 7-fold drop in activity compared to ARN16267 (Table 1). Conversely, inserting the same substitution in **5d** only decreased 1.4-fold the inhibitory activity (IC_50_ = 22.5 ± 7.2 μM) compared to the parent compound ARN16267 (Table 1). This result confirms that a methoxy group, alone, in the mimic E-ring increases the potency of this hybrid scaffold, as also observed previously.^39^ Increasing the spacer length was also beneficial, improving the IC_50_ from over 100 μM for **6** to 74.4 ± 13.6 and 28.0 ± 4.4 μM for **7** and **8**, respectively. This positive trend in potency could be due to a more balanced structure where the flexibility introduced by having the hydrocarbon chain (i.e., the spacer) is compensated by the introduced rigidity of the C=C double bond embedded in the allylic system in **8**. Within this second class of analogues, compounds are more potent when the 3-methoxy was retained as the only substituent, with IC_50_ values of 22.5 ± 7.2 μM for **5d**, 15.8 ± 3.4 μM for **5e**, and 8.3 ± 2.3 μM for **5f**. Notably, **5f** showed a 2-fold increase in IC_50_ compared to its template ARN16267 (which has no spacer). These data thus suggest that a flexible substituent connecting the thiobarbituric core and the mimic E-ring in our hybrid scaffold may facilitate the orientation of our molecules inside the active site of topoII, increasing their inhibitory potency (see docking results, below).

### Antiproliferative Activity against Cultured Human Cancer Cells, Metabolic Stability, Chemical Solubility, and TopoII Poisoning

The antiproliferative activity of all compounds was evaluated in (i) DU145, an androgen-independent prostate cancer cell line; (ii) HeLa, a cervical cancer cell line; and (iii) A549, a lung adenocarcinoma cell line (Table 1). Notably, all new compounds showed good antiproliferative activity with IC_50_ values in the low μM range. Among the most active compounds in inhibiting topoII activity, **3f**, **3h**, and **5e** showed cytotoxicity with IC_50_ values lower than 5 μM (Table 1). Undoubtedly, this preliminary cytotoxicity data will need further characterization, also in relation to the *in vitro* activity of these compounds.

After this initial evaluation of the new set of hybrid compounds for their inhibitory activity against topoII *in vitro* and for their biological cytotoxicity, we selected **3b**, **3f**–**i**, and **5e** for further evaluation. We assessed their metabolic stability using mouse serum and mouse liver microsomes, and we assessed their kinetic solubility in neutral buffer. These compounds had excellent plasma and microsomal stability with half-time values greater than 120 and 60 min, respectively. Additionally, **3f**, **3g**, and **3i** displayed excellent solubility in aqueous buffer (pH 7.4), with values greater than 200 μM (Table 2).

**Table 2:** Kinetic Solubility of **3b**, **3f**–**h**, **5e**

compd	kinetic solubility (μM)
ARN16267	236
**3b**	34
**3f**	224
**3g**	208
**3h**	6
**3i**	238
**5e**	122

**Figure 2:**
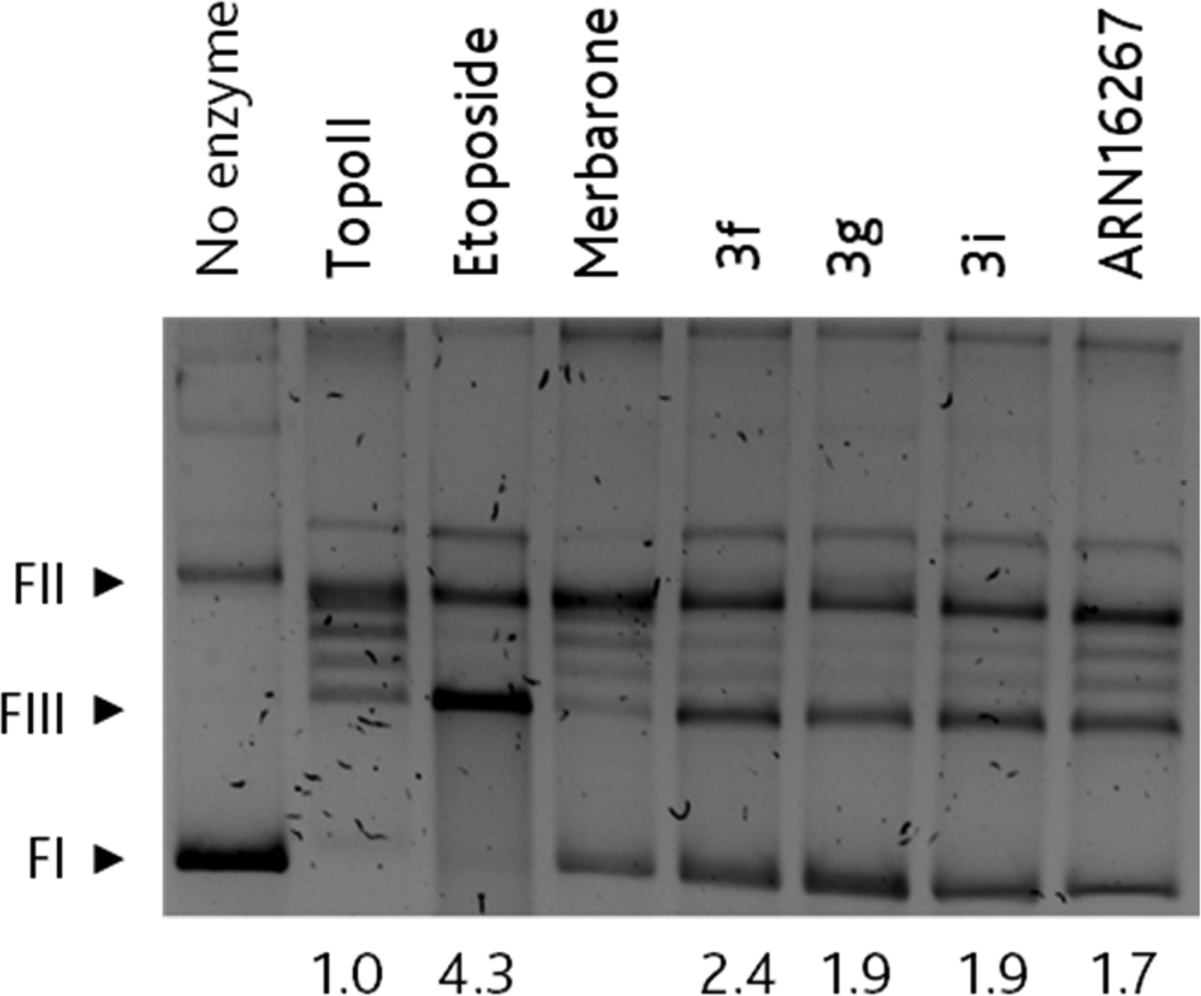
Poison activity of the hybrid compounds. Agarose gel electrophoresis of plasmid DNA incubated in the absence (no enzyme) or presence of 1 U of topoII containing either 1% DMSO as control vehicle (no enzyme and topoII lanes) or 200 μM compound. Labels are shown above each lane. Numbers at the bottom correspond to the normalized intensity of the linear form (FIII). Plasmid forms are indicated by the arrow-points on the right: supercoiled (FI), nicked (FII), and linear (FIII).

In view of these results, **3f**, **3g**, and **3i** were tested in a cleavage complex formation assay to further ascertain their mode of action as topoII poisons. As shown in Figure 2, all these hybrid molecules were confirmed to be poisons and thus able to generate an accumulation of topoII/DNA cleavage complex. In particular, **3f** had the greatest poison efficacy, being about 1.5-fold better than the template ARN16267, at 200 μM concentration. Given **3f**’s promising *in vitro* activity as a topoII poison and its overall drug-like profile, we examined its binding mode to topoIIα and its *in vivo* pharmacokinetic profile in mice.

### Docking and Molecular Simulations of **3f** Bound to the Target

**Figure 3:**
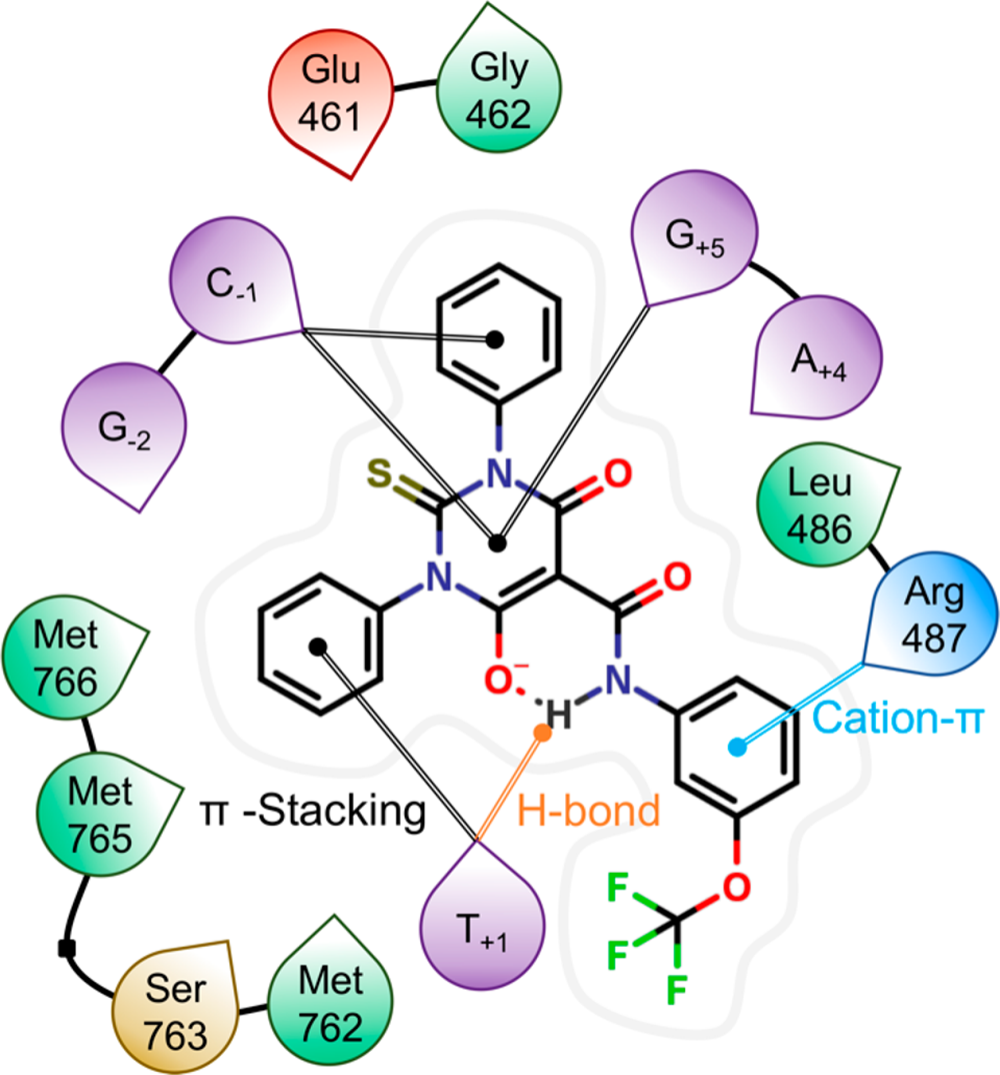
Interaction diagram of the lead compound **3f** bound to the DNA/topoII complex as derived from docking calculations. The computational study flags a cation−π interaction with Arg487, π-stacking with various DNA bases, and an H-bond with T_+1_, as the main drivers for ligand binding.

We used docking and atomistic force-field-based molecular dynamics (MD) simulations to model **3f** bound to the topoII/DNA cleavage site.^52−55^ First, the crystal structure of the topoIIα isoform (PDB code 5GWK) was used for the docking studies.^25,56^ As seen in Figure S2, when the compound was first docked into the cleavage site, the mimic E-ring slightly shifted relative to the position of the E-ring of etoposide in the crystal.^25^ Our calculations revealed several key contacts between the ligand and vicinal residues that confer the system a stable, inhibited conformation, thus endorsing the compound’s action as a topoII poison. The protein aids the ligand’s anchoring within the pocket by a cation−π link formed between Arg487 and the E-ring. The neighboring DNA bases also contribute to the stabilization of the complex. Specifically, the G_+5_ and C_–1_ bases display π-stacking interactions with the heterocycle inherited from merbarone (Figure 3). Similarly, the T_+1_ and C_–1_ bases align to the phenyl substituents at the thiobarbituric core in a perpendicular fashion resembling a T-shaped π-interaction. Finally, an H-bond between T_+1_ and the ligand’s N–H moiety was also identified.

**Figure 4:**
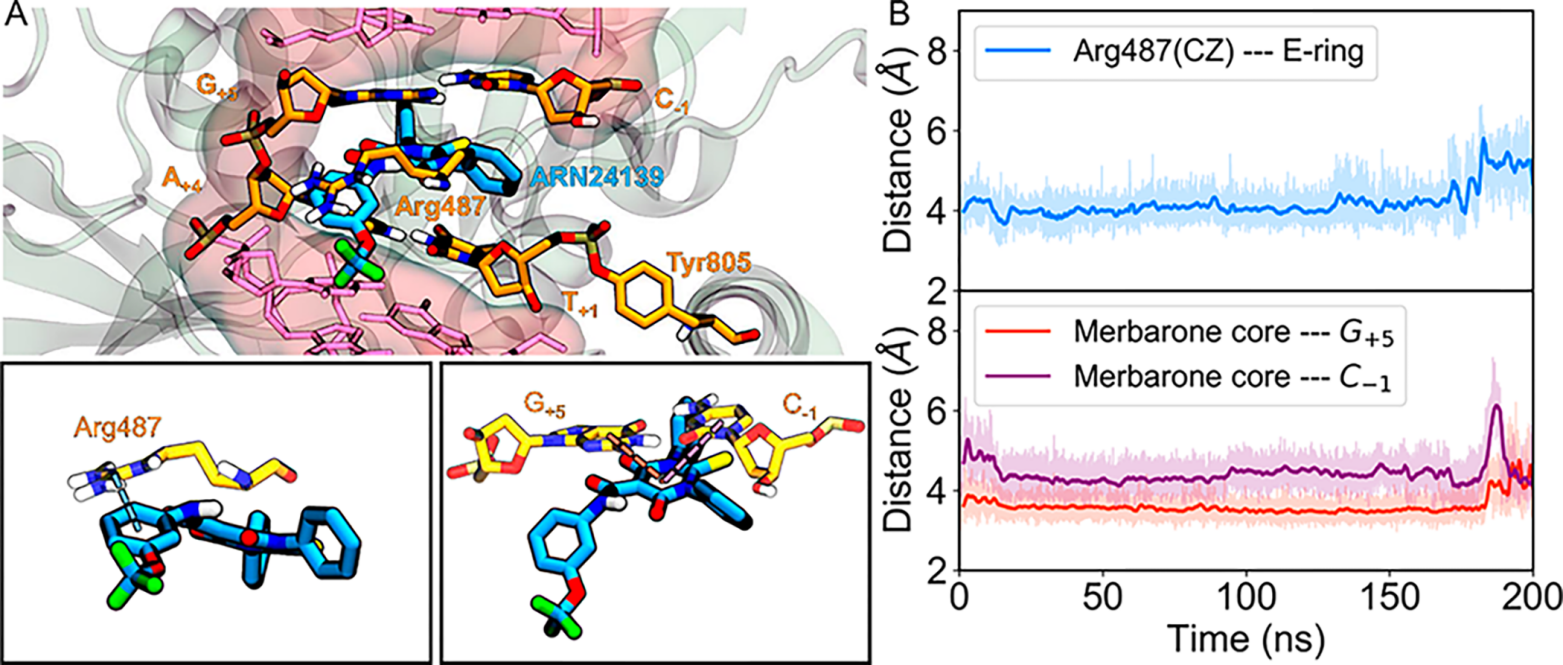
Dynamic description for the binding of **3f** to the in the DNA cleavage/religation active site of topoIIα (PDB code 5GWK). (A) Lead compound in its binding mode. The residues directly interacting with the drug are shown in orange carbons. The rest of the DNA is shown in pink and the protein in white. The insets zoom into the distances of interest, particularly those of Arg487 with the E-ring, and the merbarone core with the G_+5_ and C_–1_ bases. (B) Evolution of the distances of interest over time.

Once the general interaction pattern was established, we proceeded to perform equilibrium molecular dynamics (MD) simulations (∼200 ns) to analyze the evolution and stability of the ternary topoII/DNA/**3f** model system.^14,57−59^ For this, distances representative of the interactions described above were tracked (Figure 4A). The simulations corroborate the role of Arg487 in stabilizing the drug at the cleaved site. Indeed, we monitored the distance between the carbon atom of the guanidinium group and the centroid of the E-ring and found that it remains under 6 Å for 99% of the simulation (Figure 4B).

The H-bonds between the ligand and the cleaved complex were also examined. It is worth noting that the −NH linker present in compound **3f** adds to the rigidity of the molecule. In fact, it enables the formation of a six-membered intramolecular pseudocycle. The cycled configuration was present for ∼79% of the overall simulation time. Nonetheless, the −NH group was found to intermittently invert in order to form an H-bond with the oxygen from the deoxyribose ring of G_+5_. The latter bond persisted for a total of ∼17% of the production run, thus making a complementary interaction for the stabilization of the cleaved complex holo-form.

Similarly, we examined the staggered π-stacking formed between the thiobarbituric cycle and the G_+5_ and C_–1_ bases (Figure 4B). Here, the distance to the G_+5_ base remains consistently smaller at a stable value of 3.6 ± 0.3 Å, whereas the distance to C_–1_ extends to 4.4 ± 0.4 Å. A similar pattern is observed between the phenyl groups and the vicinal base pairs. In summary, the binding mode most frequently visited is stable in our MD simulations too, with key cation−π, H-bonds, and stacking interactions formed with the surrounding DNA/protein complex.

### *In Vivo* Pharmacokinetics

**Figure 5:**
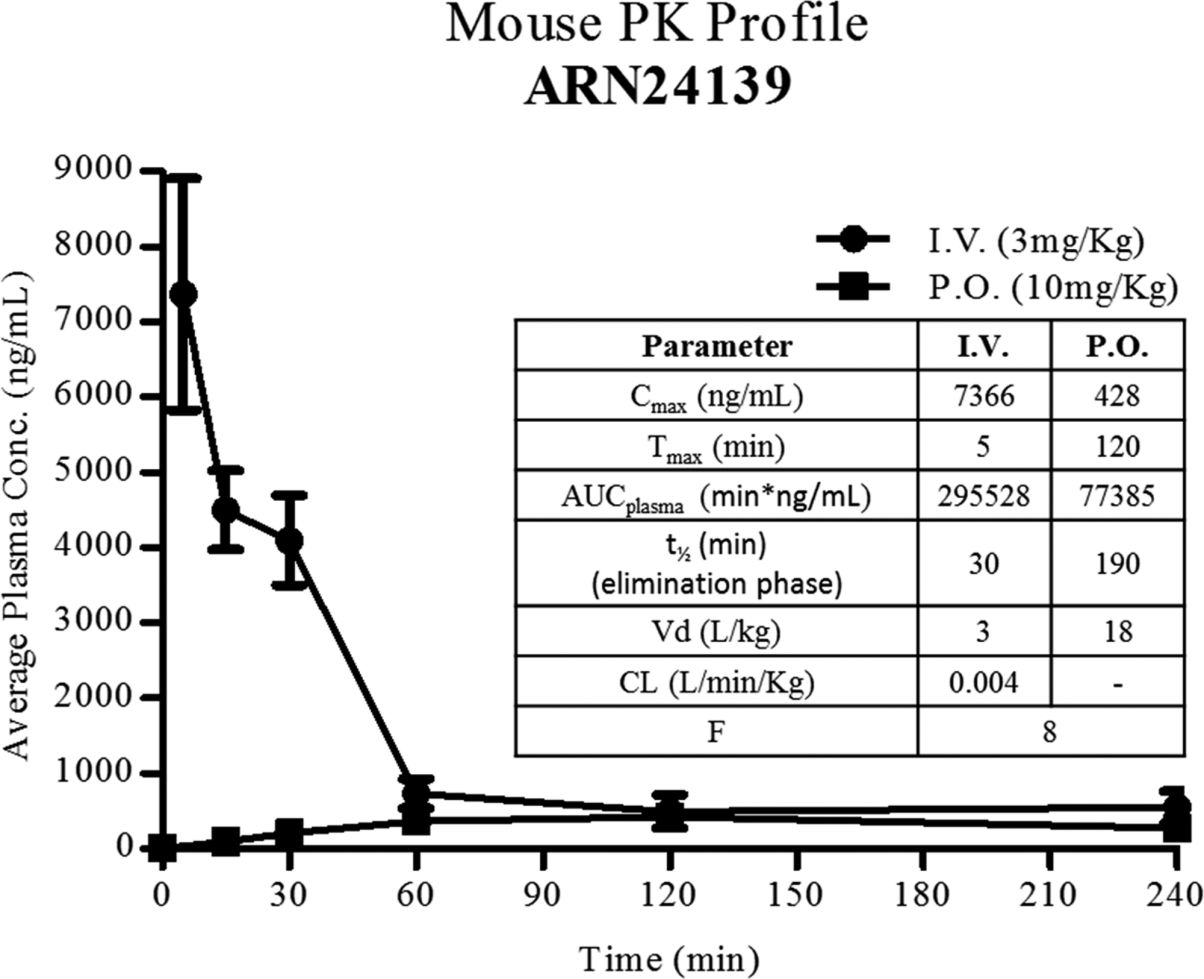
Mouse PK profiles of **3f** following intravenous (iv) and oral administration (po) at 3 and 10 mg/kg, respectively: observed and calculated PK parameters following intravenous (iv) and oral administration (po). The bioavailability *F* was calculated to 8% based on the AUC (area under curve) from *t* = 0 to 240 min.

Finally, on the basis of the overall results and drug-like profile, **3f** was selected as our lead for *in vivo* pharmacokinetics studies, as a preparatory characterization for future analyses of *in vivo* drug efficacy in animal models of cancer. We tested two different routes of administration: (i) intravenous (iv) injection at a concentration of 3 mg/kg (*n* = 3 for each time point) and oral (po) treatment at a dose of 10 mg/kg (*n* = 3 for each time point). Despite the relatively low thermodynamic solubility of **3f** (30 μM in PBS), the compound reached the target concentration in the formulation used for the *in vivo* experiments. The mean plasma concentration versus time profile of **3f** is shown in Figure 5, and the corresponding pharmacokinetic parameters are summarized in the inserted table. During the PK studies, via either iv or po administration, **3f** was well tolerated by all the animals, and no treatment-related clinical signs were observed.

The peak plasma **3f** concentration for iv was observed at the earliest time point (5 min after administration), and the concentration of **3f** in plasma was above the lower limit of quantification throughout the sampling period. The iv profile of **3f** presents a very fast distribution phase with a *C*_max_ of 7366 ng/mL, followed by a slower exposure phase. The compound was still detectable after 2 h at a concentration of 551 ng/mL, with Clp value of 0.004 L min^–1^ kg^–1^. After oral administration (10 mg/kg), plasma concentration of about 400 ng/mL was reached relatively quickly (1 h), and it was stably maintained for at least 6 h. The maximum concentration was achieved at approximately 2 h after oral administration, 428 ng/mL. These data indicate that the compound **3f** is well tolerated. Indeed, the animal behavior and the obtained PK profiles indicate that the dose of **3f** could be increased. This may be beneficial, given the observed very high protein binding of this compound (>99% in both mouse and human plasma) that may limit target engagement.

## Conclusion

On the basis of our previous results on a novel hybrid scaffold with structural elements of merbarone and etoposide,^39^ we have here reported the design, synthesis, and extensive experimental–computational characterization of new hybrid molecules that act as topoII poisons. The resulting SAR elucidated the key structural features that enhanced the potency and antiproliferative activity of our new etoposide–merbarone hybrid compounds. These new compounds were often equipotent and sometimes more potent relative to the reference compounds merbarone and etoposide. Inhibitory activity was improved by introducing a bulkier group in *meta* position of the mimic E- ring (**3a** and **3b**, Scheme 1). Incorporating electron-withdrawing groups preserved or slightly improved the inhibitory activity (**3c**–**e**, Scheme 1), while the bioisosteric substitution with fluorine-embedding groups (**3f**–**h**,Scheme 1) was highly favorable. Furthermore, in the structural design of these new bioactive hybrid molecules, the combined functionalization of both the aromatic E-ring and the hydrocarbon spacer was essential to fine-tuning the drug–target interactions, as proven by the activity of more flexible hybrid topoII poisons (**6**–**8**, and **5d**–**f**, Scheme 2). Taken together, the inhibitory activity and extensive analyses of the drug-likeness profile indicate the novel derivative **3f** (ARN24139) as the most drug-like topoII poison of this novel chemical series. This lead compound has also shown promising antiproliferative activity against cancer cell lines and a favorable pharmacokinetic profile, which are promising features for future *in vivo* efficacy studies.

## Experimental Section

### Chemistry. General Considerations

All the commercially available reagents and solvents were used as purchased from vendors without further purification. Dry solvents were purchased from Sigma-Aldrich. Automated column chromatography purifications were done using a Teledyne ISCO apparatus (CombiFlash Rf) with prepacked silica gel columns of different sizes (from 4 g up to 24 g) and mixtures of increasing polarity of cyclohexane and ethyl acetate (EtOAc) or dichloromethane (DCM) and methanol (MeOH). NMR data were collected on 400 or 600 MHz (1H) and 100 or 150 MHz (^13^C). Spectra were acquired at 300 K, using deuterated dimethyl sulfoxide (DMSO-*d*_6_) or deuterated chloroform (CDCl_3_) as solvents. For ^1^H NMR, data are reported as follows: chemical shift, multiplicity (s = singlet, d = doublet, dd = double of doublets, t = triplet, q = quartet, m = multiplet), coupling constants (Hz), and integration. UPLC/MS analyses were run on a Waters ACQUITY UPLC/MS instrument consisting of an SQD (single quadrupole detector). The analyses were performed on an ACQUITY UPLC BEH C_18_ column (50 × 2.1 mmID, particle size 1.7 μm) with a VanGuard BEH C_18_ precolumn (5 × 2.1 mmID, particle size 1.7 μm) (log *D* > 1). The mobile phase was 10 mM NH_4_OAc in H_2_O at pH 5 adjusted with AcOH (A) and 10 mM NH_4_OAc in MeCN–H_2_O (95:5) at pH 5 (B). Electrospray ionization in positive and negative modes was applied in the mass scan range 100–500 Da. Depending on the analysis method used, a different gradient increasing the proportion of mobile phase B was applied. For analysis method A, the mobile-phase B proportion increased from 5% to 95% in 3 min. For analysis method B, the mobile-phase B proportion increased from 50% to 100% in 3 min. High-resolution mass spectrometry (HRMS) was carried out on a Waters Synapt G2 quadrupole-Tof instrument equipped with an ESI ion source. The analyses were run on an ACQUITY UPLC BEH C_18_ column (50 × 2.1 mmID, particle size 1.7 μm), using H_2_O + 0.1% formic acid (A) and MeCN + 0.1% formic acid as mobile phase. All final compounds displayed ≥95% purity as determined by NMR and UPLC/MS analysis.

### 3-(1,1-Difluoroethyl)aniline (**2h**)

A solution of 3-nitroacetophenone (165 mg, 1 mmol) in dry CH_2_Cl_2_ (4 mL) was treated with [bis(2-methoxyethyl)amino]sulfur trifluoride 50 wt % solution in toluene (2.5 mmol) at room temperature under argon. EtOH (24 μL, 0.4 mmol) was added, and the reaction mixture was stirred at room temperature for 48 h, after which time, the solution was poured into NaHCO_3_ saturated solution and extracted with CH_2_Cl_2_ (2 × 5 mL). Combined organic layers were dried with Na_2_SO_4_, filtered and concentrated under vacuum. Purification by silica gel flash chromatography (elution by gradient from 100 to 95/5 cyclohexane/EtOAc) afforded pure 1-(1,1-difluoroethyl)-3-nitrobenzene (73 mg, 39% yield). ^1^H NMR (400 MHz, CDCl_3_): δ 8.38 (bs, 1H), 8.31 (d, *J* = 7.7 Hz, 1H), 7.85 (d, *J* = 7.7 Hz, 1H), 7.64 (dd, *J* = 7.9, 7.9 Hz, 1H), 1.98 (t, ^3^*J*_H–F_ = 18.2 Hz, 3H). Then tin chloride dihydrate (440 mg, 1.95 mmol) was added to a solution of compound 1-(1,1-difluoroethyl)-3-nitrobenzene (73 mg, 0.39 mmol) in 3 mL of ethanol. The reaction mixture was refluxed for 1 h. The mixture was slowly poured on cooled water. The pH was adjusted to 7 by addition of an aqueous 5 N solution of sodium hydroxide, then adjusted to 9 by addition of an aqueous NaHCO_3_ satured solution. The product was extracted with EtOAc (3 × 5 mL). The organic phases were combined, dried over Na_2_SO_4_, filtered and concentrated under vacuum. Purification by silica gel flash chromatography (elution by gradient from 100 to 80/20 cyclohexane/EtOAc) afforded pure compound **3h** (58 mg, 95% yield). UPLC/MS: Rt = 1.75 min (method A). MS (ESI) *m*/*z*: 158.0 [M + H]^+^, C_8_H_10_F_2_N [M + H]^+^ calculated, 158.1. ^1^H NMR (400 MHz, CDCl_3_): δ 7.19 (dd, *J* = 7.9, 7.9 Hz, 1H), 6.89 (d, *J* = 7.8 Hz, 1H), 6.82 (bs, 1H), 6.73 (d, *J* = 7.5 Hz, 1H), bs (2H), 1.89 (t, ^3^*J*_H–F_= 18.2 Hz, 3H).

### General Procedure 1: Method A for Amide Formation

A solution 0.5 M ethyl 6-hydroxy-4-oxo-1,3-diphenyl-2-thioxo-1,2,3,4-tetrahydropyrimidine-5-carboxylate **1** (1 equiv) and an appropriate aniline (1 equiv) in DMF dry was stirred at 100 °C for 30 min, then cooled to room temperature, and the product was precipitated with water, filtered, and rinsed with MeOH, yielding pure compound.

### General Procedure 2: Method B for Amide Formation

A solution 0.5 M ethyl 6-hydroxy-4-oxo-1,3-diphenyl-2-thioxo-1,2,3,4-tetrahydropyrimidine-5-carboxylate **1** (1 equiv) and an appropriate amine (1.2 equiv) in toluene dry was stirred at 100 °C for 2 h. After completion of reaction, the solvent was removed under vacuum. The product was purified by flash chromatography and/or by trituration with MeOH, yielding pure compound.

### *N*-(3-Ethoxyphenyl)-6-hydroxy-4-oxo-1,3-diphenyl-2-thioxo-1,2,3,4-tetrahydropyrimidine-5-carboxamide (**3a**)

The title compound was prepared according to general procedure 1 using 3-ethoxyaniline **2a** (31 mg, 0.23 mmol), ester **1** (86 mg, 0.23 mmol) in anhydrous DMF (0.46 mL). Then, water (3 mL) was added, and the resulting precipitate was filtered and rinsed with water (2 mL) and MeOH (2 mL), yielding **3a** (70 mg, 66% yield). UPLC/MS: Rt = 1.99 min (method A). MS (ESI) *m*/*z*:.460.4 [M + H]^+^, C_25_H_22_N_3_O_4_S [M + H]^+^ calculated, 460.5. HRMS (AP-ESI) *m*/*z* calcd for C_25_H_22_N_3_O_4_S [M + H]^+^ 460.1331, found 460.1331. ^1^H NMR (400 MHz, DMSO-*d*_6_): δ 11.63 (s, 1H, NH), 7.51–7.33 (m, 10H, Ph), 7.29 (dd, *J* = 8.1, 8.1 Hz, 1H, Ar), 7.14 (dd, *J* = 2.2, 2.2 Hz, 1H, Ar), 7.10 (dd, *J* = 8.0, 1.9 Hz, 1H, Ar), 6.80 (dd, *J* = 8.2, 2.3 Hz, 1H, Ar), 4.02 (q, *J* = 7.0 Hz, 2H), 1.30 (t, *J* = 6.9 Hz, 3H). ^13^C (100 MHz, DMSO-*d*_6_): δ 178.3 (CS), 168.7 (Cq), 159.0 (Cq), 139.0 (Cq), 136.4 (Cq), 130.1 (CH), 129.1 (CH), 129.0 (CH), 128.4 (CH), 114.0 (CH), 112.1 (CH), 108.1 (CH), 84.0 (Cq), 63.3 (CH_2_), 14.6 (CH_3_).

### 6-Hydroxy-*N*-(3-isopropoxyphenyl)-4-oxo-1,3-diphenyl-2-thioxo-1,2,3,4-tetrahydropyrimidine-5-carboxamide (**3b**)

The title compound was prepared according to general procedure 1 using 3-isopropyloxyaniline **2b** (45 mg, 0.30 mmol), ester **1** (110 mg, 0.30 mmol) in anhydrous DMF (0.6 mL). Then, water (3 mL) was added, and the resulting precipitate was filtered and rinsed with water (2 mL) and MeOH (2 mL), yielding **3b** (50 mg, 35% yield). UPLC/MS: Rt = 2.10 min (method A). MS (ESI) *m*/*z*: 472.4 [M – H]^−^, C_26_H_22_N_3_O_4_S [M – H]^−^ calculated, 472.5. HRMS (AP-ESI) *m*/*z* calcd for C_26_H_24_N_3_O_4_S [M + H]^+^ 474.1488, found 474.1494. ^1^H NMR (400 MHz, CDCl_3_): δ 11.85 (s, 1H, NH), 7.50–7.60 (m, 6H, Ph), 7.36–7.33 (m, 4H, Ph), 7.27 (dd, *J* = 8.0, 5.0 Hz, 1H, Ar), 7.08–7.04 (m, 2H, Ar), 6.79 (dd, *J* = 8.3, 2.4 Hz, 1H, Ar), 4.54 (quint, *J* = 6.0 Hz, 1H), 1.36 (d, *J* = 6.0 Hz, 6H). ^13^C (100 MHz, CDCl_3_): δ 178.6 (CS), 169.3 (Cq), 167.8 (Cq), 162.3 (Cq), 158.7 (Cq), 139.2 (Cq), 138.1 (Cq), 136.2 (Cq), 130.2 (CH), 129.8 (CH), 129.7 (CH), 129.3 (CH), 129.1 (CH), 128.8 (CH), 128.6 (CH), 114.2 (CH), 114.0 (CH), 109.5 (CH), 83.6 (Cq), 70.3 (CH), 22.1 (CH_3_).

### *N*-(3-Cyanophenyl)-6-hydroxy-4-oxo-1,3-diphenyl-2-thioxo-1,2,3,4-tetrahydropyrimidine-5-carboxamide (**3c**)

The title compound was prepared according to general procedure 1 using 3-cianoaniline **2c** (48 mg, 0.41 mmol), ester **1** (150 mg, 0.41 mmol) in anhydrous DMF (0.82 mL). Then, water (4 mL) was added, and the resulting precipitate was filtered and rinsed with water (4 mL) and MeOH (4 mL), yielding **3c** (122 mg, 68% yield) as a light pink amorphous solid. UPLC/MS: Rt = 1.75 min (method A). MS (ESI) *m*/*z*: 441.4 [M + H]^+^, C_24_H_17_N_4_O_3_S [M + H]^+^ calculated, 441.4. HRMS (AP-ESI) *m*/*z* calcd for C_24_H_17_N_4_O_3_S [M + H]^+^ 441.1021, found 441.1025. ^1^H NMR (400 MHz, DMSO-*d*_6_): δ 11.72 (s, 1H, NH), 8.10 (bs, 1H, Ar), 7.86 (d, *J* = 8.2 Hz, 1H, Ar), 7.66 (d, *J* = 7.7 Hz, Ar), 7.58 (dd, *J* = 8.0, 8.0 Hz, 1H, Ar), 7.51–7.33 (m, 10H, Ph). ^13^C (100 MHz, DMSO-*d*_6_): δ 178.2 (CS), 168.7 (Cq), 164.1 (Cq), 139.1 (Cq), 138.8 (Cq), 130.5 (CH), 129.1 (CH), 129.0 (CH), 128.9 (CH), 126.8 (CH), 125.1 (CH), 118.3 (Cq), 111.9 (Cq), 84.7 (Cq).

### 6-Hydroxy-*N*-(3-nitrophenyl)-4-oxo-1,3-diphenyl-2-thioxo-1,2,3,4-tetrahydropyrimidine-5-carboxamide (**3d**)

The title compound was prepared according to general procedure 1 using 3-nitroaniline **2d** (38 mg, 0.27 mmol), ester **1** (100 mg, 0.27 mmol) in anhydrous DMF (0.54 mL). Then, water (4 mL) was added, and the resulting precipitate was filtered and rinsed with water (4 mL) and MeOH (4 mL), yielding **3d** (70 mg, 57% yield) as a pale yellow amorphous solid. UPLC/MS: Rt = 1.86 min (method A). MS (ESI) *m*/*z*: 461.5 [M + H]^+^, C_23_H_17_N_4_O_5_S [M + H]^+^ calculated, 461.5. HRMS (AP-ESI) *m*/*z* calcd for C_23_H_17_N_4_O_5_S [M + H]^+^ 461.0920, found 461.0924. ^1^H NMR (400 MHz, DMSO-*d*_6_): δ 11.84 (s, 1H, NH), 8.59 (dd, *J* = 2.2, 2.2 Hz, 1H, Ar), 8.01 (ddd, *J* = 8.2, 2.3, 0.8 Hz, 1H, Ar), 7.89 (ddd, *J* = 8.2, 2.1, 0.8 Hz, 1H, Ar), 7.64 (dd, *J* = 8.2, 8.2 Hz, 1H, Ar), 7.50–7.31 (m, 10H, Ph). ^13^C NMR (100 MHz, CDCl_3_): δ 178.2 (CS), 169.9 (Cq), 168.1 (Cq), 162.2 (Cq), 148.9 (Cq), 138.9 (Cq), 137.6 (Cq), 136.8 (Cq), 130.3 (CH), 129.9 (CH), 129.8 (CH), 129.6 (CH), 129.3 (CH), 128.6 (CH), 128.5 (CH), 127.0 (CH), 120.6 (CH), 116.6 (CH), 84.1 (Cq).

### *N*-(3-Fluorophenyl)-6-hydroxy-4-oxo-1,3-diphenyl-2-thioxo-1,2,3,4-tetrahydropyrimidine-5-carboxamide (**3e**)

The title compound was prepared according to general procedure 1 using 3-fluoroaniline **2e** (48 μL, 0.50 mmol), ester **1** (184 mg, 0.50 mmol) in anhydrous DMF (1 mL). Then, water (5 mL) was added, and the resulting precipitate was filtered and rinsed with water (5 mL) and MeOH (5 mL), yielding **3e** (82 mg, 38% yield) as a white amorphous solid. UPLC/MS: Rt = 1.86 min (method A). MS (ESI) *m*/*z*: 434.3 [M + H]^+^, C_23_H_17_FN_3_O_3_S [M + H]^+^ calculated, 434.5. HRMS (AP-ESI) *m*/*z* calcd for C_23_H_17_FN_3_O_3_S [M + H]^+^ 434.0975, found 434.0974. ^1^H NMR (600 MHz, CDCl_3_): δ 11.91 (s, 1H, NH), 7.59–7.56 (m, 4H, Ar), 7.53–7.49 (m, 2H, Ar), 7.41 (ddd, *J* = 10.4, 2.3, 2.3 Hz, 1H, Ar), 7.35 (m, 5H), 7.18 (dd, *J* = 8.8, 2.0 Hz, 1H, Ar), 6.94 (ddd, *J* = 8.2, 8.2, 2.5 Hz, 1H, Ar). ^13^C NMR (150 MHz, CDCl_3_): δ 178.4 (CS), 169.5 (Cq), 167.9 (Cq), 163.8 (Cq), 163.0 (d, ^1^*J*_CF_ = 246.3 Hz, Cq), 139.1 (Cq), 137.9 (Cq), 136.8 (d, ^3^*J*_CF_ = 10.8 Hz, Cq), 130.6 (d, ^3^*J*_CF_ = 9.1 Hz, CH), 129.9 (CH), 129.8 (CH), 129.4 (CH), 129.2 (CH), 128.7 (CH), 128.5 (CH), 117.2 (d, ^4^*J*_CF_ = 3.0 Hz, CH), 113.2 (^2^*J*_CF_ = 21.5 Hz, CH), 109.4 (CH, ^2^*J*_CF_ = 21.4 Hz, CH), 83.8 (Cq). ^19^F NMR (565 MHz): δ −110.3 (s).

### 6-Hydroxy-4-oxo-1,3-diphenyl-2-thioxo-*N*-(3-(trifluoromethoxy)phenyl)-1,2,3,4-tetrahydropyrimidine-5-carboxamide (**3f**)

The title compound was prepared according to general procedure 1 using 3-trifluoromethoxyaniline **2f** (54 μL, 0.41 mmol), ester **1** (150 mg, 0.41 mmol) in anhydrous DMF (0.82 mL). Then, water (4 mL) was added, and the resulting precipitate was filtered and rinsed with water (4 mL) and MeOH (4 mL), yielding **3f** (79 mg, 39% yield) as a white amorphous solid. UPLC/MS: Rt = 2.05 min (method A). MS (ESI) *m*/*z*: 500.3 [M + H]^+^, C_24_H_17_F_3_N_3_O_4_S [M + H]^+^ calculated, 500.5. HRMS (AP-ESI) *m*/*z* calcd for C_24_H_17_F_3_N_3_O_4_S [M + H]^+^ 500.0892, found 500.0883. ^1^H NMR (400 MHz, DMSO-*d*_6_): δ 11.70 (s, 1H, NH), 7.71 (s, 1H, Ar), 7.54–7.31 (m, 12H, Ar), 7.19 (d, *J* = 7.7 Hz, 1H, Ar). ^13^C NMR (150 MHz, CDCl_3_): δ 178.4 (CS), 169.6 (Cq), 168.0 (Cq), 162.2 (Cq), 149.7 (Cq), 139.0 (Cq), 137.8 (Cq), 136.8 (Cq), 130.6 (CH), 129.9 (CH), 129.8 (CH), 129.5 (CH), 129.2 (CH), 128.7 (CH), 128.5 (CH), 120.5 (q, ^1^*J*_CF_ = 258 Hz, Cq), 119.9 (CH), 118.4 (CH), 114.6 (CH), 83.8 (Cq). ^19^F NMR (565 MHz): δ −57.8 (s).

### *N*-(3-(Difluoromethoxy)phenyl)-6-hydroxy-4-oxo-1,3-diphenyl-2-thioxo-1,2,3,4-tetrahydropyrimidine-5-carboxamide (**3g**)

The title compound was prepared according to general procedure 1 using 3-(difluoromethoxy)aniline **2g** (51 μL, 0.41 mmol), ester **1** (150 mg, 0.41 mmol) in anhydrous DMF (0.82 mL). Then, water (4 mL) was added, and the resulting precipitate was filtered and rinsed with water (4 mL) and MeOH (4 mL), yielding **3g** (67 mg, 34% yield) as a white amorphous solid. UPLC/MS: Rt = 1.95 min (method A). MS (ESI) *m*/*z*: 482.4 [M + H]^+^, C_24_H_18_F_2_N_3_O_3_S [M + H]^+^ calculated, 482.5. HRMS (AP-ESI) *m*/*z* calcd for C_24_H_18_F_2_N_3_O_3_S [M + H]^+^ 482.0986, found 482.0984. ^1^H NMR (600 MHz, CDCl_3_): δ 11.9 (s, 1H, NH), 7.58–7.50 (m, 6H, Ar), 7.38–7.31 (m, 7H), 7.00 (d, *J* = 8.1 Hz, 1H, Ar), 6.52 (t, *J* = 73.4 Hz, 1H, CHF_2_). ^13^C NMR (150 MHz, CDCl_3_): δ 178.4 (CS), 169.6 (Cq), 167.9 (Cq), 162.2 (Cq), 151.6 (Cq), 139.1 (Cq), 137.9 (Cq), 136.7 (Cq), 130.6 (CH), 129.9 (CH), 129.8 (CH), 129.5 (CH), 129.2 (CH), 128.7 (CH), 128.5 (CH), 118.6 (CH), 117.1 (CH), 115.7 (t, ^1^*J*_CF_ = 260.5 Hz, CH), 113.3 (CH), 83.8 (Cq). ^19^F NMR (565 MHz): δ −81.2 (s).

### *N*-(3-(1,1-Difluoroethyl)phenyl)-6-hydroxy-4-oxo-1,3-diphenyl-2-thioxo-1,2,3,4-tetrahydropyrimidine-5-carboxamide (**3h**)

The title compound was prepared according to general procedure 1 using 3-(1,1-difluoroethyl)aniline **2h** (50 mg, 0.32 mmol), ester **1** (117 mg, 0.32 mmol) in anhydrous DMF (0.64 mL). Then, water (3 mL) was added, and the resulting precipitate was filtered and rinsed with water (3 mL) and MeOH (3 mL), yielding **3h** (104 mg, 68% yield) as a white amorphous solid. UPLC/MS: Rt = 2.08 min (method B). MS (ESI) *m*/*z*: 480.1 [M + H]^+^, C_25_H_20_F_2_N_3_O_3_S [M + H]^+^ calculated, 480.5. HRMS (AP-ESI) *m*/*z* calcd for C_25_H_20_F_2_N_3_O_3_S [M + H]^+^ 480.1193, found 480.1194. ^1^H NMR (400 MHz, CDCl_3_): δ 11.95 (s, 1H, NH), 7.63–7.47 (m, 8H, Ar), 7.44 (dd, *J* = 7.9, 7.9 Hz, 1H, Ar), 7.37 (d, *J* = 7.9 Hz, 1H, Ar), 7.33–7.29 (m, 4H), 1.90 (t, *J* = 18.2 Hz, 3H). ^13^C (100 MHz, CDCl_3_): δ 178.5 (CS), 169.5 (Cq), 167.9 (Cq), 162.3 (Cq), 139.7 (t, ^2^*J*_CF_ = 27.3 Hz, Cq), 139.1 (Cq), 137.9 (Cq), 135.6 (Cq), 129.9 (CH), 129.8 (CH), 129.5 (CH), 129.2 (CH), 128.7 (CH), 122.9 (CH), 122.5 (t, ^3^*J*_CF_ = 5.9 Hz, CH), 121.3 (t, ^1^*J*_CF_ = 239.5 Hz, CF_2_), 118.2 (t, ^3^*J*_CF_= 6.4 Hz, CH), 83.7 (Cq), 26.0 (t, ^2^*J*_CF_ = 29.5 Hz, CH_3_). ^19^F NMR (565 MHz): δ −87.1 (s).

### *N*-(3-(Difluoromethyl)phenyl)-6-hydroxy-4-oxo-1,3-diphenyl-2-thioxo-1,2,3,4-tetrahydropyrimidine-5-carboxamide (**3i**)

The title compound was prepared according to general procedure 1 using 3-(difluoromethyl)aniline **2i** (43 mg, 0.30 mmol), ester **1** (110 mg, 0.30 mmol) in anhydrous DMF (0.60 mL). Then, water (3 mL) was added, and the resulting precipitate was filtered and rinsed with water (3 mL) and MeOH (3 mL), yielding **3i** (45 mg, 32% yield) as a white amorphous solid. UPLC/MS: Rt = 1.94 min (method A). MS (ESI) *m*/*z*: 466.4 [M + H]^+^, C_24_H_18_F_2_N_3_O_3_S [M + H]^+^ calculated, 466.5. HRMS (AP-ESI) *m*/*z* calcd for C_24_H_18_F_2_N_3_O_3_S [M + H]^+^ 466.1037, found 466.1041. ^1^H NMR (600 MHz, CDCl_3_): δ 11.97 (s, 1H, NH), 7.68 (s, 1H), 7.61–7.47 (m, 8H, Ar), 7.38–7.31 (m, 5H, Ar), 6.63 (t, *J* = 56.5 Hz, 1H, CHF_2_). ^13^C (150 MHz, CDCl_3_): δ 178.5 (CS), 169.6 (Cq), 167.9 (Cq), 162.2 (Cq), 139.1 (Cq), 137.9 (Cq), 135.9 (Cq), 135.8 (t, ^2^*J*_CF_ = 22.6 Hz, Cq), 130.0 (CH), 129.9 (CH), 129.8 (CH), 129.5 (CH), 129.2 (CH), 128.7 (CH), 128.5 (CH), 123.8 (CH), 123.3 (t, ^3^*J*_CF_ = 6.1 Hz, CH), 119.0 (t, ^3^*J*_CF_ = 6.1 Hz, CH), 119.0 (t, ^3^*J*_CF_ = 6.2 Hz, CH), 114.0 (t, ^1^*J*_CF_ = 240.8 Hz, CH), 83.8 (Cq). ^19^F NMR (565 MHz): δ −111.5 (s).

### *N*-(4-Fluoro-3-methylphenyl)-6-hydroxy-4-oxo-1,3-diphenyl-2-thioxo-1,2,3,4-tetrahydropyrimidine-5-carboxamide (**3j**)

The title compound was prepared according to general procedure 2 using 4-fluoro-3-methoxyaniline **2j** (61 mg, 0.43 mmol), ester **1** (132 mg, 0.36 mmol) in anhydrous toluene (0.72 mL). Then, the solvent was removed under vacuum, the residue was treated with water (3 mL), and the resulting precipitate was filtered and rinsed with MeOH (3 mL), yielding **3j** (126 mg, 72% yield) as a white amorphous solid. UPLC/MS: Rt = 1.94 min (method A). MS (ESI) *m*/*z*: 464.4 [M + H]^+^, C_24_H_19_FN_3_O_4_S [M + H]^+^ calculated, 464.5. HRMS (AP-ESI) *m*/*z* calcd for C_24_H_19_FN_3_O_4_S [M + H]^+^ 464.1080, found 464.1084. ^1^H NMR (600 MHz, CDCl_3_): δ 11.84 (s, 1H, NH), 7.59–7.49 (m, 6H, Ar), 7.34–7.31 (m, 4H), 7.09–7.06 (m, 3H). ^13^C (150 MHz, CDCl_3_): δ 178.5 (CS), 169.1 (Cq), 167.8 (Cq), 162.3 (Cq), 150.4 (^1^*J*_CF_ = 245.4 Hz, Cq), 148.1 (^2^*J*_CF_ = 11.3 Hz, Cq), 139.1 (Cq), 138.0 (Cq), 131.5 (d, ^4^*J*_CF_ = 3.3, Cq), 129.9 (CH), 129.8 (CH), 129.4 (CH), 129.1 (CH), 128.7 (CH), 128.5 (CH), 116.5 (d, ^2^*J*_CF_ = 19.6 Hz, CH), 114.1 (d, ^3^*J*_CF_ = 6.8 Hz, CH), 107.6 (CH), 83.6 (Cq), 56.4 (OCH_3_). ^19^F NMR (565 MHz): δ −136.7.

### [4-[*tert*-Butyl(dimethyl)silyl]oxy-3,5-dimethoxy-phenyl]methanamine (**4a**)

A suspension of compound **10** (250 mg, 0.77 mmol) in 7 mL of dry MeOH was cooled to 0 °C and treated with NiCl_2_·6H_2_O (734.0 mg, 3.09 mmol). The resulting mixture was stirred at the same temperature for 5 min before the addition of NaBH_4_ (290 mg, 7.66 mmol). After 30 min, the reaction mixture was quenched with saturated aqueous NH_4_Cl (10 mL) solution and extracted with EtOAc (3 × 15 mL). The combined extracts were dried over Na_2_SO_4_ and concentrated under vacuum. Flash chromatographic purification (elution by gradient from 100 to 80/20 DCM/MeOH·NH_3_ 1 N) afforded compound **4a** (110 mg, 48% yield) as a viscous oil. UPLC/MS: Rt = 0.94 min (method B). MS (ESI) *m*/*z*: 281 of main fragment. ^1^H NMR (400 MHz, CDCl_3_): δ 6.51 (s, 2H), 3.80 (bs, 2H), 3.79 (s, 6H), 1.00 (s, 9H, *t*Bu TBS), 0.12 (s, 6H, CH_3_ TBS).

### 2-[4-[*tert*-Butyl(dimethyl)silyl]oxy-3,5-dimethoxyphenyl]ethanamine (**4b**)

A suspension of compound **11** (250 mg, 0.74 mmol) in 7 mL of dry MeOH was cooled to 0 °C and treated with NiCl_2_·6H_2_O (703.6 mg, 2.96 mmol). The resulting mixture was stirred at the same temperature for 5 min before the addition of NaBH_4_ (279.9 mg, 7.40 mmol). After 30 min, the reaction mixture was quenched with saturated aqueous NH_4_Cl (10 mL) solution and extracted with EtOAc (3 × 15 mL). The combined extracts were dried over Na_2_SO_4_ and concentrated under vacuum. Flash chromatographic purification (elution by gradient from 100 to 80/20 DCM/MeOH·NH_3_ 1 N) afforded compound **4b** (110 mg, 48% yield) as a viscous oil. UPLC/MS: Rt = 1.16 min (method B). MS (ESI) *m*/*z*: 312.2 [M + H]^+^, C_16_H_30_NO_3_Si [M + H]^+^ calculated, 312.2. ^1^H NMR (400 MHz, DMSO-*d*_6_): δ 6.45 (s, 2H), 3.75 (s, 6H), 2.75 (m, 2H), 2.55 (m, 2H), 1.00 (s, 9H, *t*Bu TBS), 0.12 (s, 6H, CH_3_ TBS).

### (*E*)-3-[4-[*tert*-Butyl(dimethyl)silyl]oxy-3,5-dimethoxyphenyl]prop-2-en-1-amine (**4c**)

A 2 M solution of LiALH_4_ in THF (1.37 mL, 2.73 mmol) was added to a suspension of AlCl_3_ (363 mg, 2.73 mmol) in THF dry (6 mL) at 0 °C under argon. After 10 min, a solution of intermediate **13** (774 mg, 0.78 mmol) in 5 mL of dry THF was added dropwise. The mixture was stirred for 30 min at 50 °C and then cooled at 0 °C, quenched with ice–water (5 mL). The pH was adjusted to 9–10 with NaOH 2 M solution. The mixture was extracted with EtOAc (3 × 20 mL). The combined organic extracts were dried over Na_2_SO_4_ and concentrated under vacuum. The ^1^H NMR of the crude of the reaction showed the presence of *E*/*Z* isomers in ratio 1:0.12. Flash chromatographic purification (elution by gradient from 100 to 80/20 DCM/MeOH–NH_3_ 1 N) afforded compound (*E*)-**4c** (90 mg, 35% yield) as a viscous oil. UPLC/MS: Rt = 1.26 min (method B). MS (ESI) *m*/*z*: 307.2 main fragment. ^1^H NMR (400 MHz, CDCl_3_): δ 6.58 (s, 2H), 6.41 (dd, *J* = 16.0, 1.5 Hz, 1H), 6.20 (ddd, *J* = 15.8, 6.0, 6.0 Hz, 1H), 3.80 (s, 6H), 3.47 (dd, *J* = 6.0, 1.4 Hz, 2H), 1.00 (s, 9H, *t*Bu TBS), 0.12 (s, 6H, CH_3_ TBS).

### (*E*)-2-(3-Methoxyphenyl)ethenamine (**4f**)

A 2 M solution of LiAlH_4_ in THF (1.75 mL, 3.5 mmol) was added to a suspension of AlCl_3_ (467 mg, 3.5 mmol) in anhydrous THF (8 mL) at 0 °C under argon. After 10 min, a solution of intermediate **14** (159 mg, 1.0 mmol) in 6 mL of anhydrous THF was added dropwise. The mixture was stirred for 30 min at 50 °C and then cooled at 0 °C, quenched with ice–water (7 mL). The pH was adjusted to 9–10 with NaOH 2 M solution. The mixture was extracted with EtOAc (3 × 20 mL). The combined organic extracts were dried over Na_2_SO_4_ and concentrated under vacuum. The ^1^H NMR of the crude of the reaction showed the presence of *E*/*Z* isomers in ratio 1:0.10. Flash chromatographic purification (elution by gradient from 100 to 80/20 DCM/MeOH–NH_3_ 1 N) yielded title compound (*E*)-**4f** (45 mg, 27% yield) as a yellow viscous oil. UPLC/MS: Rt = 1.15 min (method A). MS (ESI) *m*/*z*: 147.0 main fragment. ^1^H NMR (400 MHz, CDCl_3_): δ 7.22 (dd, *J* = 7.9, 7.9 Hz, 1H), 6.97 (d, *J* = 7.8 Hz, 1H), 6.91 (dd, *J* = 2.0, 2.0 Hz, 1H), 6.78 (dd, *J* = 8.1, 2.6 Hz, 1H), 6.48 (ddd, *J* = 15.9, 1.7, 1.7 Hz, 1H), 6.32 (ddd, *J* = 15.8, 5.8, 5.8 Hz, 1H), 3.81 (s, 3H), 3.49 (dd, *J* = 5.8, 1.3 Hz, 2H).

### *N*-(4-((*tert*-Butyldimethylsilyl)oxy)-3,5-dimethoxybenzyl)-6-hydroxy-4-oxo-1,3-diphenyl-2-thioxo-1,2,3,4-tetrahydropyrimidine-5-carboxamide (**5a**)

The title compound was prepared according to general procedure 2 using amine **4a** (80 mg, 0.27 mmol), ester **1** (83 mg, 0.22 mmol) in anhydrous toluene (0.44 mL). Then, the solvent was removed under vacuum. Flash chromatographic purification (elution by gradient from 100 to 85/15 cyclohexane/EtOAc) afforded **5a** (95 mg, 70% yield) as a viscous oil. UPLC/MS: Rt = 2.08 min (method B). MS (ESI) *m*/*z*: 620.3 [M + H]^+^, C_32_H_38_N_3_O_6_SSi [M + H]^+^ calculated, 620.8. ^1^H NMR (400 MHz, CDCl_3_): δ 10.13 (dd, *J* = 5.8, 5.8 Hz, 1H, NH), 7.55–7.42 (m, 6H, Ph), 7.30–7.23 (m, 4H, Ph), 6.44 (s, 2H, Ar), 4.48 (d, *J* = 5.9 Hz, 2H), 3.76 (s, 6H), 1.00 (s, 9H), 0.12 (s, 6H). ^13^C NMR (100 MHz, CDCl_3_): δ 179.0 (CS), 170.4 (CONH), 167.5 (Cq), 162.2 (Cq), 152.0 (Cq), 128.7 (CH), 127.8 (CH), 105.8 (CH), 83.1 (Cq), 56.0 (OCH_3_), 45.0 (CH_2_), 25.9 (CH_3_, TBS), 18.8 (Cq, TBS), −4.5 (CH_3_, TBS).

### *N*-(4-((*tert*-Butyldimethylsilyl)oxy)-3,5-dimethoxyphenethyl)-6-hydroxy-4-oxo-1,3-diphenyl-2-thioxo-1,2,3,4-tetrahydropyrimidine-5-carboxamide (**5b**)

The title compound was prepared according to general procedure 2 using amine **4b** (40 mg, 0.12 mmol), ester **1** (37 mg, 0.10 mmol) in anhydrous toluene (0.50 mL). Then, the solvent was removed under vacuum. Flash chromatographic purification (elution by gradient from 100 to 75/25 cyclohexane/EtOAc) afforded **5b** (27 mg, 42% yield) as a viscous oil. UPLC/MS: Rt = 2.38 min (method B). MS (ESI) *m*/*z*: 634.2 [M + H]^+^, C_33_H_40_N_3_O_6_SSi [M + H]^+^ calculated, 634.8. ^1^H NMR (400 MHz, CDCl_3_): δ 10.04 (dd, *J* = 5.7, 5.7 Hz, 1H, NH), 7.54–7.43 (m, 6H, Ph), 7.29–7.22 (m, 4H, Ph), 6.35 (s, 2H, Ar), 3.71 (s, 6H), 3.65 (ddd, *J* = 6.8, 6.8, 6.8 Hz, 2H), 2.80 (dd, *J* = 6.8, 6.8 Hz, 2H), 1.00 (s, 9H), 0.10 (s, 6H).

### (*E*)-*N*-(3-(4-((*tert*-Butyldimethylsilyl)oxy)-3,5-dimethoxyphenyl)allyl)-6-hydroxy-4-oxo-1,3-diphenyl-2-thioxo-1,2,3,4-tetrahydropyrimidine-5-carboxamide (**5c**)

The title compound was prepared according to general procedure 2 using amine **4c** (65 mg, 0.20 mmol), ester **1** (63 mg, 0.17 mmol) in anhydrous toluene (0.34 mL). Then, the solvent was removed under vacuum. Flash chromatographic purification (elution by gradient from 100 to 75/25 cyclohexane/EtOAc) afforded **5c** (43 mg, 39% yield) as a viscous oil. UPLC/MS: Rt = 2.30 min (method B). MS (ESI) *m*/*z*: 646.3 [M + H]^+^, C_34_H_39_N_3_O_6_SSi [M + H]^+^ calculated, 646.8. ^1^H NMR (400 MHz, CDCl_3_): δ 10.06 (dd, *J* = 5.3, 5.3 Hz, 1H, NH), 7.55–7.42 (m, 6H, Ph), 7.30–7.26 (m, 4H, Ph), 6.53 (s, 2H, Ar), 6.47 (ddd, *J* = 15.7, 1.4, 1.4 Hz, 1H), 6.02 (ddd, *J* = 15.7, 6.5, 6.5 Hz, 1H), 4.17 (ddd, *J* = 6.5, 6.5, 1.4 Hz, 2H), 3.79 (s, 6H), 1.00 (s, 9H), 0.12 (s, 6H). ^13^C NMR (100 MHz, CDCl_3_): δ 179.0 (CS), 170.6 (CONH), 167.4 (Cq), 162.3 (Cq), 151.8 (Cq), 139.4 (Cq), 138.4 (Cq), 134.7 (CH), 129.7 (CH), 129.2 (CH), 128.9 (CH), 128.6 (CH), 103.9 (CH, 2C), 83.1 (Cq), 55.9 (OCH_3_), 42.6 (CH_2_), 25.9 (CH_3_, TBS), 18.9 (Cq, TBS), −4.5 (CH_3_, TBS)

### 6-Hydroxy-*N*-(3-methoxybenzyl)-4-oxo-1,3-diphenyl-2-thioxo-1,2,3,4-tetrahydropyrimidine-5-carboxamide (**5d**)

The title compound was prepared according to general procedure 2 using 3-methoxybenzylamine **4d** (21 μL, 0.16 mmol), ester **1** (50 mg, 0.14 mmol) in anhydrous toluene (0.28 mL). Then, the solvent was removed under vacuum. Flash chromatographic purification (elution by gradient from 100 to 75/25 cyclohexane/EtOAc) afforded **5d** (39 mg, 62% yield) as an amorphous white solid. UPLC/MS: Rt = 2.26 min (method A). MS (ESI) *m*/*z*: 460.2 [M + H]^+^, C_25_H_22_N_3_O_4_S [M + H]^+^ calculated, 460.5. HRMS (AP-ESI) *m*/*z* calcd for C_25_H_22_N_3_O_4_S [M + H]^+^ 460.1331, found 460.1325. ^1^H NMR (400 MHz, DMSO-*d*_6_): δ 10.26 (dd, *J* = 6.0, 6.0 Hz, 1H, NH), 7.47–7.35 (m, 6H, Ar), 7.30–7.25 (m, 5H, Ar), 6.91–6.85 (m, 3H, Ar), 4.55 (d, *J* = 6.2 Hz, 2H), 3.73 (s, 3H). ^13^C (100 MHz, DMSO-*d*_6_): δ 178.6 (CS), 169.7 (Cq), 159.4 (Cq), 139.3 (Cq), 138.8 (Cq), 129.8 (CH), 129.0 (CH), 128.2 (CH), 119.7 (CH), 113.6 (CH), 112.7 (CH), 82.8 (Cq), 55.1 (OCH_3_), 43.7 (CH_2_).

### 6-Hydroxy-*N*-(3-methoxyphenethyl)-4-oxo-1,3-diphenyl-2-thioxo-1,2,3,4-tetrahydropyrimidine-5-carboxamide (**5e**)

The title compound was prepared according to general procedure 2 using 3-methoxyphenethylamine **4e** (38 μL, 0.26 mmol), ester **1** (80 mg, 0.22 mmol) in anhydrous toluene (0.44 mL). Then, the solvent was removed under vacuum. Flash chromatographic purification (elution by gradient from 100 to 75/25 cyclohexane/EtOAc) afforded **5e** (43 mg, 41% yield) as an amorphous white solid. UPLC/MS: Rt = 2.37 min (method A). MS (ESI) *m*/*z*: 474.1 [M + H]^+^, C_26_H_24_N_3_O_4_S [M + H]^+^ calculated, 474.1. HRMS (AP-ESI) *m*/*z* calcd for C_26_H_24_N_3_O_4_S [M + H]^+^ 474.1488, found 474.1489. ^1^H NMR (400 MHz, CDCl_3_): δ 10.03 (dd, J = 6.0, 6.0 Hz, 1H, NH), 7.54–7.43 (m, 6H, Ar), 7.29–7.20 (m, 5H, Ar), 6.79–6.72 (m, 3H, Ar), 3.76 (s, 3H), 3.67 (ddd, *J* = 6.9, 6.9, 6.9 Hz, 2H), 2.87 (dd, *J* = 7.2 Hz, 2H). ^13^C (150 MHz, CDCl_3_): δ 179.0 (CS), 170.8 (Cq), 167.3 (Cq), 162.2 (Cq), 159.9 (Cq), 139.4 (Cq), 139.1 (Cq), 138.4 (Cq), 129.9 (CH), 129.6 (CH), 129.1 (CH), 128.9 (CH), 128.8 (CH), 128.7 (CH), 121.0 (CH), 114.4 (CH), 112.5 (CH), 83.0 (Cq), 55.3 (OCH_3_), 41.9 (CH_2_), 35.5 (CH_2_).

### (*E*)-6-Hydroxy-*N*-(3-(3-methoxyphenyl)allyl)-4-oxo-1,3-diphenyl-2-thioxo-1,2,3,4-tetrahydropyrimidine-5-carboxamide (**5f**)

The title compound was prepared according to general procedure 2 using amine **4f** (45 mg, 0.28 mmol), ester **1** (85 mg, 0.23 mmol) in anhydrous toluene (0.46 mL). Then, the solvent was removed under vacuum, flash chromatographic purification (elution by gradient from 100 to 70/30 cyclohexane/EtOAc) afforded **5f** (42 mg, 38% yield) as an amorphous white solid. UPLC/MS: Rt = 2.33 min (method A). MS (ESI) *m*/*z*:.486.1 [M + H]^+^, C_27_H_24_N_3_O_4_S [M + H]^+^ calculated, 486.5. ^1^H NMR (600 MHz, CDCl_3_): δ 10.11 (dd, *J* = 5.5, 5.5 Hz, 1H, NH), 7.56–7.48 (m, 7H, Ar), 7.33–7.30 (m, 3H, Ar), 7.25 (dd, *J* = 8.0, 8.0 Hz, 1H, Ar), 6.95 (d, *J* = 7.7 Hz, 1H, Ar), 6.89 (dd, *J* = 2.0, 2.0 Hz, 1H, Ar), 6.84 (dd, *J* = 8.0, 2.2 Hz, 1H), 6.56 (d, *J* = 15.8 Hz, 1H), 6.18 (ddd, *J* = 15.8, 6.4, 6.4 Hz, 1H), 4.22 (ddd, *J* = 6.1, 6.1, 1.4 Hz, 2H), 3.83 (s, 3H). ^13^C NMR (150 MHz, CDCl_3_): δ 179.0 (CS), 170.7 (CONH), 167.4 (Cq), 162.2 (Cq), 159.9 (Cq), 139.3 (Cq), 138.4 (Cq), 137.4 (Cq), 134.1 (CH), 129.8 (CH), 129.7 (CH), 129.2 (CH), 128.9 (CH), 128.8 (CH), 128.6 (CH, 2C), 122.8 (CH), 119.3 (CH), 114.0 (CH), 111.9 (CH), 83.1 (Cq), 55.4 (OCH_3_), 42.5 (CH_2_).

### General Procedure 3: TBS Deprotection

A solution 0.5 M of silylated precursor (1 equiv) was treated with TBAF 1 M solution in THF (1.5 equiv). The reaction mixture stirred for 3 h. Then, the mixture was diluted with EtOAc, washed with water, and concentrated under vacuum. The crude material was purified by flash chromatography.

### 6-Hydroxy-*N*-(4-hydroxy-3,5-dimethoxybenzyl)-4-oxo-1,3-diphenyl-2-thioxo-1,2,3,4-tetrahydropyrimidine-5-carboxamide (**6**)

The title compound was prepared according to general procedure 3 using intermediate **5a** (90 mg, 0.14 mmol), TBAF 1 M in THF (220 μL, 0.27 mmol) in anhydrous THF (0.28 mL). The crude was purified by silica gel flash chromatography (elution by gradient from 100 to 60/40 cyclohexane/EtOAc) to yield **6** (42 mg, 54%) as a pale yellow amorphous solid. UPLC/MS: Rt = 2.08 min (method A). MS (ESI) *m*/*z*: 504.2 [M – H]^−^, C_26_H_22_N_3_O_6_S [M – H]^−^ calculated, 504.5. HRMS (AP-ESI) *m*/*z* calcd for C_26_H_24_N_3_O_6_S [M + H]^+^ 506.1386, found 506.1373. ^1^H NMR (400 MHz, DMSO-*d*_6_): δ 10.18 (dd, *J* = 6.2, 6.2 Hz, 1H, NH), 8.37 (s, 1H, OH), 7.48–7.35 (m, 6H, Ph), 7.30–7.23 (m, 4H, Ph), 6.65 (s, 2H, Ar), 4.46 (d, *J* = 6.1 Hz, 2H), 3.73 (s, 6H). ^13^C (100 MHz, DMSO-*d*_6_): δ 178.6 (CS), 169.4 (Cq), 150.0 (Cq), 139.2 (Cq), 135.3 (Cq), 128.9 (CH), 128.1 (CH), 126.7 (CH), 106.1 (CH), 82.8 (Cq), 56.1 (OCH_3_), 43.7 (CH_2_).

### 6-Hydroxy-*N*-(4-hydroxy-3,5-dimethoxyphenethyl)-4-oxo-1,3-diphenyl-2-thioxo-1,2,3,4-tetrahydropyrimidine-5-carboxamide (**7**)

The title compound was prepared according to general procedure 3 using intermediate **5b** (26 mg, 0.04 mmol), TBAF 1 M in THF (60 μL, 0.06 mmol) in anhydrous THF (0.1 mL). The crude was purified by silica gel flash chromatography (elution by gradient from 100 to 60/40 cyclohexane/EtOAc) to yield **7** (11 mg, 53%) as a pale yellow amorphous solid. UPLC/MS: Rt = 2.11 min (method A). MS (ESI) *m*/*z*: 518.1 [M – H]^−^, C_27_H_24_N_3_O_6_S [M – H]^−^ calculated, 518.6. HRMS (AP-ESI) *m*/*z* calcd for C_27_H_26_N_3_O_6_S [M + H]^+^ 520.1542. ^1^H NMR (400 MHz, CDCl_3_): δ 10.07 (dd, *J* = 5.8, 5.8 Hz, 1H, NH), 7.55–7.42 (m, 6H, Ph), 7.29–7.22 (m, 4H, Ph), 6.40 (s, 2H, Ar), 5.41 (s, 1H, OH), 3.8 (s, 6H), 3.65 (ddd, *J* = 6.1, 6.1, 6.1 Hz, 2H), 2.81 (dd, *J* = 6.8 Hz, 6.8 Hz, 2H). ^13^C (150 MHz, CDCl_3_): δ (CS), 179.0 (CS), 170.6 (CONH), 167.3 (Cq), 162.2 (Cq), 147.3 (Cq), 139.4 (Cq), 138.4 (Cq), 133.8 (Cq), 129.7 (CH), 129.6 (CH), 129.2 (CH), 128.9 (CH), 128.8 (CH), 128.6 (CH), 105.5 (CH), 83.0 (Cq), 56.4 (OCH_3_), 42.2 (CH_2_), 35.5 (CH_2_).

### (*E*)-6-Hydroxy-*N*-(3-(4-hydroxy-3,5-dimethoxyphenyl)allyl)-4-oxo-1,3-diphenyl-2-thioxo-1,2,3,4-tetrahydropyrimidine-5-carboxamide (**8**)

The title compound was prepared according to general procedure 3 using intermediate **5c** (40 mg, 0.06 mmol), TBAF 1 M in THF (90 μL, 0.09 mmol) in anhydrous THF (0.12 mL). The crude was purified by silica gel flash chromatography (elution by gradient from 100 to 60/40 cyclohexane/EtOAc) to yield **8** (29 mg, 88%) as pale yellow amorphous solid. UPLC/MS: Rt = 2.11 min (method A). MS (ESI) *m*/*z*: 530.3 [M – H]^−^, C_28_H_24_N_3_O_6_S [M – H]^−^ calculated, 530.6. HRMS (AP-ESI) *m*/*z* calcd for C_28_H_26_N_3_O_6_S [M + H]^+^ 532.1542, found 532.1524. ^1^H NMR (400 MHz, DMSO-*d*_6_): δ 10.01 (dd, *J* = 6.2, 6.2 Hz, 1H, NH), 8.44 (s, 1H, OH), 7.47–7.28 (m, 11H, Ph, OH), 6.68 (m, 2H, Ar), 6.46 (d, *J* = 15.8 Hz, 1H), 6.17 (ddd, *J* = 15.8, 6.2, 6.2 Hz, 1H), 4.13 (dd, *J* = 6.0, 6.0, 1.4 Hz, 2H), 3.75 (s, 6H). ^13^C NMR (150 MHz, DMSO-*d*_6_): δ 178.7 (CS), 169.6 (CONH), 148.1 (Cq), 139.4 (Cq), 135.7 (Cq), 132.7 (Cq), 129.1 (CH), 128.3 (CH), 126.7 (Cq), 121.7 (CH), 104.0 (CH), 83.2 (Cq), 58.0 (OCH_3_), 41.9 (CH_2_).

### 1-[4-[*tert*-Butyl(dimethyl)silyl]oxy-3,5-dimethoxyphenyl]-*N*-methoxymethanimine (**10**)

Sodium acetate (138 mg, 1.69 mmol) and *N*-methylhydroxylamine hydrochloride (141 mg, 1.69 mmol) were added to a solution of compound **9**(50) (250 mg, 0.844 mmol) in MeOH dry (5 mL) under argon. The reaction mixture was stirred at 50 °C for 5 h until completion of reaction. The solvent was removed under vacuum, the residue was suspended in water (5 mL), and the product was extracted with EtOAc (5 × 3 mL). Collected organic layers were dried with Na_2_SO_4_, filtered, and,concentrated under vacuum affording desired product **10** as a mixture of *E*/*Z* isomers (260 mg, 95% yield). The product was used as such without further purification. UPLC/MS: Rt = 2.42 min (method B). MS (ESI) *m*/*z*: 326.2 [M + H]^+^, C_16_H_27_NO_3_Si [M + H]^+^ calculated, 326.2. ^1^H NMR (400 MHz, CDCl_3_) of major isomer: δ 7.96 (s, 1H), 6.78 (s, 2H), 3.95 (s, 3H), 3.82 (s, 6H), 1.00 (s, 9H, *t*Bu TBS), 0.13 (s, 6H, CH_3_ TBS).

### 1-[[(1,1-Dimethylethyl)dimethylsilyl]oxy]-2,6-dimethoxy-4-[(1*E*)-2-nitroethenyl]benzene (**11**)

Nitromethane (2.7 mL, 50.5 mmol) was carefully added to a mixture of aldehyde **9**(50) (300 mg, 1.01 mmol) and ammonium acetate (77.1 mg, 1.01 mmol) in toluene dry (10 mL) under argon. The reaction mixture was stirred for 20 h at reflux under argon. Then the reaction mixture was cooled at room temperature, quenched with water (10 mL), and extracted with EtOAc (2 × 10 mL). Collected organic layers were dried over Na_2_SO_4_, filtered and concentrated under vacuum. Flash chromatographic purification (elution by gradient from 100 to 95/5 cyclohexane/EtOAc) afforded compound (*E*)-**11** (308 mg, 90% yield) as an amorphous yellow solid. UPLC/MS: Rt = 2.41 min (method B). MS (ESI) *m*/*z*: 340.2 [M + H]^+^, C_16_H_26_NO_5_Si [M + H]^+^ calculated, 340.1. ^1^H NMR (400 MHz, CDCl_3_): δ 7.93 (d, *J* = 13.5 Hz, 1H), 7.52 (d. *J* = 13.5 Hz, 1H), 6.73 (s, 2H), 3.84 (s, 6H), 1.01 (s, 9H, *t*Bu TBS), 0.15 (s, 6H, CH_3_ TBS).

### 3-[4-[*tert*-Butyl(dimethyl)silyl]oxy-3,5-dimethoxyphenyl]prop-2-enenitrile (**13**)

To a solution of diethylcyanomethyl phosphonate (180 μL, 1.1 mmol) in THF (8 mL) was added *t*-BuOK (125 mg, 1.1 mmol) at ice–water bath temperature with stirring for 30 min. After that, aldehyde **9**(50) (300 mg, 1.0 mmol) in THF (3 mL) was added dropwise into the above mixture at room temperature and was stirred overnight. The reaction mixture was quenched with water and extracted with EtOAc, washed with brine, dried over anhydrous Na_2_SO_4_, filtered and concentrated under vacuum. Flash chromatographic purification (elution by gradient from 100 to 85/15 cyclohexane/EtOAc) afforded title compound **13** (270 mg, 84% yield) as an *E*/*Z* mixture in ratio 1:0.12. UPLC/MS: Rt = 2.28 min (method B). MS (ESI) *m*/*z*: 320.2 [M + H]^+^, C_17_H_26_NO_3_Si [M + H]^+^ calculated, 320.2. ^1^H NMR (400 MHz, CDCl_3_) for major isomer: δ 7.29 (d, *J* = 16.5 Hz, 1H), 6.63 (s, 2H), 5.71 (d, *J* = 16.5 Hz, 1H), 3.82 (s, 6H), 1.00 (s, 9H, *t*Bu TBS), 0.12 (s, 6H, CH_3_ TBS).

### 3-(3-Methoxyphenyl)prop-2-enenitrile (**14**)

To a solution of diethylcyanomethyl phosphonate (523 μL, 3.2 mmol) in anhydrous THF (20 mL) was added *t*-BuOK (391 mg, 3.2 mmol) at ice–water bath temperature with stirring for 30 min. After that, to this mixture *m*-anisaldehyde **12** (400 mg, 2.94 mmol) in anhydrous THF (8 mL) was added dropwise at room temperature and was stirred overnight. The reaction mixture was quenched with water and extracted with EtOAc, washed with brine, dried over anhydrous Na_2_SO_4_, filtered and concentrated under vacuum. Flash chromatographic purification (elution by gradient from 100 to 85/15 cyclohexane/EtOAc) afforded title compound **14** (412 mg, 81% yield) as an *E*/*Z* mixture in ratio 1:0.18. UPLC/MS: Rt = 1.96 min (method A). MS (ESI) *m*/*z*: 160.0 [M + H]^+^, C_10_H_10_NO [M + H]^+^ calculated, 160.1. ^1^H NMR (400 MHz, CDCl_3_) for major isomer: δ 7.37 (d, *J* = 16.7 Hz, 1H), 7.31 (d, *J* = 7.9 Hz, 1H), 7.04 (d, *J* = 7.7 Hz, 1H), 6.98 (dd, J = 8.2, 2.6 Hz, 1H), 6.95 (m, 1H), 5.87 (d, *J* = 16.6 Hz, 1H), 3.83 (s, 3H).

### Biology. Cell Viability Assay

Human cancer cell lines A549 (lung adenocarcinoma, ATCC CCL-185), DU-145 (androgen-independent prostate cancer, ATCC HTB-81), and HeLa (cervical carcinoma, ATCC CCL-2) were obtained from ATCC. Cells were routinely grown in minimal essential medium containing Eagle’s salts and l-glutamine supplemented with 10% heat-inactivated FBS in a humidified atmosphere of 5% CO_2_ at 37 °C. To assess the antiproliferative activity of the compounds, cells were seeded at a density of 2500 cells/well (HeLa) or 5000 cells/well (A549, DU-145) in 96-well plates, and cell viability was measured using the MTT assay as described previously.^39^ Values are reported as the mean ± SD of two independent experiments.

### Topoisomerase II Activity Assay

The activity of topoIIα was measured using a decatenation assay (Inspiralis) following the manufacturer’s instructions. Compounds were dissolved in DMSO and used at a concentration ranging from 200 to 1 μM. Final DMSO concentration in the assay was ≤1%. Reaction mixtures were incubated for 30 min at 37 °C and terminated with STEB buffer (40% (w/v) sucrose, 100 mM Tris-HCl, pH 8, 1 mM EDTA, 0.5 mg/mL bromophenol blue). Reaction products were resolved by electrophoresis in 1% agarose gels containing SYBR Safe DNA stain (Invitrogen), scanned, and quantified using the ChemiDoc system (BioRad). IC_50_ values were obtained with GraphPad Prism software (version 5.03) using the band intensities of the dose–response gels. Values are reported as the mean ± SD of two independent experiments.

### Topoisomerase II Cleavage Assay

Poison activity of the compounds was evaluated using a cleavage complex assay (Inspiralis) as described previously.^60^ Compounds were tested at a fixed concentration of 200 μM in the presence of 1 U of topoisomerase II and 500 ng of pBR233 plasmid at 37 °C for 6 min. Final DMSO concentration in the assay was 1%. Reaction products were subjected to electrophoresis in a 1% agarose gel, stained with SYBR Safe DNA stain, and DNA bands were visualized and quantified as described above.

### Computational Studies. Structural Model

The crystal structure of the α isoform of human topoII, cocrystallized with etoposide, was downloaded from the RCSB PDB repository, namely, PDB code 5GWK (α). ARN24319 was considered for the docking and classical molecular dynamic (MD) studies. The protein structure was processed with the Protein Preparation Wizard in the Schrödinger 2017 suite.^61^ The ligand’s structure was generated and prepared with Ligprep for molecular docking, using the OPLS2005 force field and charges. All possible protonation and ionization states were generated at a pH of 7.4. Stereoisomers were generated with a limit of 32 stereoisomers per ligand.

### Docking Calculations

The receptor grid for each target was prepared using the OPLS2005 force field. We specified the area surrounding the cocrystallized ligand (i.e., etoposide) as the receptor-binding pocket. The grid center was set to be the centroid of the bound etoposide. The cubic grid had a side length of 20 Å. For the receptor, we included aromatic hydrogen atoms as potential H-bond donors and halogens as potential acceptors. After grid preparation, ligands were first docked into the generated receptor grids using the extra precision (XP) scoring function. Flexible ligand sampling was considered in the docking procedure. All poses were subjected to postdocking minimization. The conformational degrees of freedom of the ligands were extensively explored by allowing nitrogen inversions as well as multiple ring conformations.

### Classical MD Simulations

The most prevalent binding mode obtained from the docking studies was used for MD simulations with GROMACS version 5.1. All bonds were constrained using the P-LINCS algorithm, with an integration time step of 2 fs. The Verlet cutoff scheme was used with a minimum cutoff of 1.2 nm for short-range Lennard-Jones interactions and the real-space contribution to the fourth-ordered Ewald algorithm, which was used to compute long-range electrostatic interactions. Dispersion correction was applied to energy and pressure terms. Periodic boundary conditions were applied in all three dimensions. Each system was equilibrated in two phases, during which restraints were placed on protein and DNA heavy atoms. The first equilibration was done under an *NVT* ensemble for 500 ps using the v-rescale thermostat (τ_T_ = 0.1 ps) to heat the systems until a temperature of 310 K. The *NVT* thermalization was followed by a 500 ps long *NPT* pressurization using the same thermostat and the Parrinello–Rahman barostat (τ_P_ = 2.0 ps and κ = 4.5 × 10^–4^ bar^–1^) to equilibrate the pressure at 1 bar. Production simulations were carried out under an *NPT* ensemble in the absence of any restraints. A 200 ns production run was conducted for the complex. The analysis was carried out using programs within the GROMACS package and Python-based in-house scripts.

## Supporting Information Available

Procedures for *in vitro* metabolic stability, aqueous kinetic solubility, aqueous thermodynamic solubility, plasma protein binding, animal models, pharmacokinetic studies, Figure S2, ^1^H NMR, ^13^C NMR, and ^19^F NMR spectra, and chromatography analysis of key compounds (PDF)
Molecular formula strings and some data (CSV)
Coordinates information for structure representation (PDB)

## Supplementary Material

jm9b01760_si_003.csv

jm9b01760_si_004.pdb

## Abbreviations Used

AcOH: acetic acid
Arg: arginine
ATP: adenosine triphosphate
AUC: area under the curve
*C*_max_: maximum serum concentration
Clp: systemic plasma cleareance
DCM: dichloromethane
DMF: *N*,*N*-dimethylformamide
DMSO: dimethyl sulfoxide
EDTA: ethylenediaminetetraacetic acid
EtOAc: ethyl acetate
*F*: bioavailability
HWE: Horner–Wadsworth–Emmons
MD: molecular dynamics
MeCN: acetonitrile
MeOH: methanol
iv: intravenous
PK: pharmacokinetics
po: per os
SAR: structure–activity relationship
TBAF: tetrabutylammoinum fluoride
TBS: *tert*-butyldimethylsilyl
THF: tetrahydrofuran
topoII: topoisomeraseII

## References

[ref1] Pommier Y. Drugging Topoisomerases: Lessons and Challenges. ACS Chem. Biol. 2013, 8, 82–95. 10.1021/cb300648v. 23259582 PMC3549721

[ref2] Deweese J. E.; Osheroff N. The DNA Cleavage Reaction of Topoisomerase II: Wolf in Sheep’s Clothing. Nucleic Acids Res. 2009, 37, 738–748. 10.1093/nar/gkn937. 19042970 PMC2647315

[ref3] Deweese J. E.; Osheroff M. A.; Osheroff N. DNA Topology and Topoisomerases: Teaching a “Knotty” Subject. Biochem. Mol. Biol. Educ. 2009, 37, 2–10. 10.1002/bmb.20244. PMC2643378 19225573

[ref4] Iacopetta D.; Rosano C.; Puoci F.; Parisi O. I.; Saturnino C.; Caruso A.; Longo P.; Ceramella J.; Malzert-Freon A.; Dallemagne P.; Rault S.; Sinicropi M. S. Multifaceted Properties of 1,4-Dimethylcarbazoles: Focus on Trimethoxybenzamide and Trimethoxyphenylurea Derivatives as Novel Human Topoisomerase II Inhibitors. Eur. J. Pharm. Sci. 2017, 96, 263–272. 10.1016/j.ejps.2016.09.039. 27702608

[ref5] Rosenblum D.; Joshi N.; Tao W.; Karp J. M.; Peer D. Progress and Challenges Towards Targeted Delivery of Cancer Therapeutics. Nat. Commun. 2018, 9, 1410 10.1038/s41467-018-03705-y. 29650952 PMC5897557

[ref6] Hu W.; Huang X. S.; Wu J. F.; Yang L.; Zheng Y. T.; Shen Y. M.; Li Z. Y.; Li X. Discovery of Novel Topoisomerase II Inhibitors by Medicinal Chemistry Approaches. J. Med. Chem. 2018, 61, 8947–8980. 10.1021/acs.jmedchem.7b01202. 29870668

[ref7] Pommier Y.; Sun Y.; Huang S. N.; Nitiss J. L. Roles of Eukaryotic Topoisomerases in Transcription, Replication and Genomic Stability. Nat. Rev. Mol. Cell Biol. 2016, 17, 703–721. 10.1038/nrm.2016.111. 27649880 PMC9248348

[ref8] Nitiss J. L. DNA Topoisomerase II and Its Growing Repertoire of Biological Functions. Nat. Rev. Cancer 2009, 9, 327–337. 10.1038/nrc2608. 19377505 PMC2730144

[ref9] Lindsey R. H. jr.; Pendleton M.; Ashley R. E.; Mercer S. L.; Deweese J. E.; Osheroff N. Catalytic Core of Human Topoisomerase IIalpha: Insights into Enzyme-DNA Interactions and Drug Mechanism. Biochemistry 2014, 53, 6595–6602. 10.1021/bi5010816. 25280269 PMC4204876

[ref10] Liang X.; Wu Q.; Luan S.; Yin Z.; He C.; Yin L.; Zou Y.; Yuan Z.; Li L.; Song X.; He M.; Lv C.; Zhang W. A Comprehensive Review of Topoisomerase Inhibitors as Anticancer Agents in the Past Decade. Eur. J. Med. Chem. 2019, 171, 129–168. 10.1016/j.ejmech.2019.03.034. 30917303

[ref11] Wang W.; Tse-Dinh Y. C. Recent Advances in Use of Topoisomerase Inhibitors in Combination Cancer Therapy. Curr. Top. Med. Chem. 2019, 19, 730–740. 10.2174/1568026619666190401113350. 30931861

[ref12] Hande K. R. Topoisomerase II Inhibitors. Cancer Chemother. Biol. Response Modif. 2003, 21, 103–125. 10.1016/S0921-4410(03)21005-X. 15338742

[ref13] Baldwin E. L.; Osheroff N. Etoposide, Topoisomerase II and Cancer. Curr. Med. Chem.: Anti-Cancer Agents 2005, 5, 363–372. 10.2174/1568011054222364. 16101488

[ref14] Palermo G.; Minniti E.; Greco M. L.; Riccardi L.; Simoni E.; Convertino M.; Marchetti C.; Rosini M.; Sissi C.; Minarini A.; De Vivo M. An Optimized Polyamine Moiety Boosts the Potency of Human Type II Topoisomerase Poisons as Quantified by Comparative Analysis Centered on the Clinical Candidate F14512. Chem. Commun. (Cambridge, U. K.) 2015, 51, 14310–14313. 10.1039/C5CC05065K. 26234198

[ref15] Baviskar A. T.; Amrutkar S. M.; Trivedi N.; Chaudhary V.; Nayak A.; Guchhait S. K.; Banerjee U. C.; Bharatam P. V.; Kundu C. N. Switch in Site of Inhibition: A Strategy for Structure-Based Discovery of Human Topoisomerase IIalpha Catalytic Inhibitors. ACS Med. Chem. Lett. 2015, 6, 481–485. 10.1021/acsmedchemlett.5b00040. 25941559 PMC4416441

[ref16] Kadayat T. M.; Park S.; Shrestha A.; Jo H.; Hwang S. Y.; Katila P.; Shrestha R.; Nepal M. R.; Noh K.; Kim S. K.; Koh W. S.; Kim K. S.; Jeon Y. H.; Jeong T. C.; Kwon Y.; Lee E. S. Discovery and Biological Evaluations of Halogenated 2,4-Diphenyl Indeno[1,2-B]Pyridinol Derivatives as Potent Topoisomerase IIalpha-Targeted Chemotherapeutic Agents for Breast Cancer. J. Med. Chem. 2019, 62, 8194–8234. 10.1021/acs.jmedchem.9b00970. 31398033

[ref17] Gouveia R. G.; Ribeiro A. G.; Segundo M.; de Oliveira J. F.; de Lima M.; de Lima Souza T. R. C.; de Almeida S. M. V.; de Moura R. O. Synthesis, DNA and Protein Interactions and Human Topoisomerase Inhibition of Novel Spiroacridine Derivatives. Bioorg. Med. Chem. 2018, 26, 5911–5921. 10.1016/j.bmc.2018.10.038. 30420325

[ref18] Pogorelcnik B.; Perdih A.; Solmajer T. Recent Developments of DNA Poisons--Human DNA Topoisomerase IIalpha Inhibitors--as Anticancer Agents. Curr. Pharm. Des. 2013, 19, 2474–2488. 10.2174/1381612811319130016. 23363399

[ref19] Pogorelcnik B.; Perdih A.; Solmajer T. Recent Advances in the Development of Catalytic Inhibitors of Human DNA Topoisomerase IIalpha as Novel Anticancer Agents. Curr. Med. Chem. 2013, 20, 694–709. 10.2174/092986713804999402. 23210851

[ref20] Riddell I. A.; Agama K.; Park G. Y.; Pommier Y.; Lippard S. J. Phenanthriplatin Acts as a Covalent Poison of Topoisomerase II Cleavage Complexes. ACS Chem. Biol. 2016, 11, 2996–3001. 10.1021/acschembio.6b00565. 27648475 PMC5248983

[ref21] Delgado J. L.; Hsieh C. M.; Chan N. L.; Hiasa H. Topoisomerases as Anticancer Targets. Biochem. J. 2018, 475, 373–398. 10.1042/BCJ20160583. 29363591 PMC6110615

[ref22] Froelich-Ammon S. J.; Osheroff N. Topoisomerase Poisons: Harnessing the Dark Side of Enzyme Mechanism. J. Biol. Chem. 1995, 270, 21429–21432. 10.1074/jbc.270.37.21429. 7665550

[ref23] Gibson E. G.; King M. M.; Mercer S. L.; Deweese J. E. Two-Mechanism Model for the Interaction of Etoposide Quinone with Topoisomerase IIalpha. Chem. Res. Toxicol. 2016, 29, 1541–1548. 10.1021/acs.chemrestox.6b00209. 27533850

[ref24] Bailly C. Contemporary Challenges in the Design of Topoisomerase II Inhibitors for Cancer Chemotherapy. Chem. Rev. 2012, 112, 3611–3640. 10.1021/cr200325f. 22397403

[ref25] Wu C. C.; Li T. K.; Farh L.; Lin L. Y.; Lin T. S.; Yu Y. J.; Yen T. J.; Chiang C. W.; Chan N. L. Structural Basis of Type II Topoisomerase Inhibition by the Anticancer Drug Etoposide. Science 2011, 333, 459–462. 10.1126/science.1204117. 21778401

[ref26] Wendorff T. J.; Schmidt B. H.; Heslop P.; Austin C. A.; Berger J. M. The Structure of DNA-Bound Human Topoisomerase II Alpha: Conformational Mechanisms for Coordinating Inter-Subunit Interactions with DNA Cleavage. J. Mol. Biol. 2012, 424, 109–124. 10.1016/j.jmb.2012.07.014. 22841979 PMC3584591

[ref27] Cowell I. G.; Sondka Z.; Smith K.; Lee K. C.; Manville C. M.; Sidorczuk-Lesthuruge M.; Rance H. A.; Padget K.; Jackson G. H.; Adachi N.; Austin C. A. Model for MLL Translocations in Therapy-Related Leukemia Involving Topoisomerase IIbeta-Mediated DNA Strand Breaks and Gene Proximity. Proc. Natl. Acad. Sci. U. S. A. 2012, 109, 8989–8994. 10.1073/pnas.1204406109. 22615413 PMC3384169

[ref28] Pendleton M.; Lindsey R. H. jr.; Felix C. A.; Grimwade D.; Osheroff N. Topoisomerase II and Leukemia. Ann. N. Y. Acad. Sci. 2014, 1310, 98–110. 10.1111/nyas.12358. 24495080 PMC3961513

[ref29] Azarova A. M.; Lyu Y. L.; Lin C. P.; Tsai Y. C.; Lau J. Y.; Wang J. C.; Liu L. F. Roles of DNA Topoisomerase II Isozymes in Chemotherapy and Secondary Malignancies. Proc. Natl. Acad. Sci. U. S. A. 2007, 104, 11014–11019. 10.1073/pnas.0704002104. 17578914 PMC1904155

[ref30] Cowell I. G.; Austin C. A. Mechanism of Generation of Therapy Related Leukemia in Response to Anti-Topoisomerase II Agents. Int. J. Environ. Res. Public Health 2012, 9, 2075–2091. 10.3390/ijerph9062075. 22829791 PMC3397365

[ref31] Pommier Y.; Leo E.; Zhang H.; Marchand C. DNA Topoisomerases and Their Poisoning by Anticancer and Antibacterial Drugs. Chem. Biol. 2010, 17, 421–433. 10.1016/j.chembiol.2010.04.012. 20534341 PMC7316379

[ref32] Hu C. X.; Zuo Z. L.; Xiong B.; Ma J. G.; Geng M. Y.; Lin L. P.; Jiang H. L.; Ding J. Salvicine Functions as Novel Topoisomerase II Poison by Binding to Atp Pocket. Mol. Pharmacol. 2006, 70, 1593–1601. 10.1124/mol.106.027714. 16914642

[ref33] Walker J. V.; Nitiss J. L. DNA Topoisomerase II as a Target for Cancer Chemotherapy. Cancer Invest. 2002, 20, 570–589. 10.1081/CNV-120002156. 12094551

[ref34] Dimaggio J. J.; Warrell R. P. Jr.; Muindi J.; Stevens Y. W.; Lee S. J.; Lowenthal D. A.; Haines I.; Walsh T. D.; Baltzer L.; Yaldaei S.; Young C. W. Phase I Clinical and Pharmacological Study of Merbarone. Cancer Res. 1990, 50, 1151–1155. 2297763

[ref35] Fortune J. M.; Osheroff N. Merbarone Inhibits the Catalytic Activity of Human Topoisomerase IIalpha by Blocking DNA Cleavage. J. Biol. Chem. 1998, 273, 17643–17650. 10.1074/jbc.273.28.17643. 9651360

[ref36] Malik U. R.; Dutcher J. P.; Caliendo G.; Lasala P.; Mitnick R.; Wiernik P. H. Phase II Trial of Merbarone in Patients with Malignant Brain Tumors. Med. Oncol. 1997, 14, 159–162. 10.1007/BF02989644. 9468039

[ref37] Spallarossa A.; Rotolo C.; Sissi C.; Marson G.; Greco M. L.; Ranise A.; La Colla P.; Busonera B.; Loddo R. Further Sar Studies on Bicyclic Basic Merbarone Analogues as Potent Antiproliferative Agents. Bioorg. Med. Chem. 2013, 21, 6328–6336. 10.1016/j.bmc.2013.08.056. 24063907

[ref38] Larsen A. K.; Escargueil A. E.; Skladanowski A. Catalytic Topoisomerase II Inhibitors in Cancer Therapy. Pharmacol. Ther. 2003, 99, 167–181. 10.1016/S0163-7258(03)00058-5. 12888111

[ref39] Ortega J. A.; Riccardi L.; Minniti E.; Borgogno M.; Arencibia J. M.; Greco M. L.; Minarini A.; Sissi C.; De Vivo M. Pharmacophore Hybridization to Discover Novel Topoisomerase II Poisons with Promising Antiproliferative Activity. J. Med. Chem. 2018, 61, 1375–1379. 10.1021/acs.jmedchem.7b01388. 29077404

[ref40] Ranise A.; Spallarossa A.; Schenone S.; Bruno O.; Bondavalli F.; Pani A.; Marongiu M. E.; Mascia V.; La Colla P.; Loddo R. Synthesis and Antiproliferative Activity of Basic Thioanalogues of Merbarone. Bioorg. Med. Chem. 2003, 11, 2575–2589. 10.1016/S0968-0896(03)00158-5. 12757725

[ref41] Minniti E.; Byl J. A. W.; Riccardi L.; Sissi C.; Rosini M.; De Vivo M.; Minarini A.; Osheroff N. Novel Xanthone-Polyamine Conjugates as Catalytic Inhibitors of Human Topoisomerase IIalpha. Bioorg. Med. Chem. Lett. 2017, 27, 4687–4693. 10.1016/j.bmcl.2017.09.011. 28919339 PMC5623067

[ref42] Oviatt A. A.; Kuriappan J. A.; Minniti E.; Vann K. R.; Onuorah P.; Minarini A.; De Vivo M.; Osheroff N. Polyamine-Containing Etoposide Derivatives as Poisons of Human Type II Topoisomerases: Differential Effects on Topoisomerase IIalpha and IIbeta. Bioorg. Med. Chem. Lett. 2018, 28, 2961–2968. 10.1016/j.bmcl.2018.07.010. 30006062 PMC6097886

[ref43] Riccardi L.; Genna V.; De Vivo M. Metal–Ligand Interactions in Drug Design. Nat. Rev. Chem. 2018, 2, 100–112. 10.1038/s41570-018-0018-6.

[ref44] Meanwell N. A. Synopsis of Some Recent Tactical Application of Bioisosteres in Drug Design. J. Med. Chem. 2011, 54, 2529–2591. 10.1021/jm1013693. 21413808

[ref45] Tanaka R; Hirayama N. Structure of Etoposide. Anal. Sci.: X-Ray Struct. Anal. Online 2007, 23, x29–x30. 10.2116/analscix.23.x29.

[ref46] Wilstermann A. M.; Bender R. P.; Godfrey M.; Choi S.; Anklin C.; Berkowitz D. B.; Osheroff N.; Graves D. E. Topoisomerase II - Drug Interaction Domains: Identification of Substituents on Etoposide That Interact with the Enzyme. Biochemistry 2007, 46, 8217–8225. 10.1021/bi700272u. 17580961 PMC2888091

[ref47] Bender R. P.; Jablonksy M. J.; Shadid M.; Romaine I.; Dunlap N.; Anklin C.; Graves D. E.; Osheroff N. Substituents on Etoposide That Interact with Human Topoisomerase IIalpha in the Binary Enzyme-Drug Complex: Contributions to Etoposide Binding and Activity. Biochemistry 2008, 47, 4501–4509. 10.1021/bi702019z. 18355043 PMC2737519

[ref48] Ding S.; Jiao N. N,N-Dimethylformamide: A Multipurpose Building Block. Angew. Chem., Int. Ed. 2012, 51, 9226–9237. 10.1002/anie.201200859. 22930476

[ref49] Yang D.-S.; Jeon H.-B. Convenient N-Formylation of Amines in Dimethylformamide with Methyl Benzoate under Microwave Irradiation. Bull. Korean Chem. Soc. 2010, 31, 1424–1426. 10.5012/bkcs.2010.31.5.1424.

[ref50] Pettit G. R.; Searcy J. D.; Tan R.; Cragg G. M.; Melody N.; Knight J. C.; Chapuis J. C. Antineoplastic Agents. 585. Isolation of Bridelia Ferruginea Anticancer Podophyllotoxins and Synthesis of 4-Aza-Podophyllotoxin Structural Modifications. J. Nat. Prod. 2016, 79, 507–518. 10.1021/acs.jnatprod.5b00873. 26938998

[ref51] Inspiralis Limited. Https://www.Inspiralis.Com/Assets/Technicaldocuments/Human-Topo-II-Alpha-Decatenation-Assay-Protocol.Pdf (accessed Oct 9, 2019).

[ref52] De Vivo M.; Cavalli A. Recent Advances in Dynamic Docking for Drug Discovery. Wiley Interdiscip. Rev.: Comput. Mol. Sci. 2017, 7, e1320 10.1002/wcms.1320.

[ref53] Franco-Ulloa S.; La Sala G.; Miscione G. P.; De Vivo M. Novel Bacterial Topoisomerase Inhibitors Exploit Asp83 and the Intrinsic Flexibility of the DNA Gyrase Binding Site. Int. J. Mol. Sci. 2018, 19, 453 10.3390/ijms19020453. PMC5855675 29401640

[ref54] Kuriappan J. A.; Osheroff N.; De Vivo M. Smoothed Potential MD Simulations for Dissociation Kinetics of Etoposide to Unravel Isoform Specificity in Targeting Human Topoisomerase II. J. Chem. Inf. Model. 2019, 59, 4007–4017. 10.1021/acs.jcim.9b00605. 31449404 PMC6800198

[ref55] De Vivo M.; Masetti M.; Bottegoni G.; Cavalli A. Role of Molecular Dynamics and Related Methods in Drug Discovery. J. Med. Chem. 2016, 59, 4035–4061. 10.1021/acs.jmedchem.5b01684. 26807648

[ref56] Wang Y. R.; Chen S. F.; Wu C. C.; Liao Y. W.; Lin T. S.; Liu K. T.; Chen Y. S.; Li T. K.; Chien T. C.; Chan N. L. Producing Irreversible Topoisomerase II-Mediated DNA Breaks by Site-Specific Pt(II)-Methionine Coordination Chemistry. Nucleic Acids Res. 2017, 45, 10861–10871. 10.1093/nar/gkx742. 28977631 PMC5737487

[ref57] Palermo G.; Stenta M.; Cavalli A.; Dal Peraro M.; De Vivo M. Molecular Simulations Highlight the Role of Metals in Catalysis and Inhibition of Type II Topoisomerase. J. Chem. Theory Comput. 2013, 9, 857–862. 10.1021/ct300691u. 26588728

[ref58] Palermo G.; Cavalli A.; Klein M. L.; Alfonso-Prieto M.; Dal Peraro M.; De Vivo M. Catalytic Metal Ions and Enzymatic Processing of DNA and RNA. Acc. Chem. Res. 2015, 48, 220–228. 10.1021/ar500314j. 25590654

[ref59] Jacob D. A.; Mercer S. L.; Osheroff N.; Deweese J. E. Etoposide Quinone Is a Redox-Dependent Topoisomerase II Poison. Biochemistry 2011, 50, 5660–5667. 10.1021/bi200438m. 21595477 PMC3119725

[ref60] Bandele O. J.; Osheroff N. Cleavage of Plasmid DNA by Eukaryotic Topoisomerase II. Methods Mol. Biol. 2009, 582, 39–47. 10.1007/978-1-60761-340-4_4. 19763940 PMC2893727

[ref61] Sastry G. M.; Adzhigirey M.; Day T.; Annabhimoju R.; Sherman W. Protein and Ligand Preparation: Parameters, Protocols, and Influence on Virtual Screening Enrichments. J. Comput.-Aided Mol. Des. 2013, 27, 221–234. 10.1007/s10822-013-9644-8. 23579614

