# Supplementary material for: Design, Synthesis, Dynamic Docking, Biochemical Characterization, and in Vivo Pharmacokinetics Studies of Novel Topoisomerase II Poisons with Promising Antiproliferative Activity
Source: J Med Chem. 2020 Mar 20;63(7):3508–21. doi: 10.1021/acs.jmedchem.9b01760 (PMC7997578; doi:10.1021/acs.jmedchem.9b01760)
Supplement: Supplementary file 1 — jm9b01760_si_001.pdf [file jm9b01760_si_001.pdf]

SUPPORTING INFORMATION

Design, synthesis, dynamic docking, biochemical characterization, and *in vivo* pharmacokinetics studies of novel topoisomerase II poisons with promising antiproliferative activity

Jose M. Arencibia,#[1] Nicoletta Brindani,#[1] Sebastian Franco-Ulloa,[1] Michela Nigro,[1] Jissy Akkarapattiakal Kuriappan,[1] Giuliana Ottonello,[2] Sine Mandrup Bertozzi,[2] Maria Summa,[2] Stefania Girotto,[1] Rosalia Bertorelli,[2] Andrea Armirotti,[2] Marco De Vivo*,[1]

1. Molecular Modeling and Drug Discovery Lab, Istituto Italiano di Tecnologia, via Morego 30, 16163 Genova, Italy

2. Analytical Chemistry and in vivo Pharmacology, Istituto Italiano di Tecnologia, via Morego 30, 16163 Genova, Italy

Table of contents

- Title page and table of contents S1
- 1. *In vitro* metabolic stability S2
- 2. Aqueous kinetic solubility S2
- 3. Aqueous thermodynamic solubility S3
- 4. Plasma protein binding S3
- 4. Animal models S4
- 4. Pharmacokinetic studies S4
- 5. Figure S2 S5
- 6. ^{1}H NMR, ^{13}C NMR and ^{19}F NMR spectra S6
- 7. Chromatography analysis of key compounds S44

1. *In vitro* metabolic stability

In vitro microsomial stability. 10mM DMSO stock solution of test compound was pre-incubated at 37˚C for 15min with *mouse* liver microsomes added 0.1M Tris-HCl buffer (pH 7.4). The final concentration was 4.6µM. After pre-incubation, the co-factors (NADPH, G6P, G6PDH and $MgCl_2$ pre-dissolved in 0.1M Tris-HCl) were added to the incubation mixture and the incubation was continued at 37˚C for 1h. At each time point (0, 5, 15, 30, 60min), 30µL of incubation mixture was diluted with 200µL cold CH_3CN spiked with 200nM of internal standard, followed by centrifugation at 3500g for 15min. The supernatant was further diluted with H_2O (1:1) for analysis. The concentration of test compound was quantified by LC/MS-MS on a Waters ACQUITY UPLC/MS TQD system consisting of a TQD (Triple Quadrupole Detector) Mass Spectrometer equipped with an Electrospray Ionization interface. The analyses were run on an ACQUITY UPLC BEH C18 (50x2.1mmID, particle size 1.7µm) with a VanGuard BEH C18 pre-column (5x2.1mmID, particle size 1.7µm) at 40°C, using 0.1% HCOOH in H_2O (A) and 0.1% HCOOH in CH_3CN (B) as mobile phase. Electrospray ionization (ESI) was applied in positive mode. The percentage of test compound remaining at each time point relative to t=0 was calculated. The half-lives ($t_{½}$) were determined by an one-phase decay equation using a non-linear regression of compound concentration versus time.

In vitro Plasma Stability**.** 10mM DMSO stock solution of test compound was diluted 50-fold with DMSO-H_2O (1:1) and incubated at 37˚C for 2 h with *mouse* plasma added 5% DMSO (pre-heated at 37˚C for 10 min). The final concentration was 2µM. At each time point (0, 5, 15, 30, 60, 120min), 50 µL of incubation mixture was diluted with 200µL cold CH_3CN spiked with 200nM of internal standard, followed by centrifugation at 3500g for 20min. The supernatant was further diluted with H_2O (1:1) for analysis. The concentration of test compound was quantified by LC/MS-MS on a Waters ACQUITY UPLC/MS TQD system consisting of a TQD (Triple Quadrupole Detector) Mass Spectrometer equipped with an Electrospray Ionization interface. The analyses were run on an ACQUITY UPLC BEH C18 (50x2.1mmID, particle size 1.7µm) with a VanGuard BEH C18 pre-column (5x2.1mmID, particle size 1.7µm) at 40°C, using 0.1% HCOOH in H_2O (A) and 0.1% HCOOH in CH_3CN (B) as mobile phase. Electrospray ionization (ESI) was applied in positive mode. The response factors, calculated on the basis of the internal standard peak area, were plotted over time. When possible, response vs. time profiles were fitted with Prism (GraphPad Software, Inc., USA) to estimate compounds half-life in plasma.

2. Aqueous kinetic solubility

The aqueous kinetic solubility was determined from a 10mM DMSO stock solution of test compound in Phosphate Buffered Saline (PBS) at pH 7.4. The study was performed by incubation of an aliquot of 10mM DMSO stock solution in PBS (pH 7.4) at a target concentration of 250µM resulting in a final concentration of 2.5% DMSO. The incubation was carried out under shaking at 25°C for 24h followed by centrifugation at 21.100g for 30min. The supernatant was analyzed by UPLC/MS for the quantification of dissolved compound by UV at a specific wavelength (215nm).
The analyses were performed on a Waters ACQUITY UPLC/MS SQD system consisting of a SQD (Single Quadrupole Detector) Mass Spectrometer equipped with Electrospray Ionization interface.
The analyses were run on an ACQUITY UPLC BEH C_{18} column (50x2.1mmID, particle size 1.7µm) with a VanGuard BEH C_{18} pre-column (5x2.1mmID, particle size 1.7µm), using 10mM NH_4OAc in H_2O at pH 5 adjusted with AcOH (A) and 10mM NH_4OAc in MeCN-H_2O (95:5) at pH 5 (B) as mobile phase.

3. Aqueous thermodynamic solubility

The thermodynamic solubility of **3f** was determined by addition of Phosphate Buffered Saline (PBS) at pH 7.4 to an excess of solid compound.
The assay was performed by incubation of an aliquot of 2.5mg of test compound in 500µL of PBS at pH 7.4. The suspension was shaken at 300 RPM for 24h at 25°C. At the end of the incubation period, the saturated solution was filtered and analyzed by UPLC/MS for the quantification of dissolved compound (in µM) by UV at a specific wavelength (λmax of the test compound). The analyses were performed on a Waters ACQUITY UPLC/MS SQD system equipped with an ESI ion source. The analyses were run on an ACQUITY UPLC BEH C_{18} column (50x2.1mmID, particle size 1.7µm) with a VanGuard BEH C_{18} pre-column (5x2.1mmID, particle size 1.7µm), using H_2O + 0.1% HCOOH (A) and CH_3CN + 0.1% HCOOH (B) as mobile phase.

4. Plasma Protein Binding

The assay was conducted in triplicate in human and mouse plasma. An appropriate volume of plasma was spiked with a known amount of 10mM DMSO stock solution of the test compound, thus reaching the incubation concentrations of 0.5, 1 and 5µM. Then 300µL/well of the spiked plasma were added to the corresponding donor well of the RED plate (Rapid Equilibrium Dialysis (RED) Device Single-Use Plate with Inserts, Thermo Scientific), a polypropylene plate preloaded with 48 equilibrium dialysis membrane inserts (8K MWCO). 500µL/well of isotonic buffer (PBS, pH 7.4) were added to the receiver well of the RED plate. The RED plate was sealed with adhesive film and shaken at 500 RPM for 3 hours at 37°C. At the end of the incubation period, the "Donor Spin" plate was prepared by adding 100µL of isotonic buffer, 10µL of blank plasma and 10µL from each donor chamber of the RED device, while the "Receiver Spin" plate was prepared by adding 20µL of blank plasma and 100µL from each receiver chamber of the RED device. The "Standards Spin" plate was prepared by adding 100µL of isotonic buffer, 10µL of spiked plasma and 10µL of blank plasma. 300µL of stop solution (Acetonitrile spiked with 200nM of the appropriate internal standard) were added to each well of the three "spin" plates, that subsequently were centrifuged for 20 minutes at 3750 RPM. At this point, the supernatants were ready for injections in the UPLC-MS/MS system. The analyses were performed on a Waters ACQUITY UPLC/MS TQD system consisting of a TQD (triple quadrupole detector) Mass Spectrometer equipped with an Electrospray Ionization interface and a Photodiode Array eλ Detector. The analyses were run on an ACQUITY UPLC BEH C_{18} (50x2.1mmID, particle size 1.7µm) with a VanGuard BEH C_{18} pre-column (5x2.1mmID, particle size 1.7µm) at 40°C, using H_2O + 0.1% HCOOH (A) and CH_3CN + 0.1% HCOOH (B) as mobile phase. Electrospray ionization (ESI) was applied in positive mode and MRM transitions and collision energies were chosen for the specific test compound. Plasma protein binding was calculated applying appropriate corrections to compensate for dilutions during sampling.

3. Animal models

Male C57B6/J male mice, 8 weeks old, 25-30 g were used (Charles River). All procedures were performed in accordance with the Ethical Guidelines of European Communities Council (Directive 2010/63/EU of 22 September 2010) and accepted by the Italian Ministry of Health. All efforts were made to minimize animal suffering and to use the minimal number of animals required to produce reliable results, according to the "3Rs concept". Animals were group-housed in ventilated cages (n=5 mice per cage) and had free access to food and water. They were maintained under a 12-hour light/dark cycle (lights on at 8:00 am) at controlled temperature (21°C ± 1°C) and relative humidity (55% ± 10%).

4. Pharmacokinetic studies

Compound **3f** was administered orally (P.O.) and intravenously (I.V.) to C57B6/J male mice at 10 and 3 mg/Kg dose. Vehicle was: PEG400/Tween 80/Saline solution at 10/10/80 % in volume respectively. 3f was first dissolved in 100% PEG, then diluted to the final target concentration in this vehicle. The target concentration of 3f in the dosing solution was checked and confirmed by LC-MS. The dosing solutions were diluted differently in the vehicle. The different dilutions were further diluted 4-fold with CH_3CN prior to analysis. A reference standard was spiked in the vehicle to prepare a calibration curve over a 0.1-10μM range. The calibrators were also diluted 4-fold with CH3CN as for the dosing solution dilutions. The dosing solutions and calibrators were analysed under the same conditions as for the plasma samples described below.

Three animals per dose were treated. Blood samples at 0, 15, 30, 60, 120, 240, and 480 minutes after administration were collected for PO arm. Blood samples at 0, 5, 15, 30, 60, 120 and 240 minutes after administration were collected for IV arm. Plasma was separated from blood by centrifugation for 15 minutes at 3500 rpm a 4°C, collected in a eppendorf tube and frozen (-80°C). Brain samples were homogenized in Phosphate buffered saline and were then split in two aliquots kept at -80 °C until analysis. An aliquot was used for compound brain level evaluations, following the same procedure described below for plasma samples. The second aliquot was kept for protein content evaluation by bicinchoninic acid assay (BCA). Control animals treated with vehicle only were also included in the experimental protocol.

Plasma samples were centrifuged at 21.100g for 15min. at 4°C. A 50μl aliquot was transferred into a 96-Deep Well plate and 150μl of extraction solution was added. The extraction solution was consisting of cold CH_3CN spiked with 200nM of internal standard. The plate was centrifuged at 3270g for 15min. at 4°C. 80μl of supernatant was then transferred into a 96-Deep Well plate and 80μl of H_2O was added. A reference standard of the compound was spiked in naïve mouse plasma to prepare a calibration curve over a 1nM – 10μM range. Three quality control samples were prepared by spiking the compound in blank mouse plasma to the final concentrations of 20, 200 and 2000nM. Calibrators and quality controls were extracted with the same extraction solution used for the plasma samples. The samples were analysed on a Waters ACQUITY UPLC/MS TQD system (Waters Inc. Milford, USA) consisting of a TQD (Triple Quadrupole Detector) Mass Spectrometer equipped with an Electrospray Ionization interface and a Photodiode Array eλ Detector. The analyses were run on an ACQUITY UPLC BEH C_{18} (50x2.1mmID, particle size 1.7μm) with a VanGuard BEH C_{18} pre-column (5x2.1mmID, particle size 1.7μm) at 40°C. H_2O + 0.1% HCOOH (A) and CH_3CN + 0.1% HCOOH (B) were used as mobile phase with a linear gradient from 50 to 100%B in 2min. with the flow rate set to 0.5mL/min. Electrospray ionization was applied in positive mode. Plasma levels of the parent compound was quantified by monitoring the MRM peak areas.

5. Figure S2

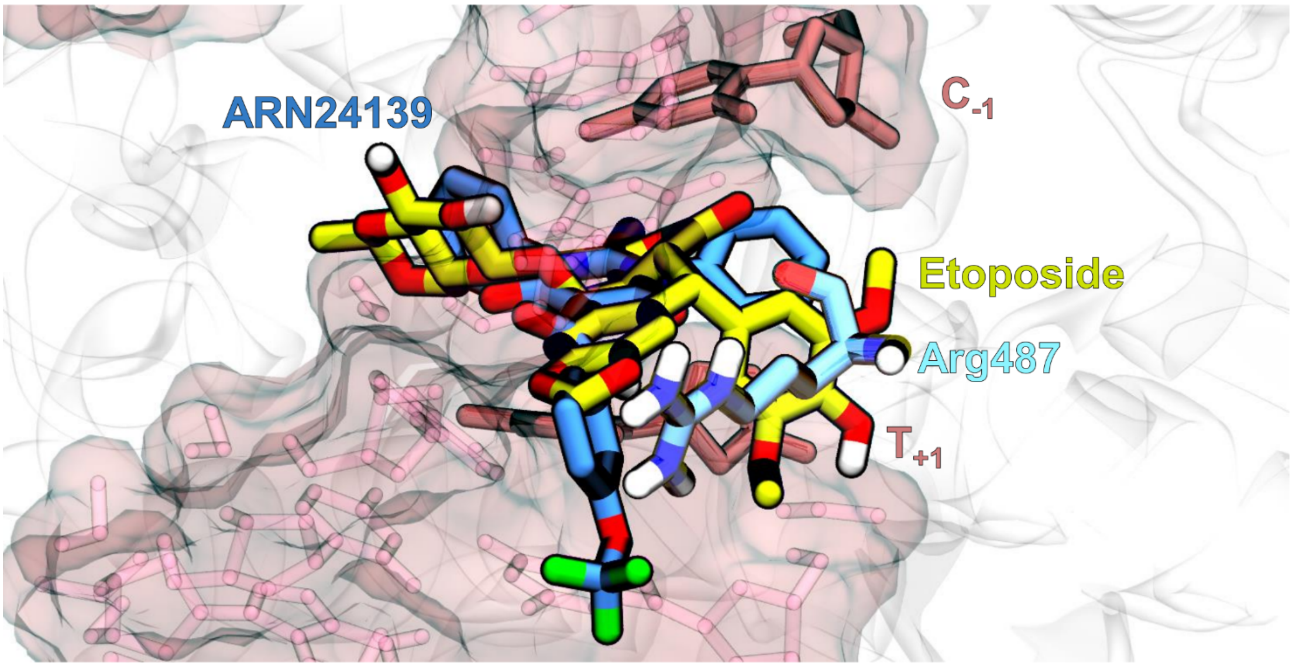

Figure S2. Binding mode superposition of etoposide (yellow carbons, PDBID: 5GWK) and compound ARN24139 (blue carbons) into topoIIα. Molecular docking calculations reveal a favourable π-cation interaction between the E-ring and Arg487.

6. ^{1}H, ^{13}C, ^{19}F NMR Spectra

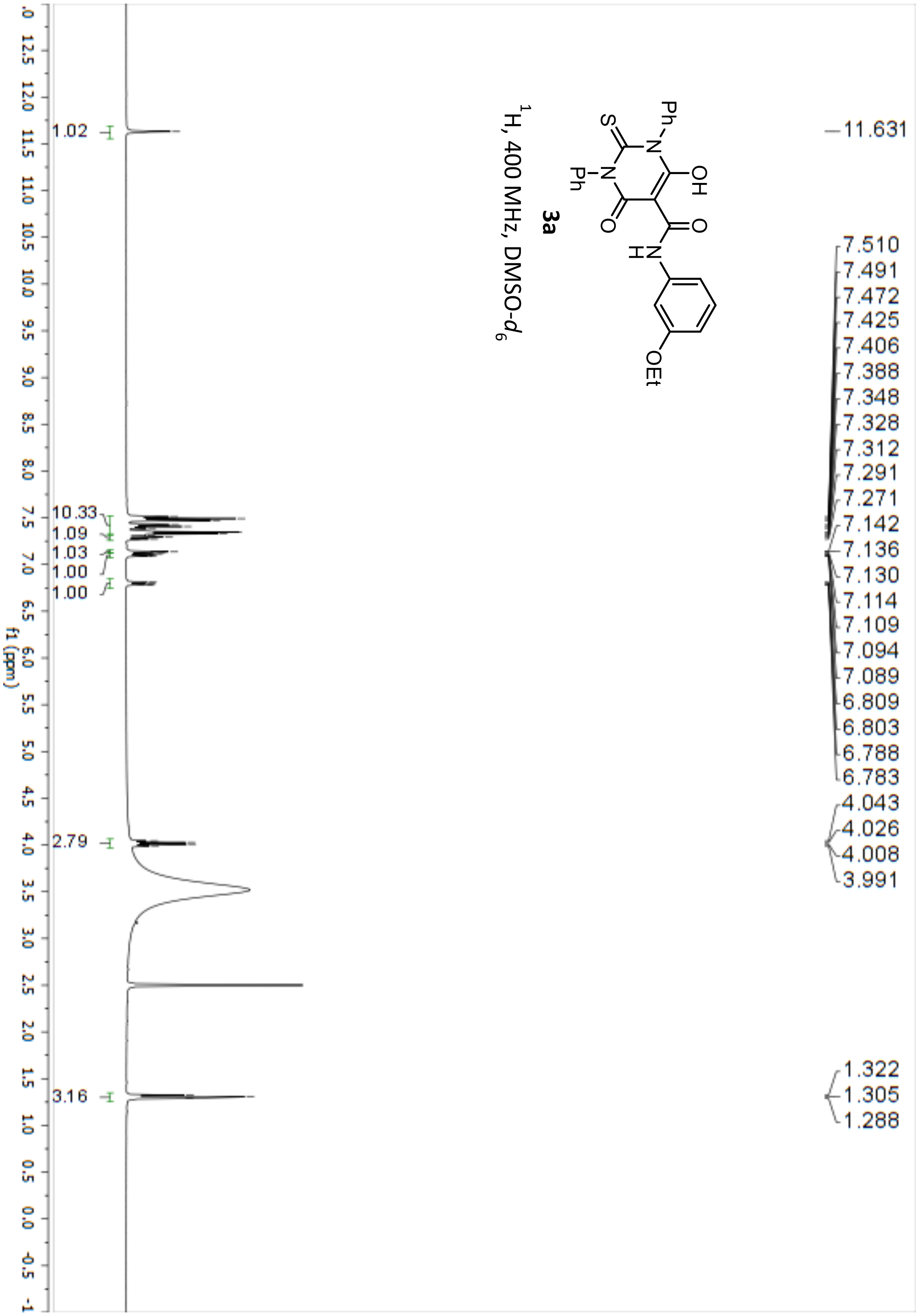

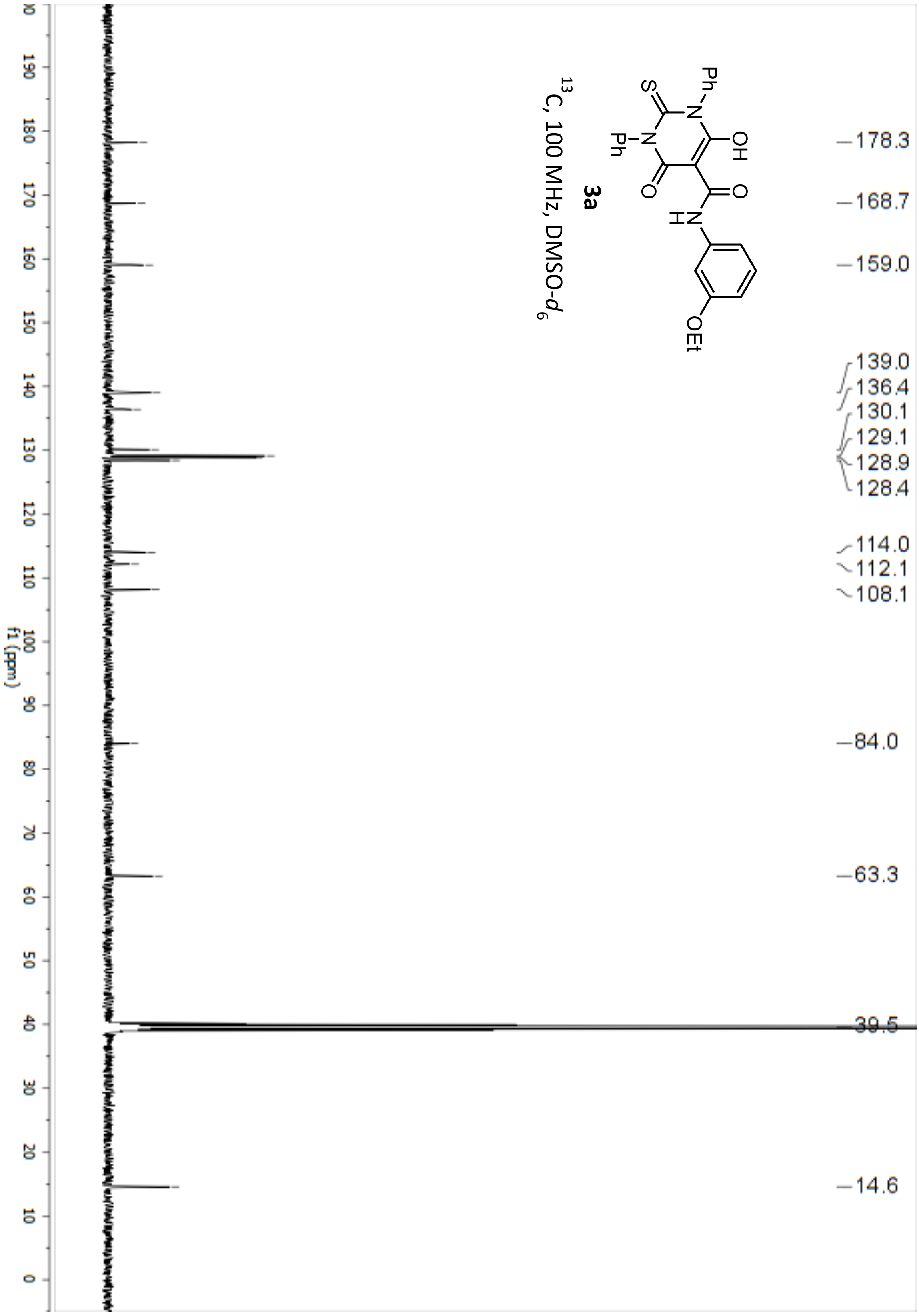

3a
^{13}C, 100 MHz, DMSO-d_6
178.3
168.7
159.0
139.0
136.4
130.1
129.1
128.9
128.4
114.0
112.1
108.1
84.0
63.3
39.5
14.6
f1 (ppm)

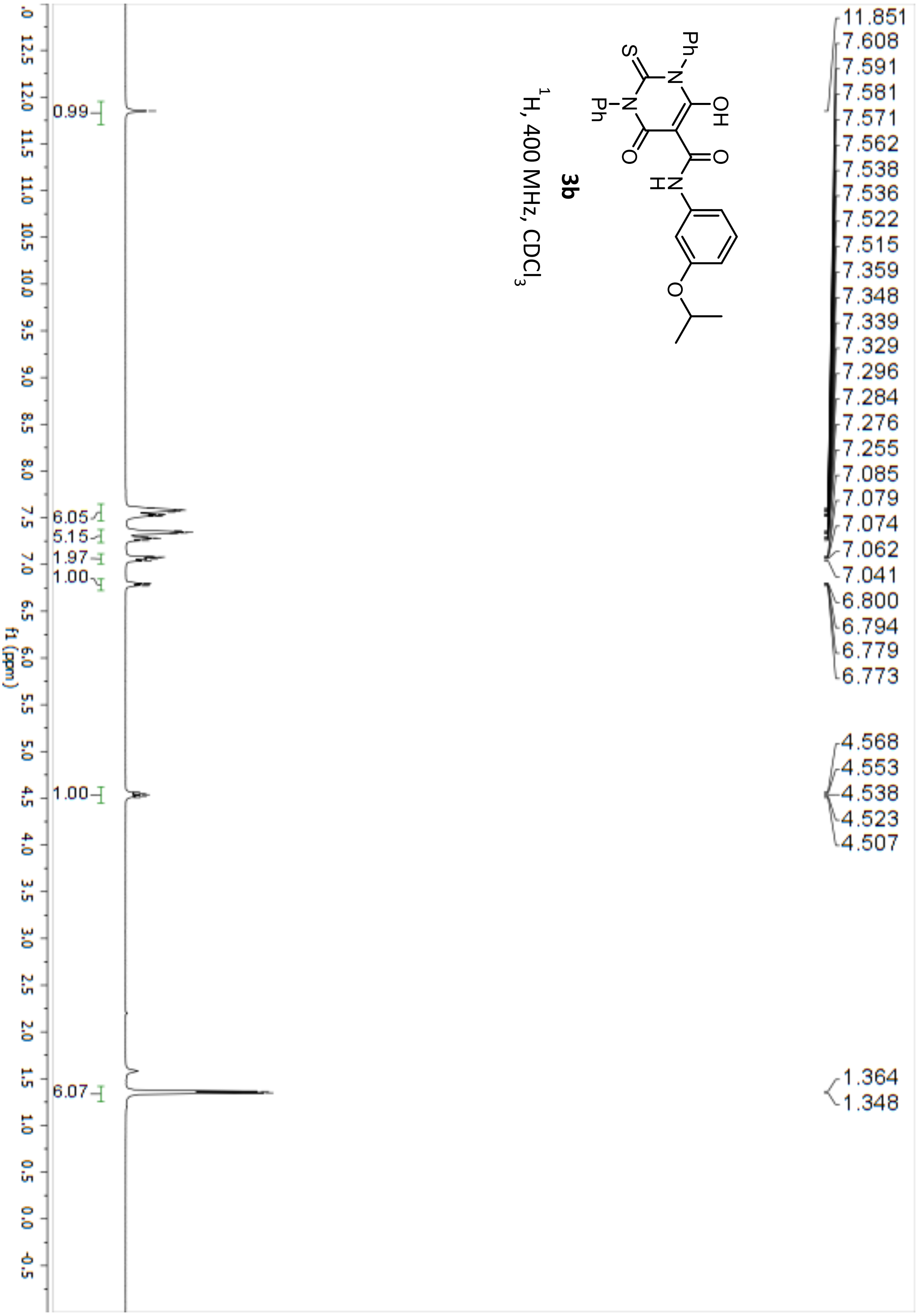
11.851
7.608
7.591
7.581
7.571
7.562
7.538
7.536
7.522
7.515
7.359
7.348
7.339
7.329
7.296
7.284
7.276
7.255
7.085
7.079
7.074
7.062
7.041
6.800
6.794
6.779
6.773
4.568
4.553
4.538
4.523
4.507
1.364
1.348
OH
O
N
H
Ph
N
S
N
Ph
O
3b
1H, 400 MHz, CDCl3
0.99
6.05
5.15
1.97
1.00
1.00
6.07
f1 (ppm)

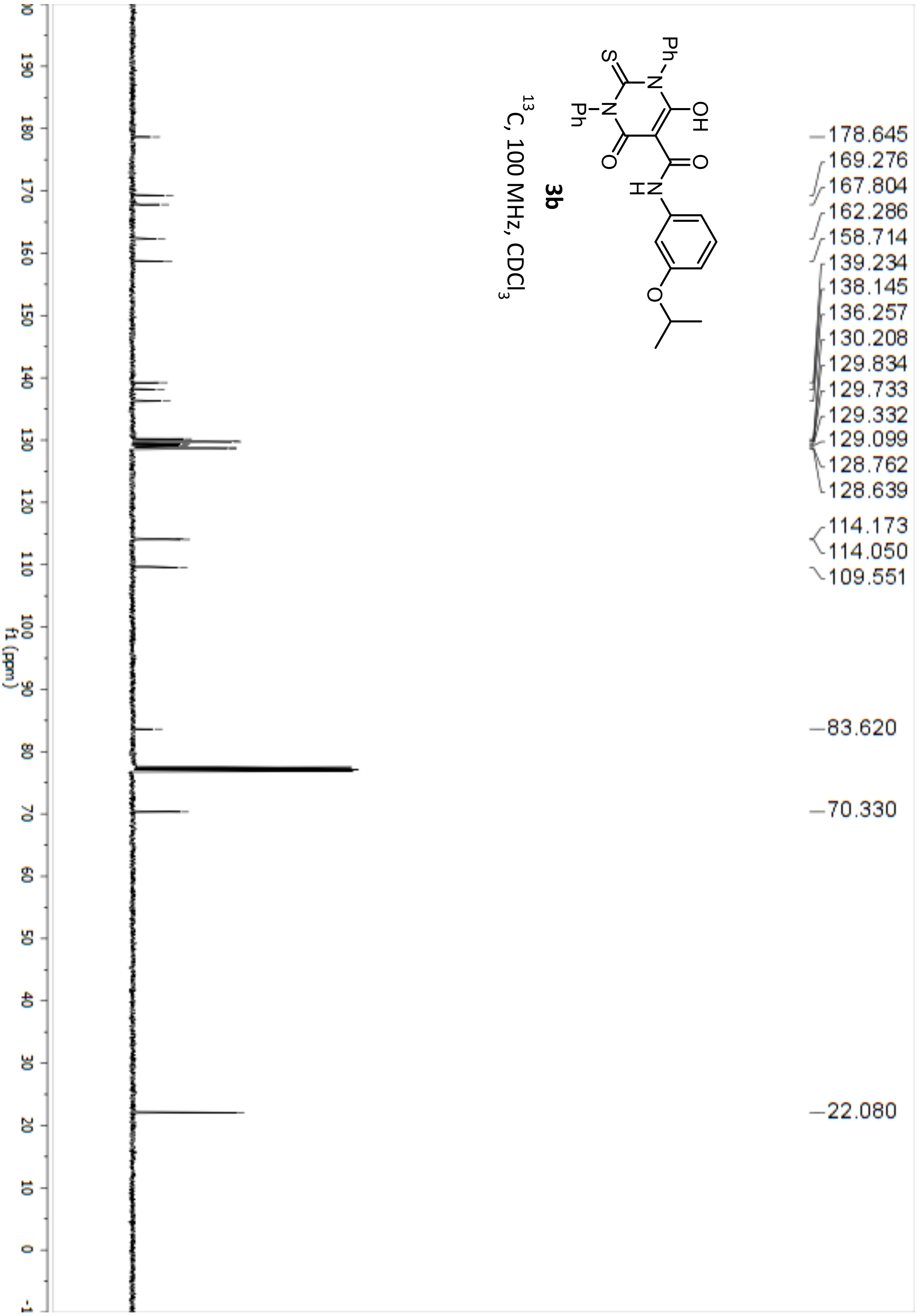
178.645
169.276
167.804
162.286
158.714
139.234
138.145
136.257
130.208
129.834
129.733
129.332
129.099
128.762
128.639
114.173
114.050
109.551
83.620
70.330
22.080
f1 (ppm)
3b
^{13}C, 100 MHz, CDCl$_3$

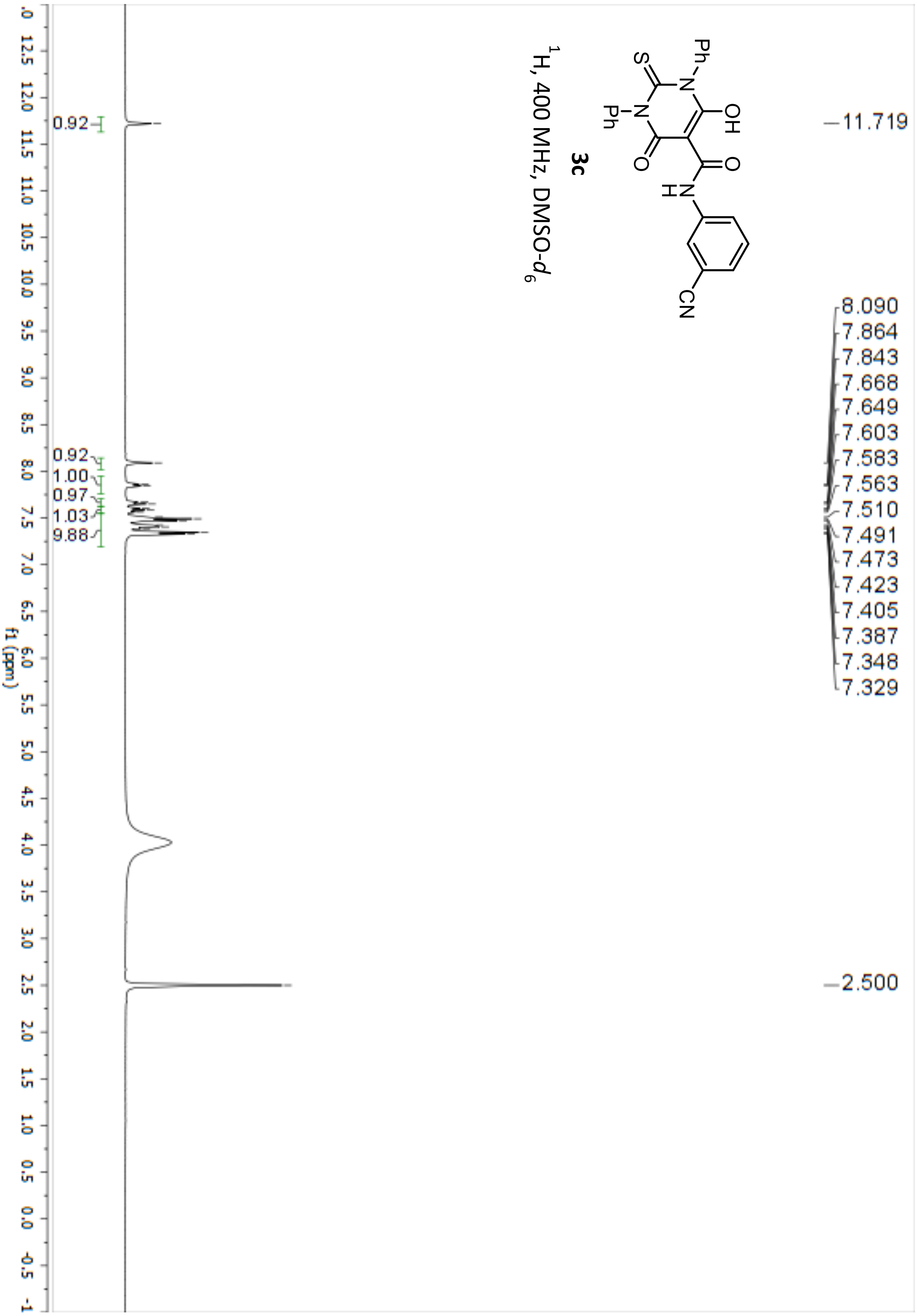
3c
1H, 400 MHz, DMSO-d6
11.719
8.090
7.864
7.843
7.668
7.649
7.603
7.583
7.563
7.510
7.491
7.473
7.423
7.405
7.387
7.348
7.329
2.500
0.92
0.92
1.00
0.97
1.03
9.88
f1 (ppm)

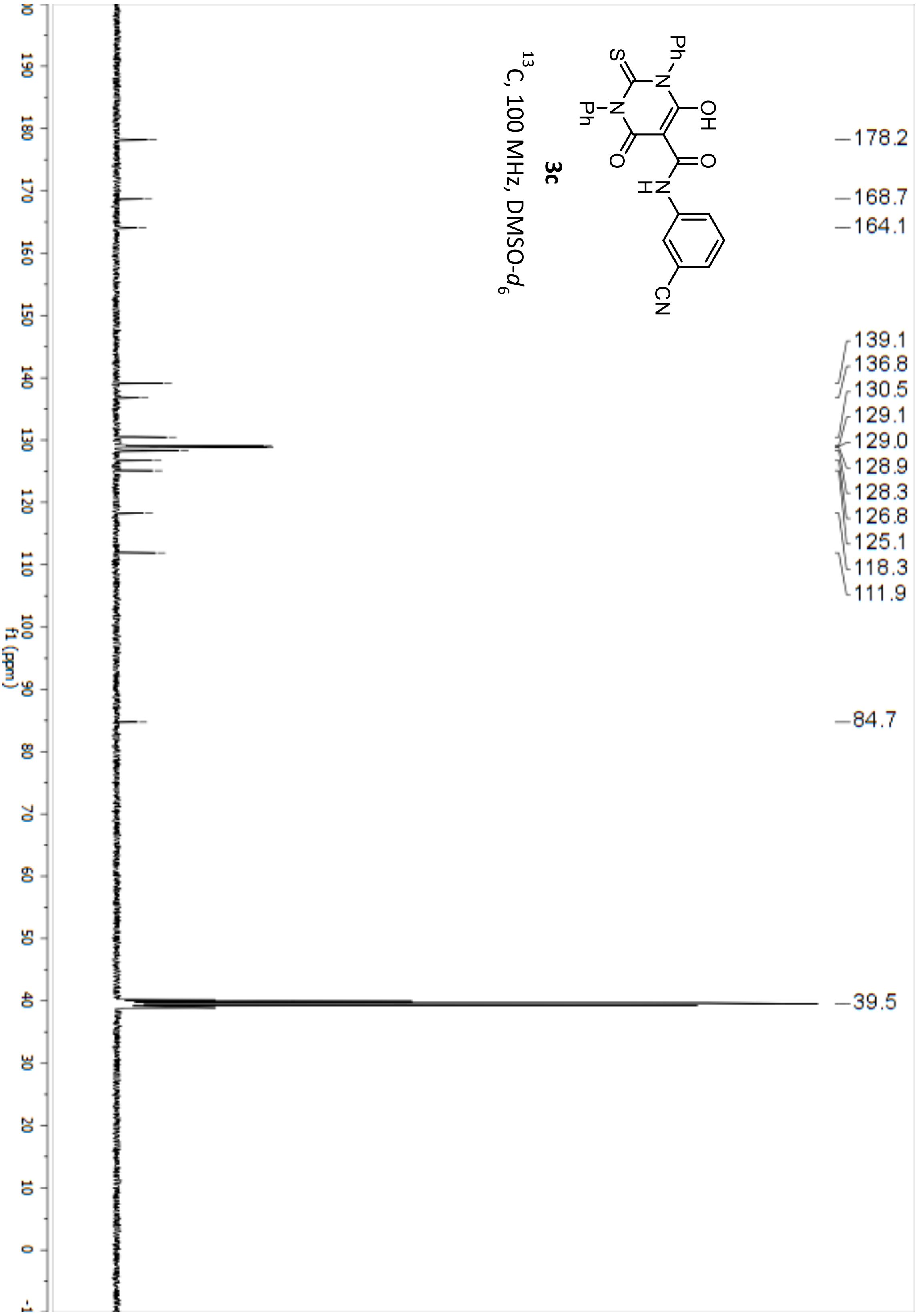
3c
13C, 100 MHz, DMSO-d6
178.2
168.7
164.1
139.1
136.8
130.5
129.1
129.0
128.9
128.3
126.8
125.1
118.3
111.9
84.7
39.5
f1 (ppm)

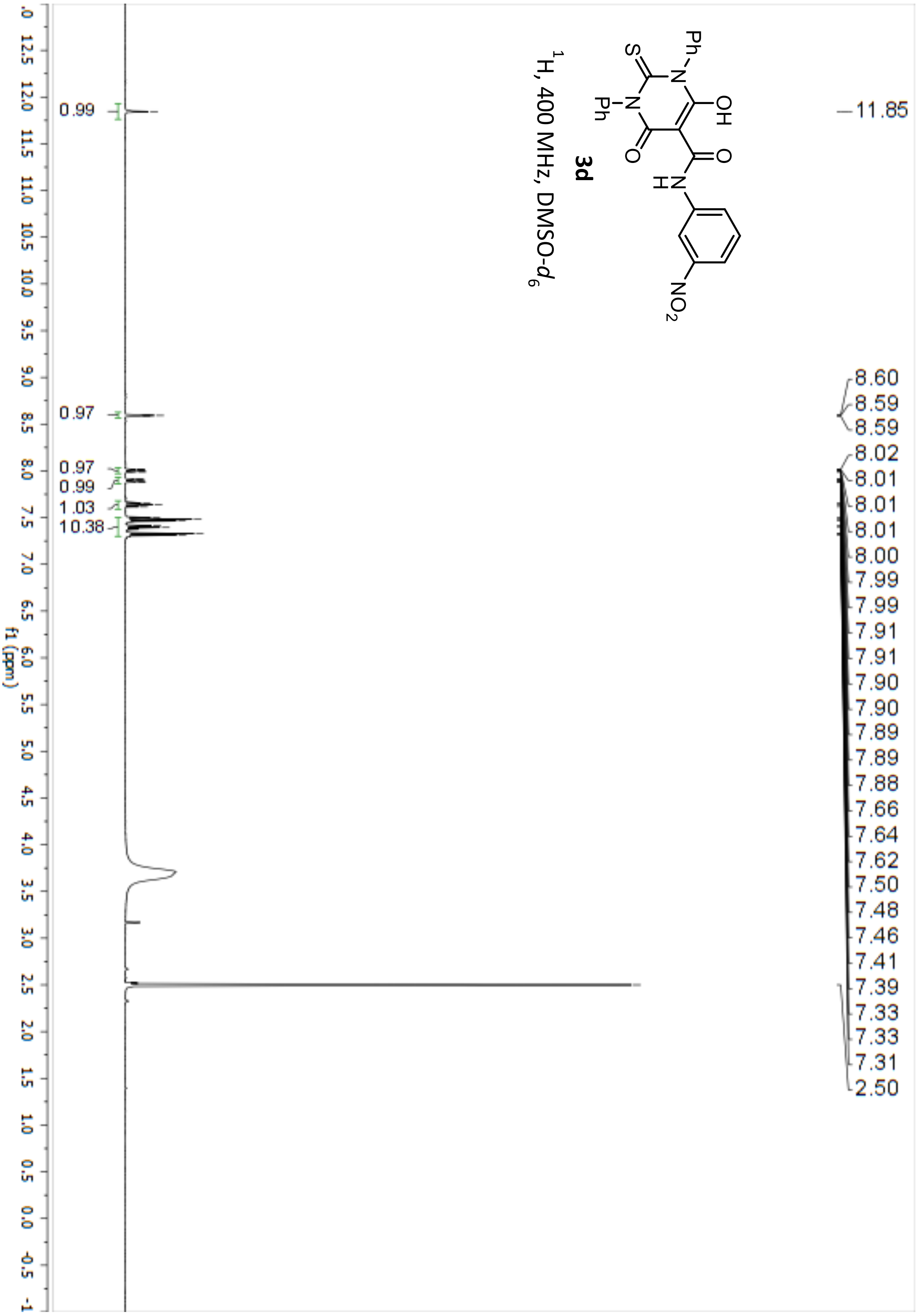
3d
1H, 400 MHz, DMSO-d6
OH
O
N
H
NO2
Ph
S
O
11.85
8.60
8.59
8.59
8.02
8.01
8.01
8.01
8.00
7.99
7.99
7.91
7.91
7.90
7.90
7.89
7.89
7.88
7.66
7.64
7.62
7.50
7.48
7.46
7.41
7.39
7.33
7.33
7.31
2.50
0.99
0.97
0.97
0.99
1.03
10.38
f1 (ppm)

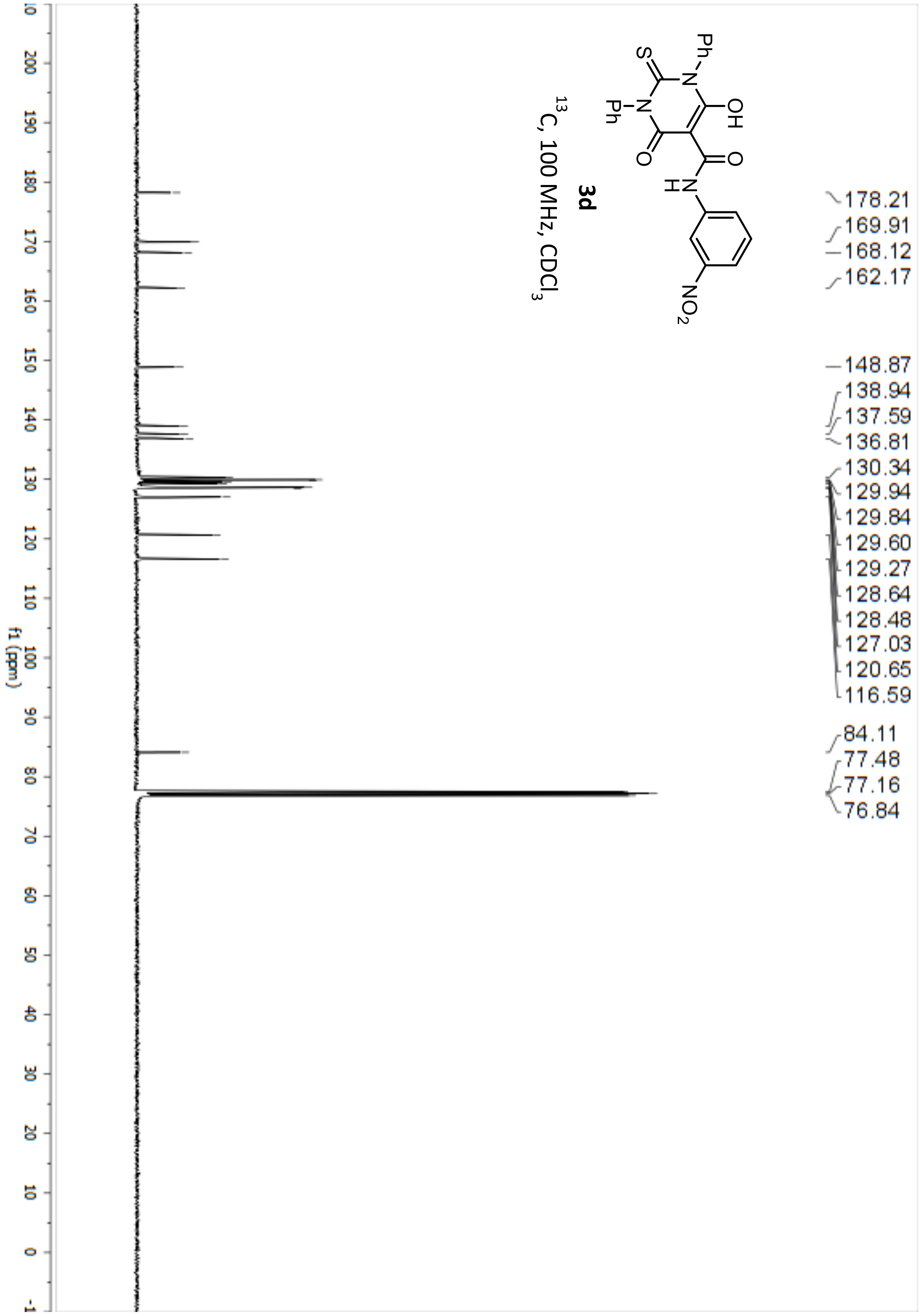
3d
^{13}C, 100 MHz, $CDCl_3$
178.21
169.91
168.12
162.17
148.87
138.94
137.59
136.81
130.34
129.94
129.84
129.60
129.27
128.64
128.48
127.03
120.65
116.59
84.11
77.48
77.16
76.84
f1 (ppm)

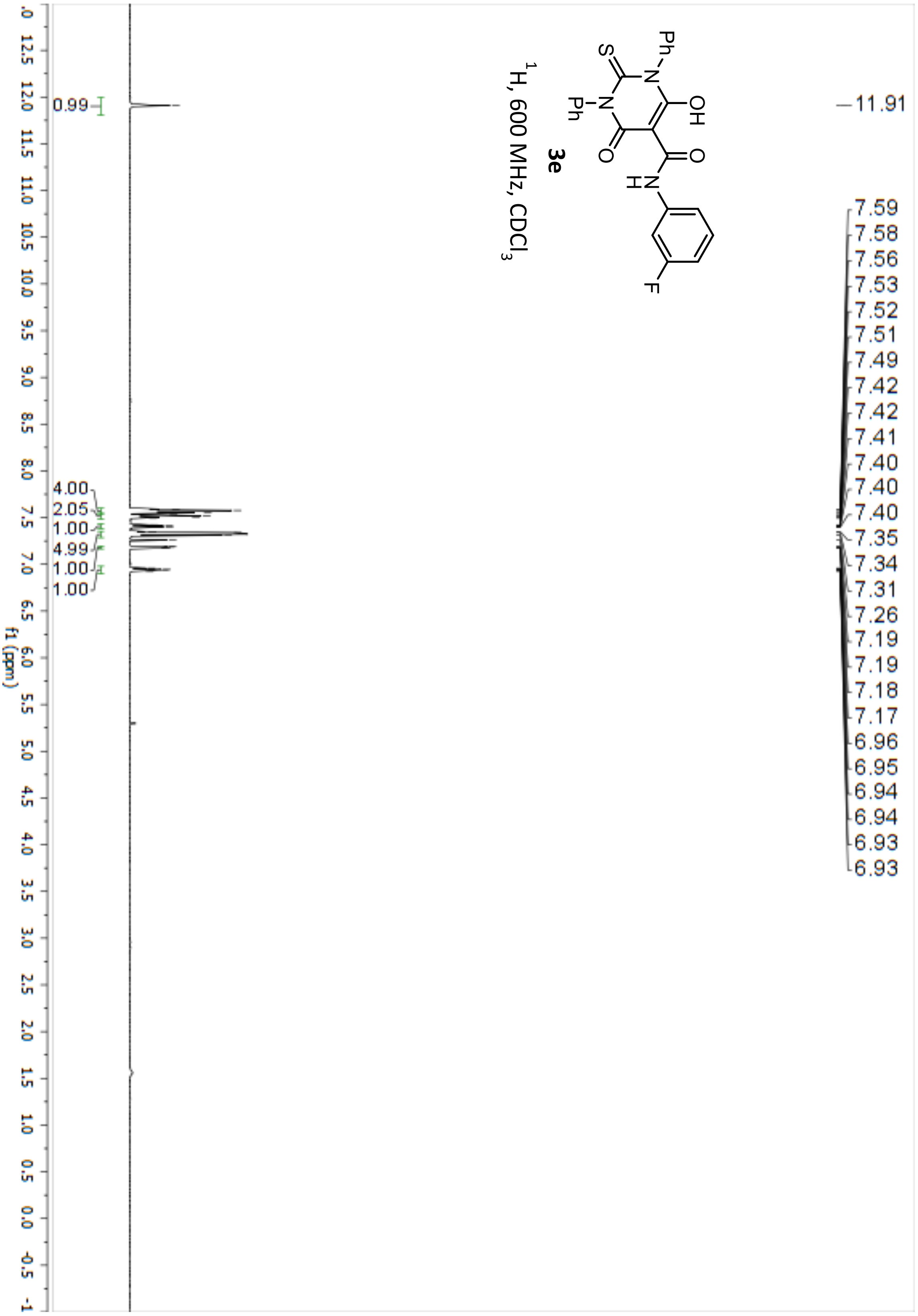
3e
1H, 600 MHz, CDCl3
OH
Ph
N
S
O
H
F
11.91
7.59
7.58
7.56
7.53
7.52
7.51
7.49
7.42
7.42
7.41
7.40
7.40
7.40
7.35
7.34
7.31
7.26
7.19
7.19
7.18
7.17
6.96
6.95
6.94
6.94
6.93
6.93
0.99
4.00
2.05
1.00
4.99
1.00
1.00
f1 (ppm)

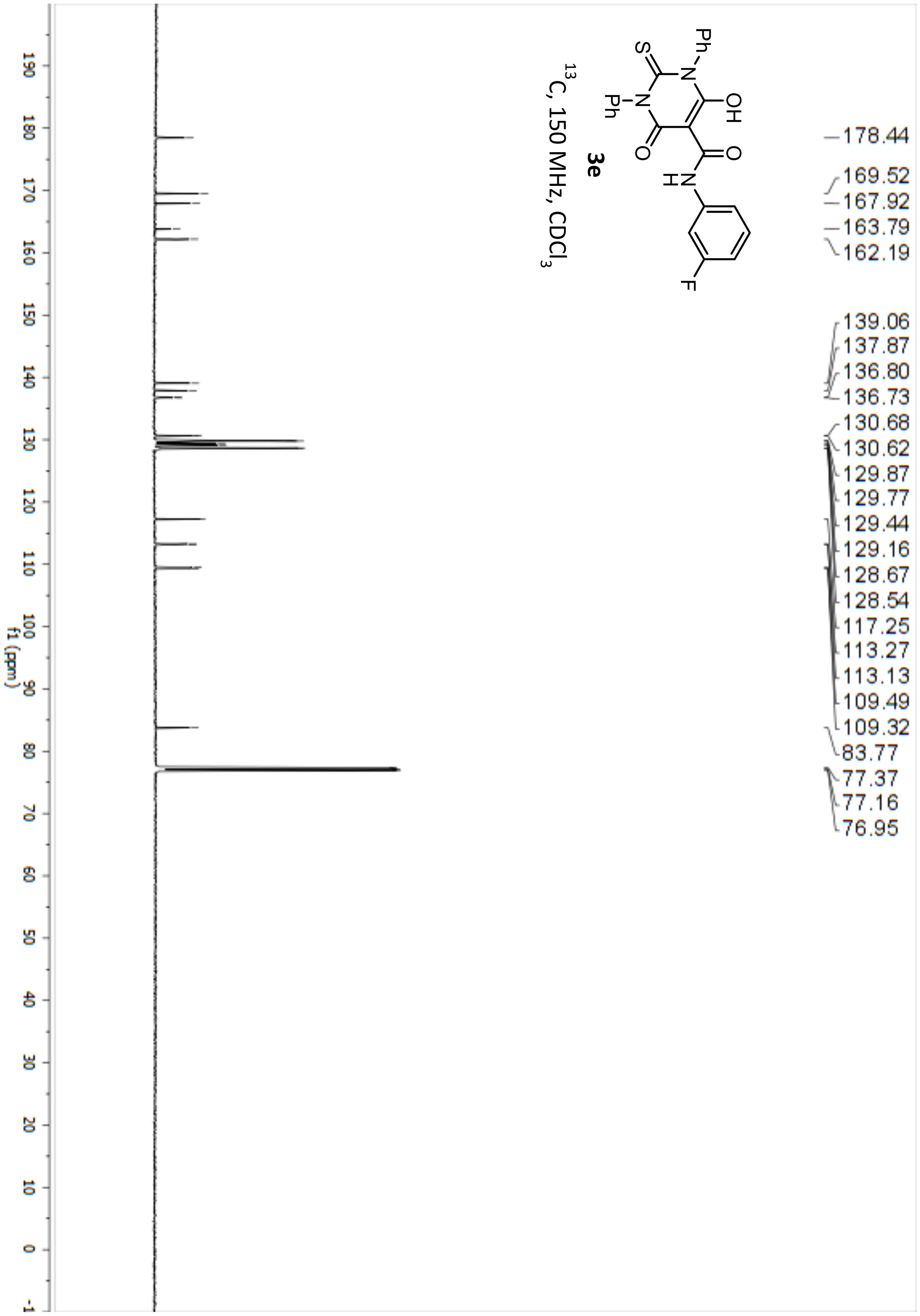
3e
13C, 150 MHz, CDCl3
178.44
169.52
167.92
163.79
162.19
139.06
137.87
136.80
136.73
130.68
130.62
129.87
129.77
129.44
129.16
128.67
128.54
117.25
113.27
113.13
109.49
109.32
83.77
77.37
77.16
76.95
f1 (ppm)

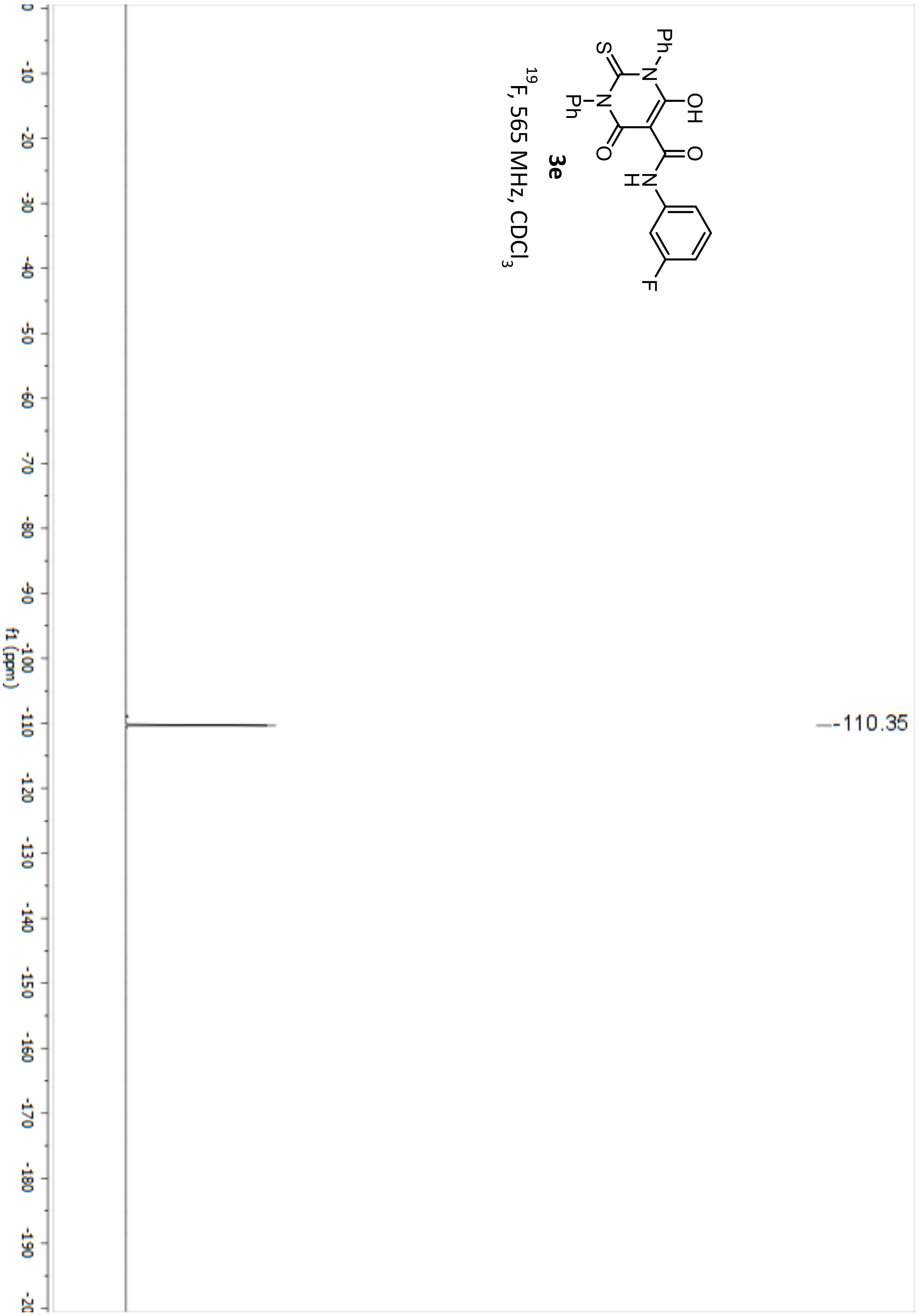
3e
19F, 565 MHz, CDCl3
-110.35
f1 (ppm)
0
-10
-20
-30
-40
-50
-60
-70
-80
-90
-100
-110
-120
-130
-140
-150
-160
-170
-180
-190
-200

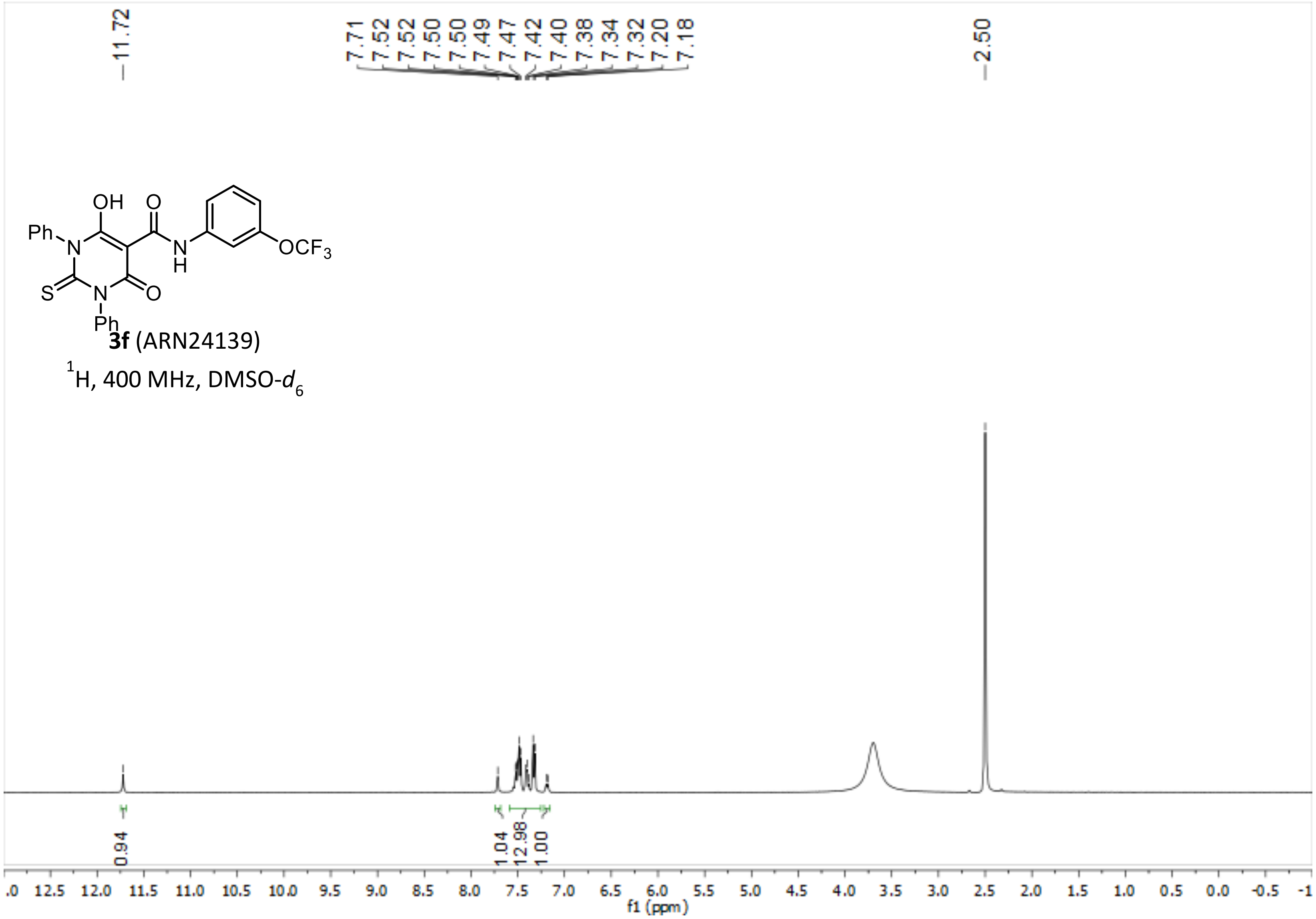
OH
O
Ph
N
N
H
OCF3
S
N
O
Ph
3f (ARN24139)
1H, 400 MHz, DMSO-d6
11.72
7.71
7.52
7.52
7.50
7.50
7.49
7.47
7.42
7.40
7.38
7.34
7.32
7.20
7.18
2.50
0.94
1.04
12.98
1.00
12.5
12.0
11.5
11.0
10.5
10.0
9.5
9.0
8.5
8.0
7.5
7.0
6.5
6.0
5.5
5.0
4.5
4.0
3.5
3.0
2.5
2.0
1.5
1.0
0.5
0.0
-0.5
f1 (ppm)

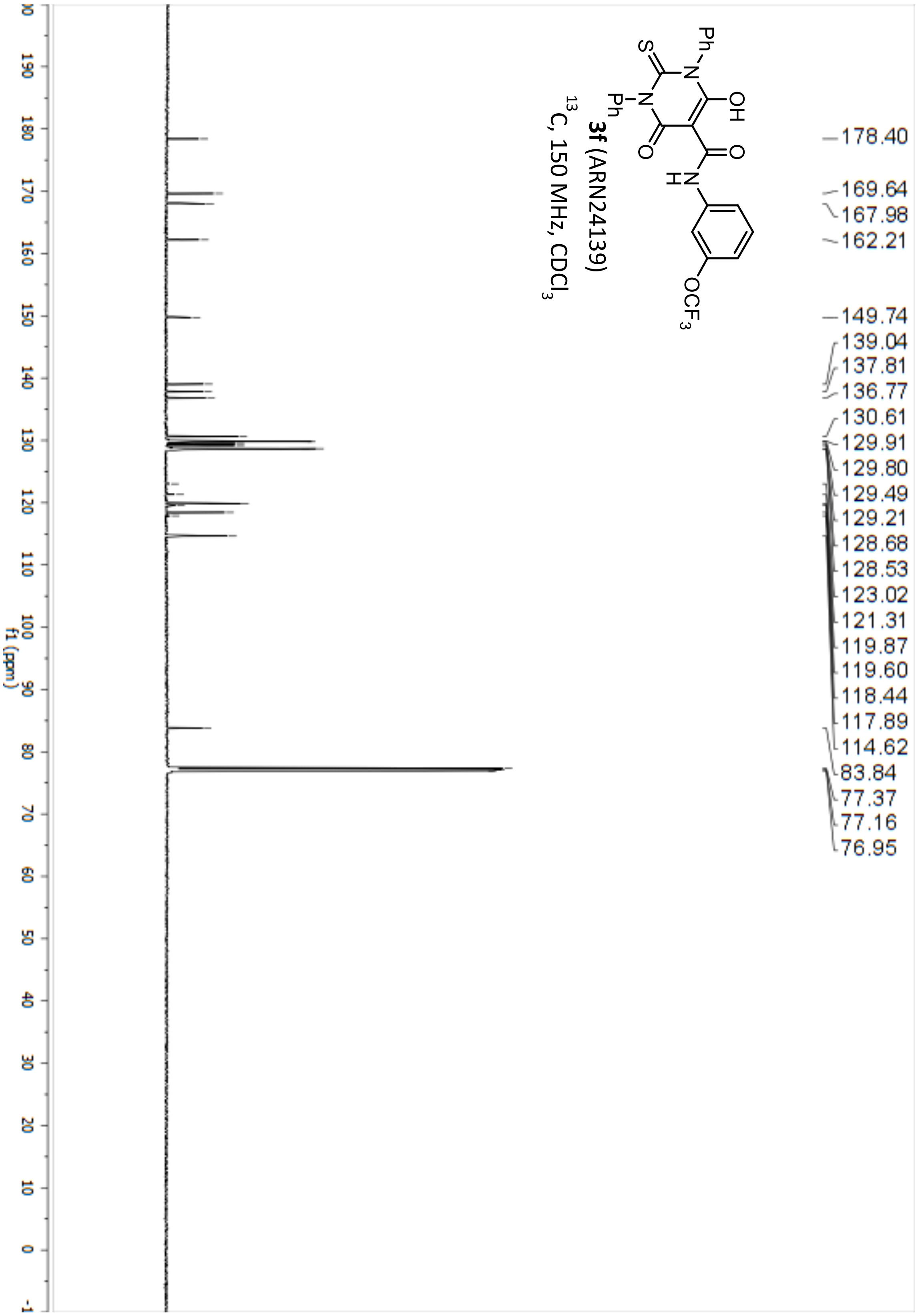
Ph
OH
O
S
N
N
Ph
O
N
H
OCF3
3f (ARN24139)
13C, 150 MHz, CDCl3
178.40
169.64
167.98
162.21
149.74
139.04
137.81
136.77
130.61
129.91
129.80
129.49
129.21
128.68
128.53
123.02
121.31
119.87
119.60
118.44
117.89
114.62
83.84
77.37
77.16
76.95
f1 (ppm)

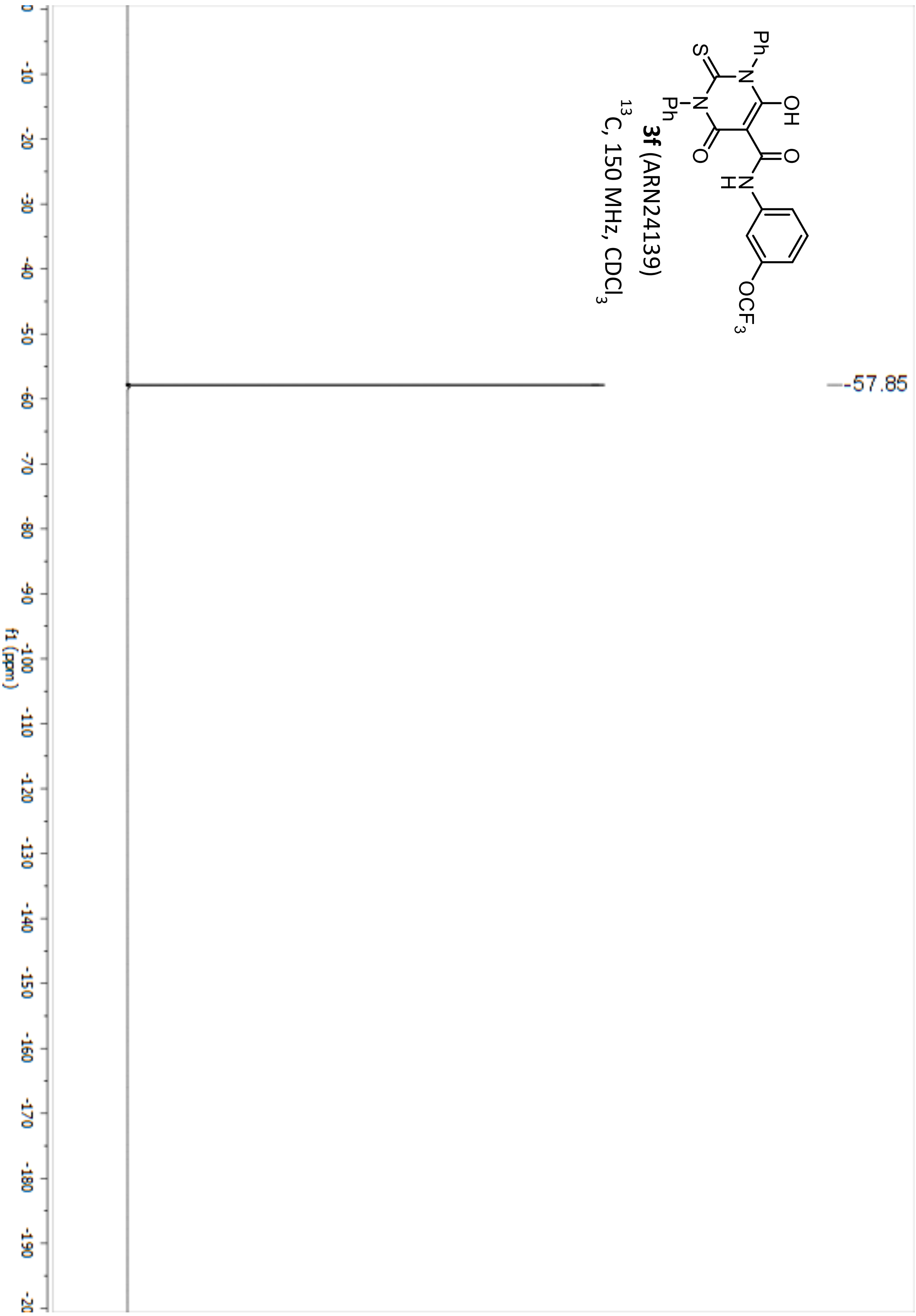
3f (ARN24139)
13C, 150 MHz, CDCl3
-57.85
f1 (ppm)

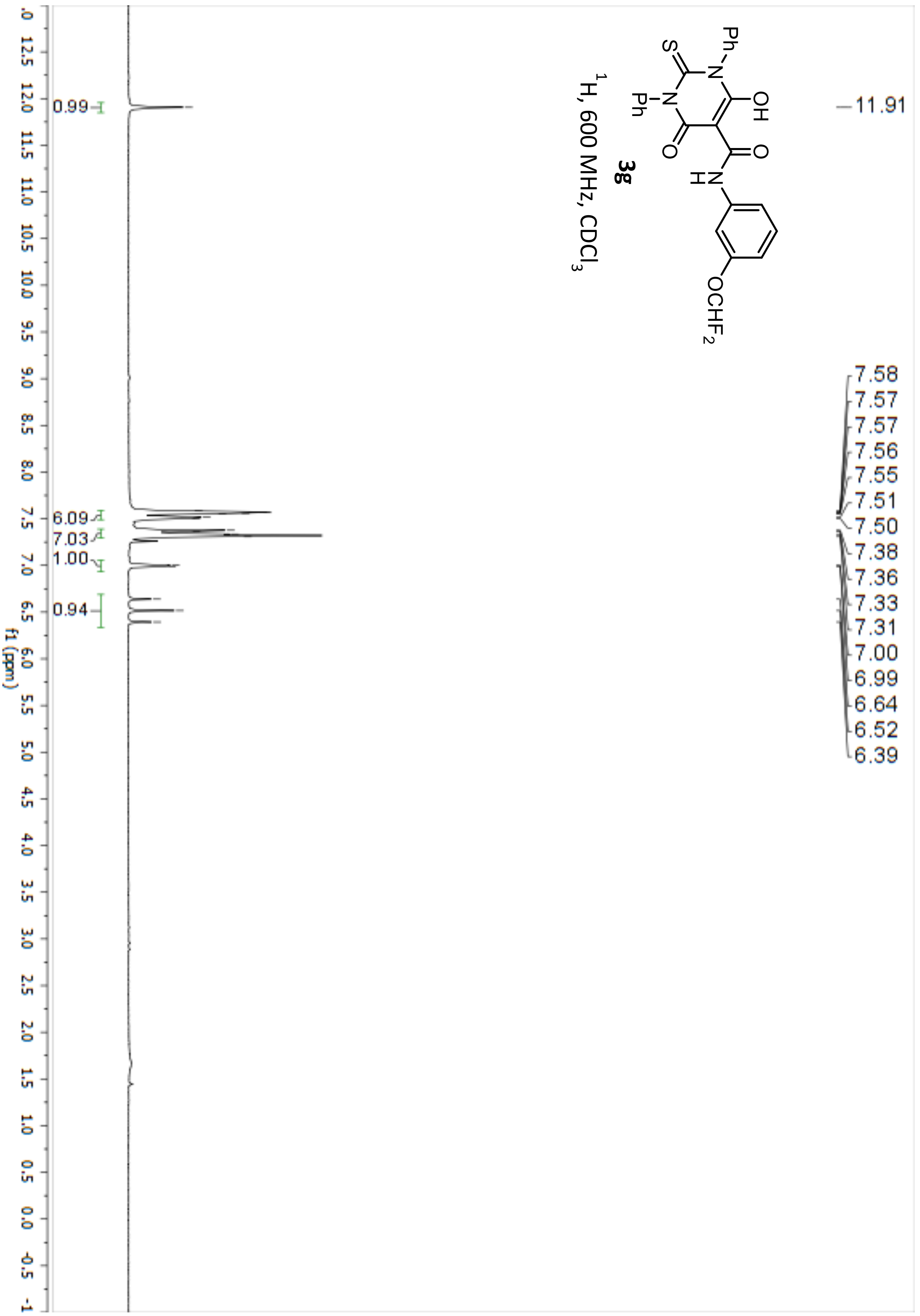
3g
1H, 600 MHz, CDCl3
OH
O
N
H
OCHF2
Ph
S
Ph
11.91
7.58
7.57
7.57
7.56
7.55
7.51
7.50
7.38
7.36
7.33
7.31
7.00
6.99
6.64
6.52
6.39
0.99
6.09
7.03
1.00
0.94
f1 (ppm)

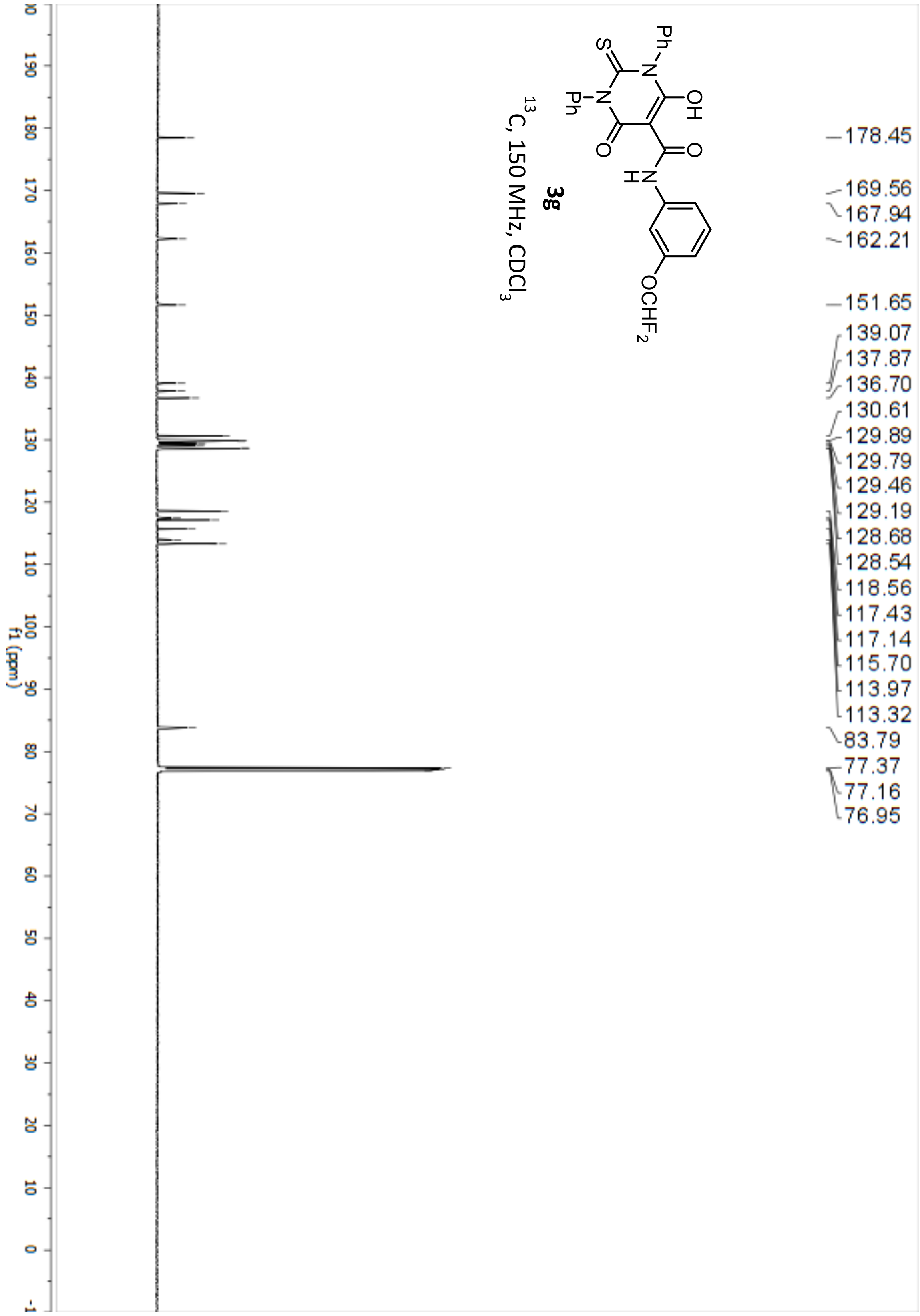
3g
13C, 150 MHz, CDCl3
f1 (ppm)
178.45
169.56
167.94
162.21
151.65
139.07
137.87
136.70
130.61
129.89
129.79
129.46
129.19
128.68
128.54
118.56
117.43
117.14
115.70
113.97
113.32
83.79
77.37
77.16
76.95

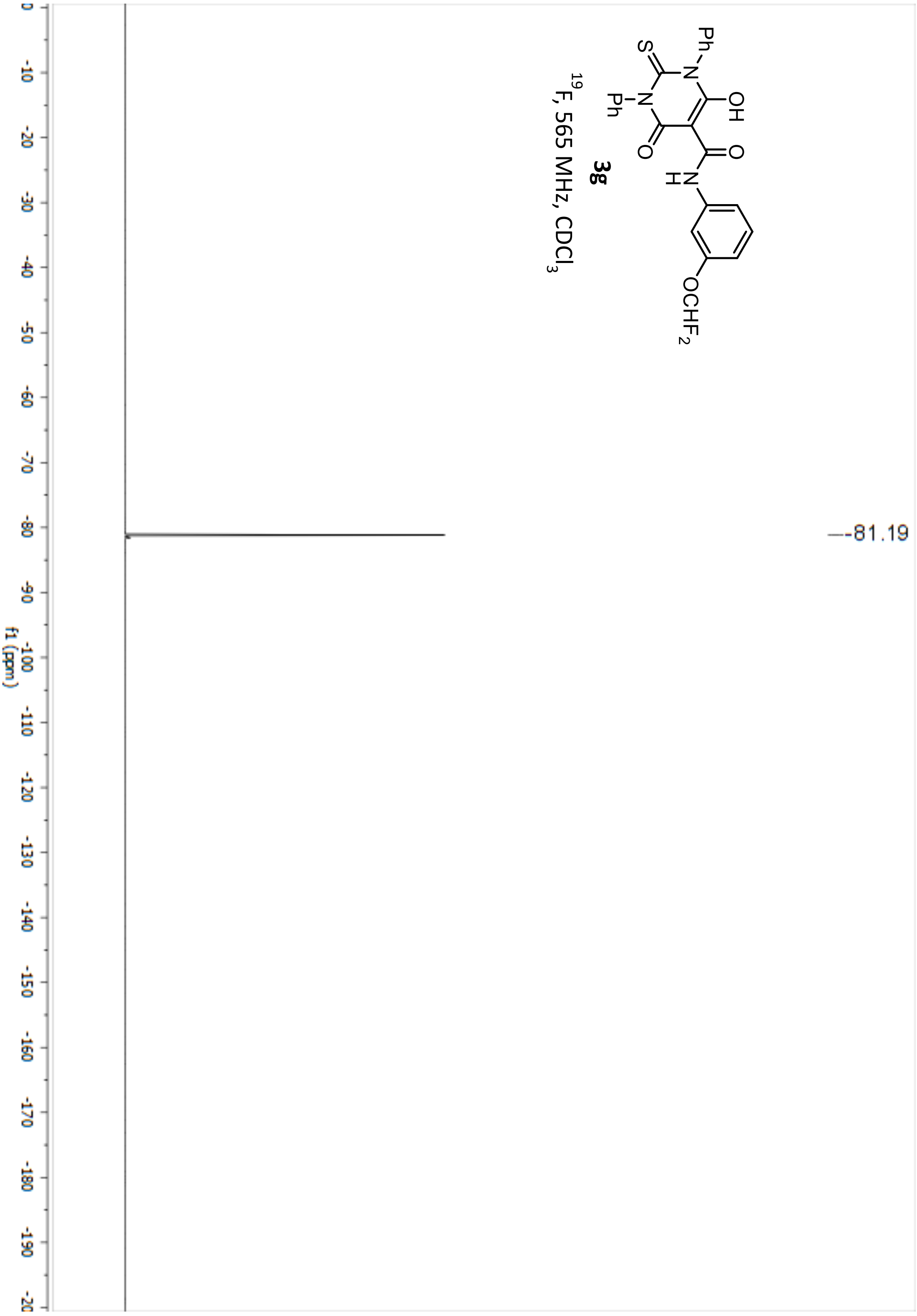
3g
19F, 565 MHz, CDCl3
-81.19
f1 (ppm)

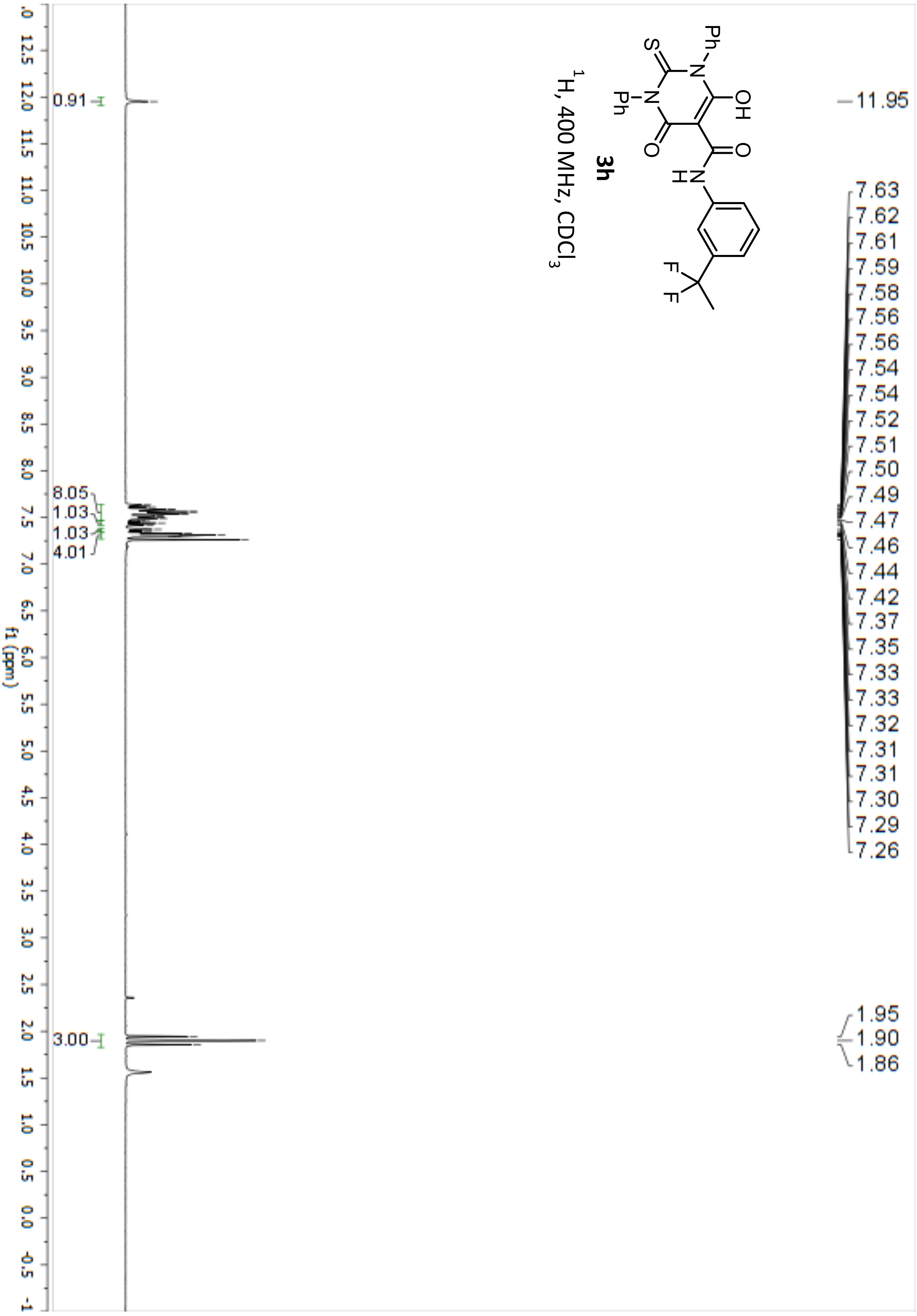
3h
1H, 400 MHz, CDCl3

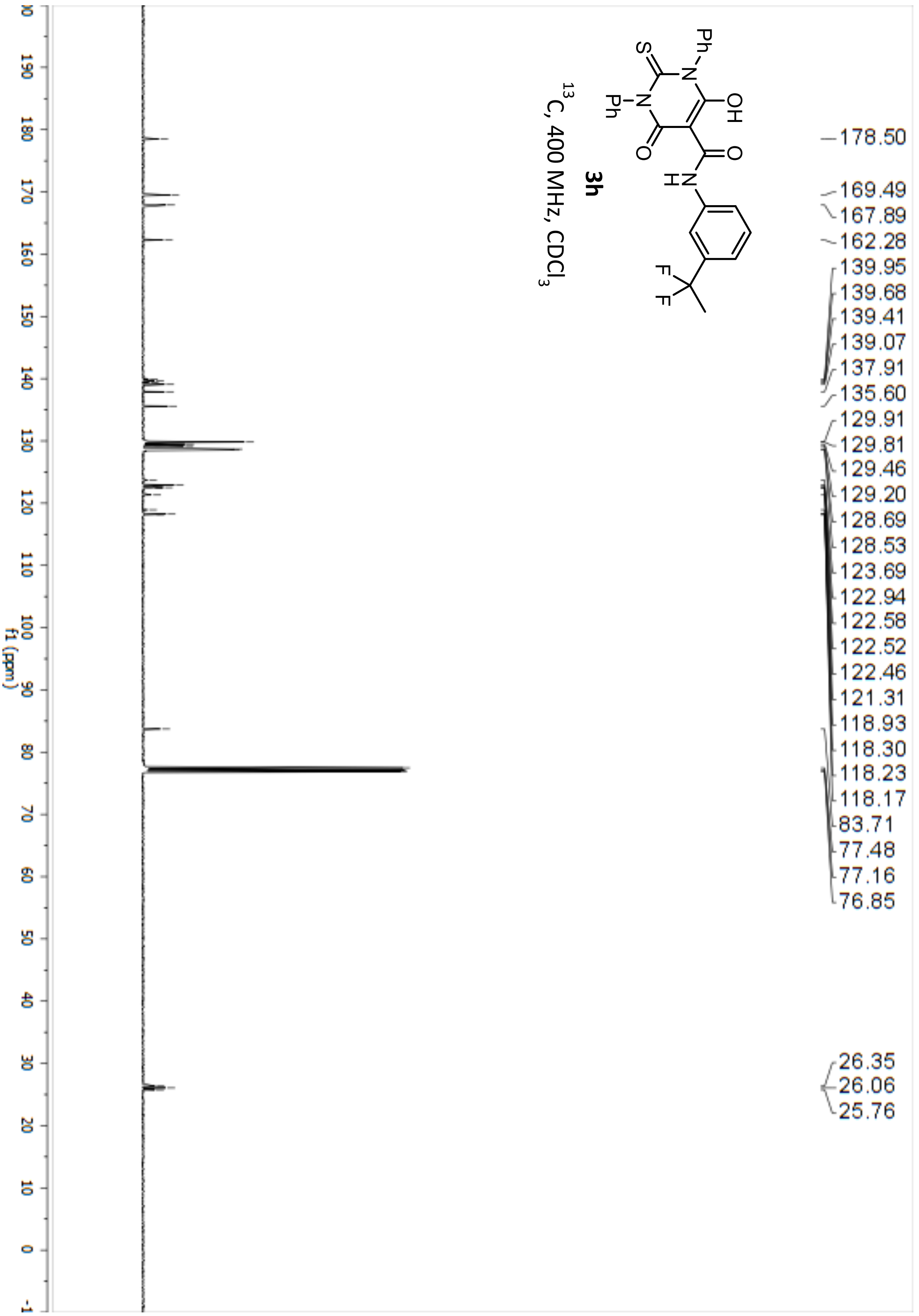
3h
13C, 400 MHz, CDCl3
178.50
169.49
167.89
162.28
139.95
139.68
139.41
139.07
137.91
135.60
129.91
129.81
129.46
129.20
128.69
128.53
123.69
122.94
122.58
122.52
122.46
121.31
118.93
118.30
118.23
118.17
83.71
77.48
77.16
76.85
26.35
26.06
25.76
f1 (ppm)

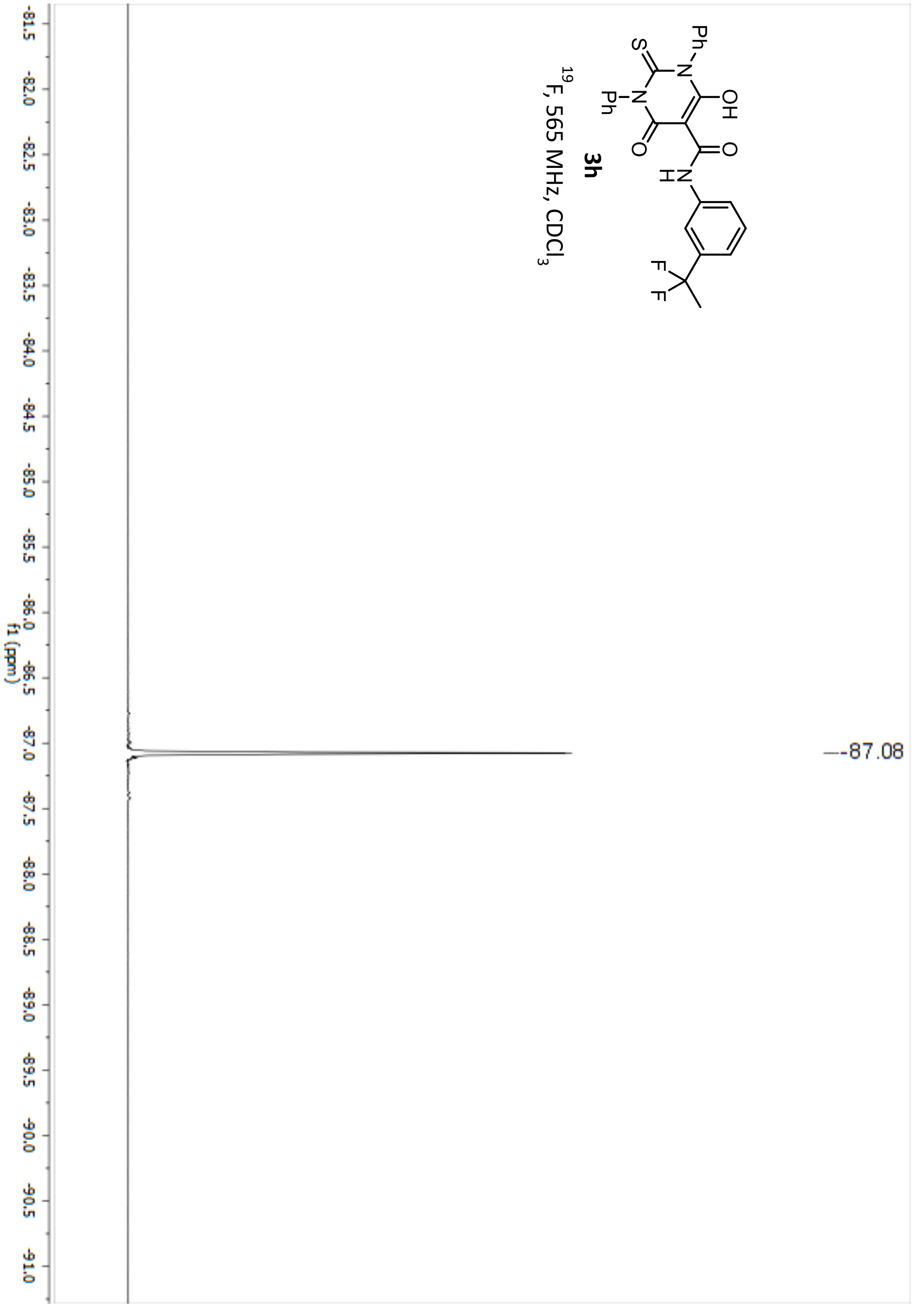

3h
19F, 565 MHz, CDCl3
-87.08
f1 (ppm)

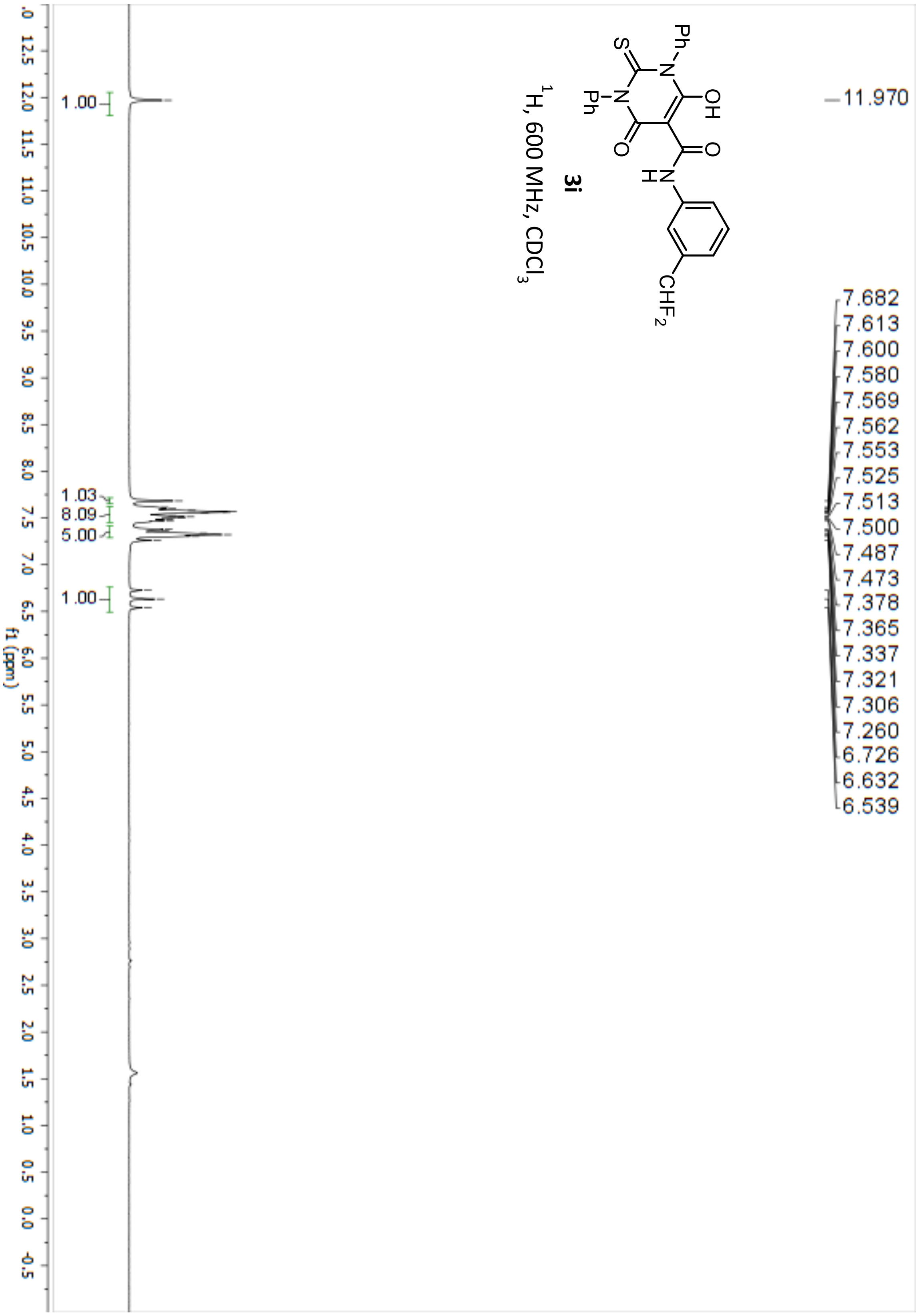

3i
1H, 600 MHz, CDCl3
11.970
7.682
7.613
7.600
7.580
7.569
7.562
7.553
7.525
7.513
7.500
7.487
7.473
7.378
7.365
7.337
7.321
7.306
7.260
6.726
6.632
6.539
f1 (ppm)

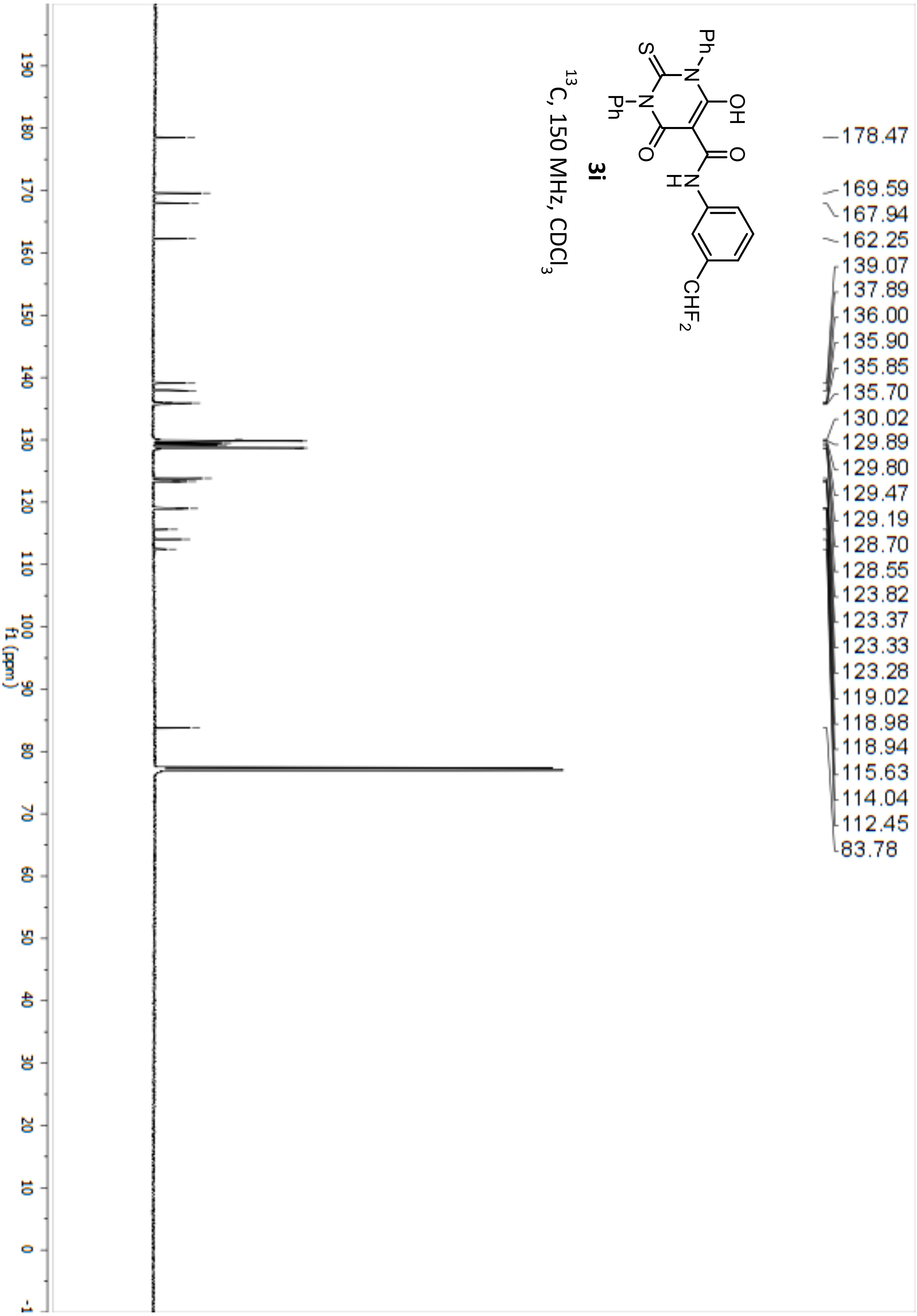
3i
13C, 150 MHz, CDCl3
178.47
169.59
167.94
162.25
139.07
137.89
136.00
135.90
135.85
135.70
130.02
129.89
129.80
129.47
129.19
128.70
128.55
123.82
123.37
123.33
123.28
119.02
118.98
118.94
115.63
114.04
112.45
83.78
f1 (ppm)

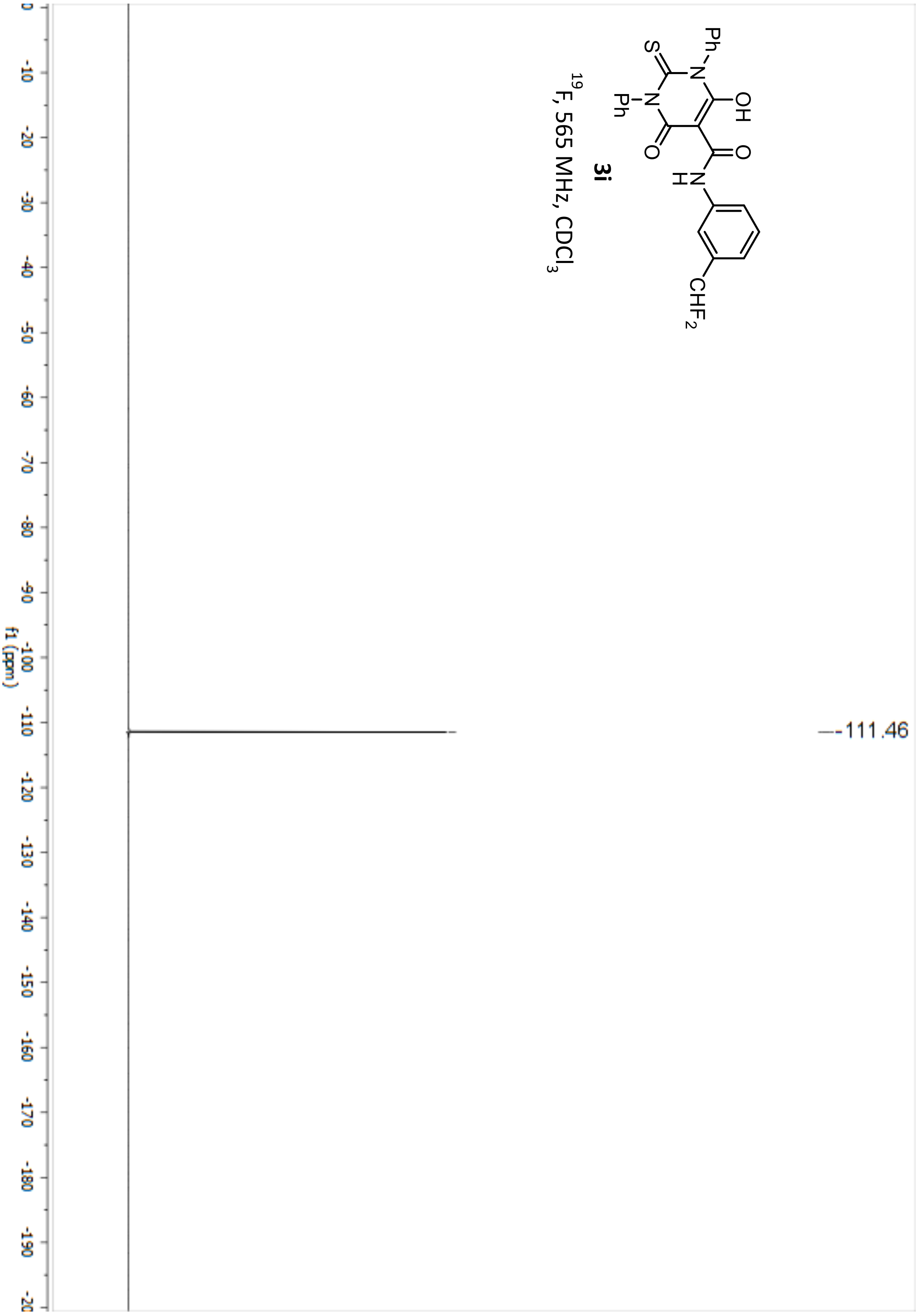

OH
O
Ph
N
S
N
Ph
O
N
H
CHF_2
3i
^{19}F, 565 MHz, $CDCl_3$
-111.46
f1 (ppm)
0
-10
-20
-30
-40
-50
-60
-70
-80
-90
-100
-110
-120
-130
-140
-150
-160
-170
-180
-190
-200

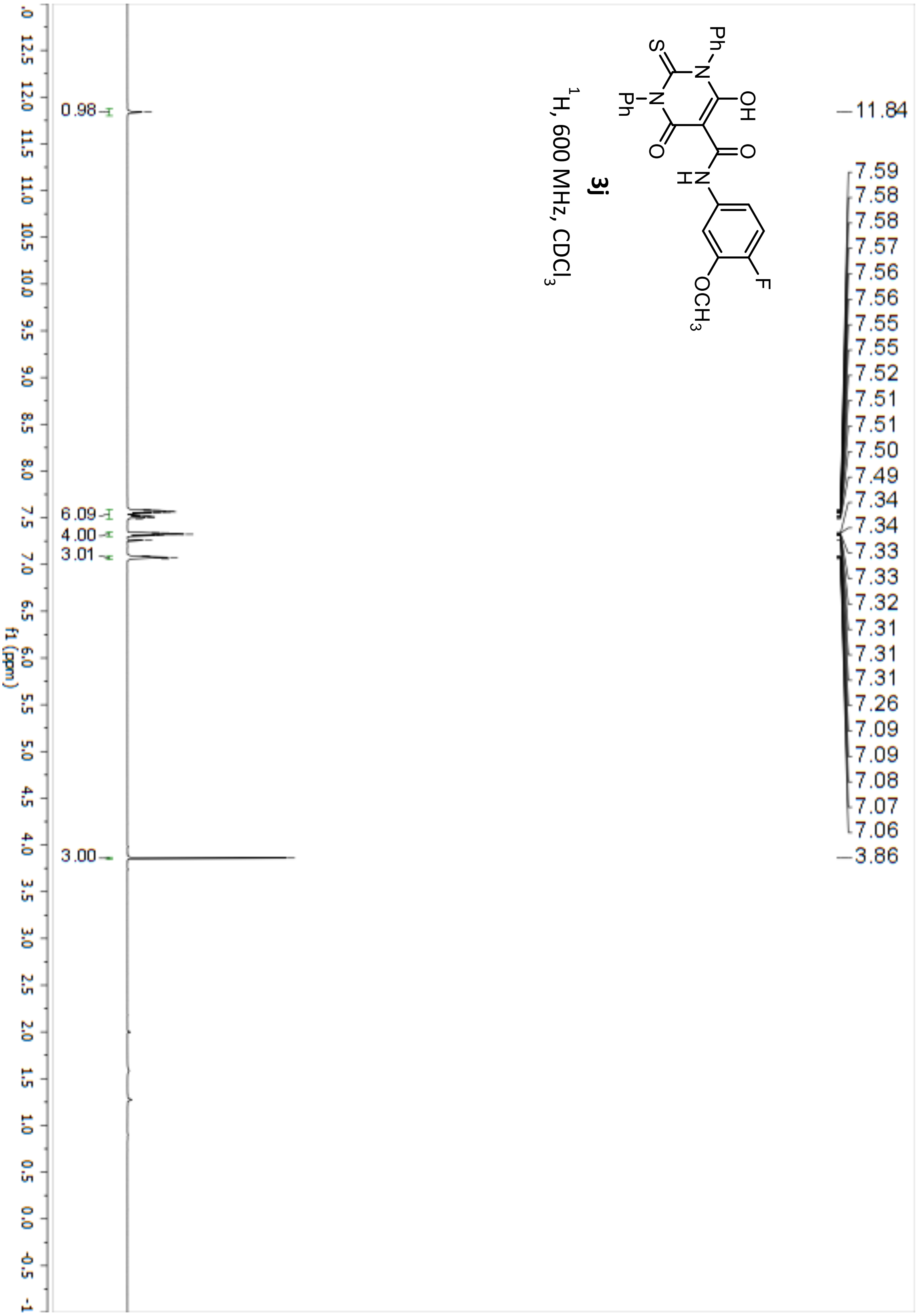

3j
1H, 600 MHz, CDCl3
11.84
7.59
7.58
7.58
7.57
7.56
7.56
7.55
7.55
7.52
7.51
7.51
7.50
7.49
7.34
7.34
7.33
7.33
7.32
7.31
7.31
7.31
7.26
7.09
7.09
7.08
7.07
7.06
3.86
0.98
6.09
4.00
3.01
3.00
f1 (ppm)

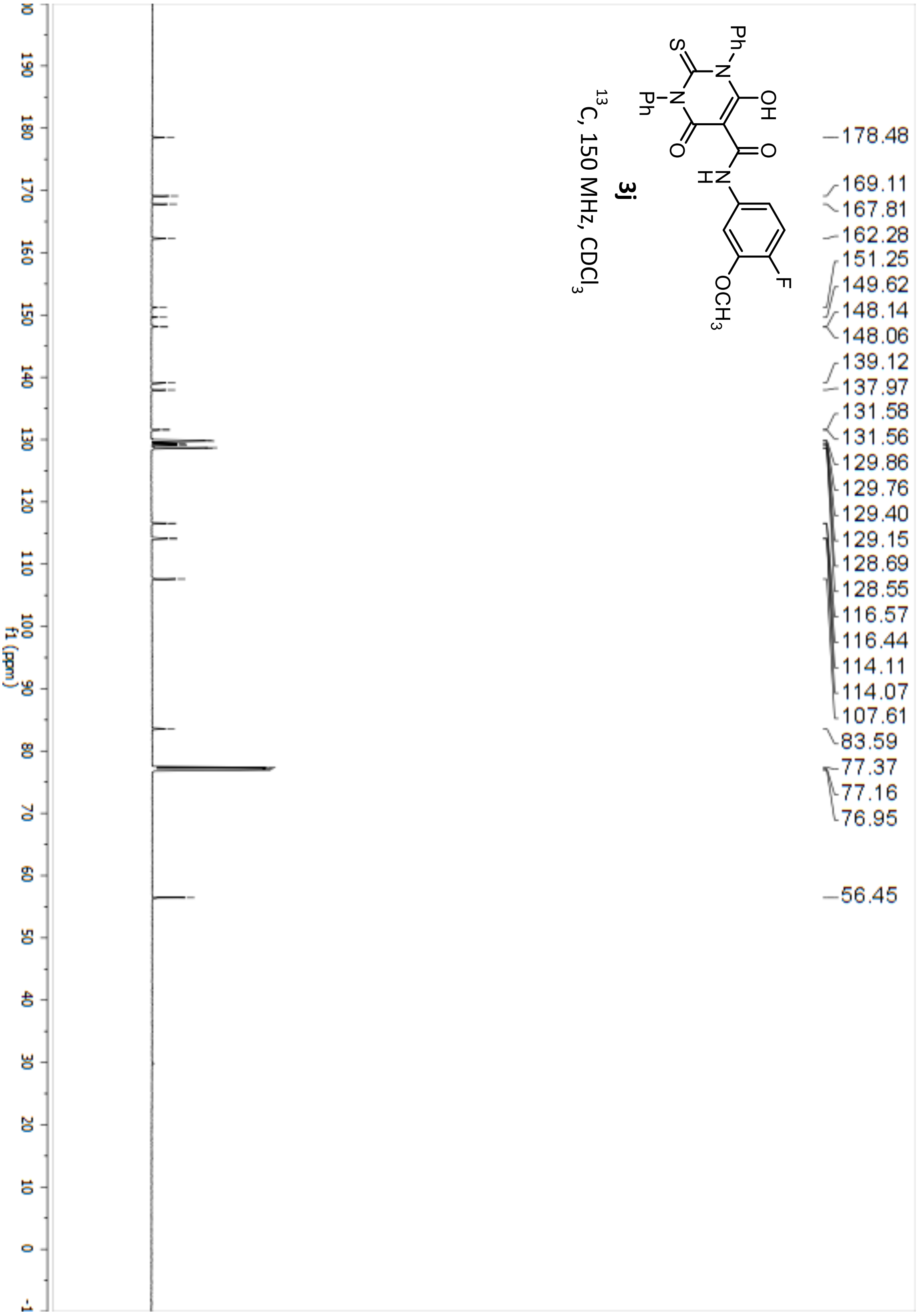

3j
^{13}C, 150 MHz, $CDCl_3$
Ph
S
N
N
OH
O
H
F
OCH_3
178.48
169.11
167.81
162.28
151.25
149.62
148.14
148.06
139.12
137.97
131.58
131.56
129.86
129.76
129.40
129.15
128.69
128.55
116.57
116.44
114.11
114.07
107.61
83.59
77.37
77.16
76.95
56.45
f1 (ppm)

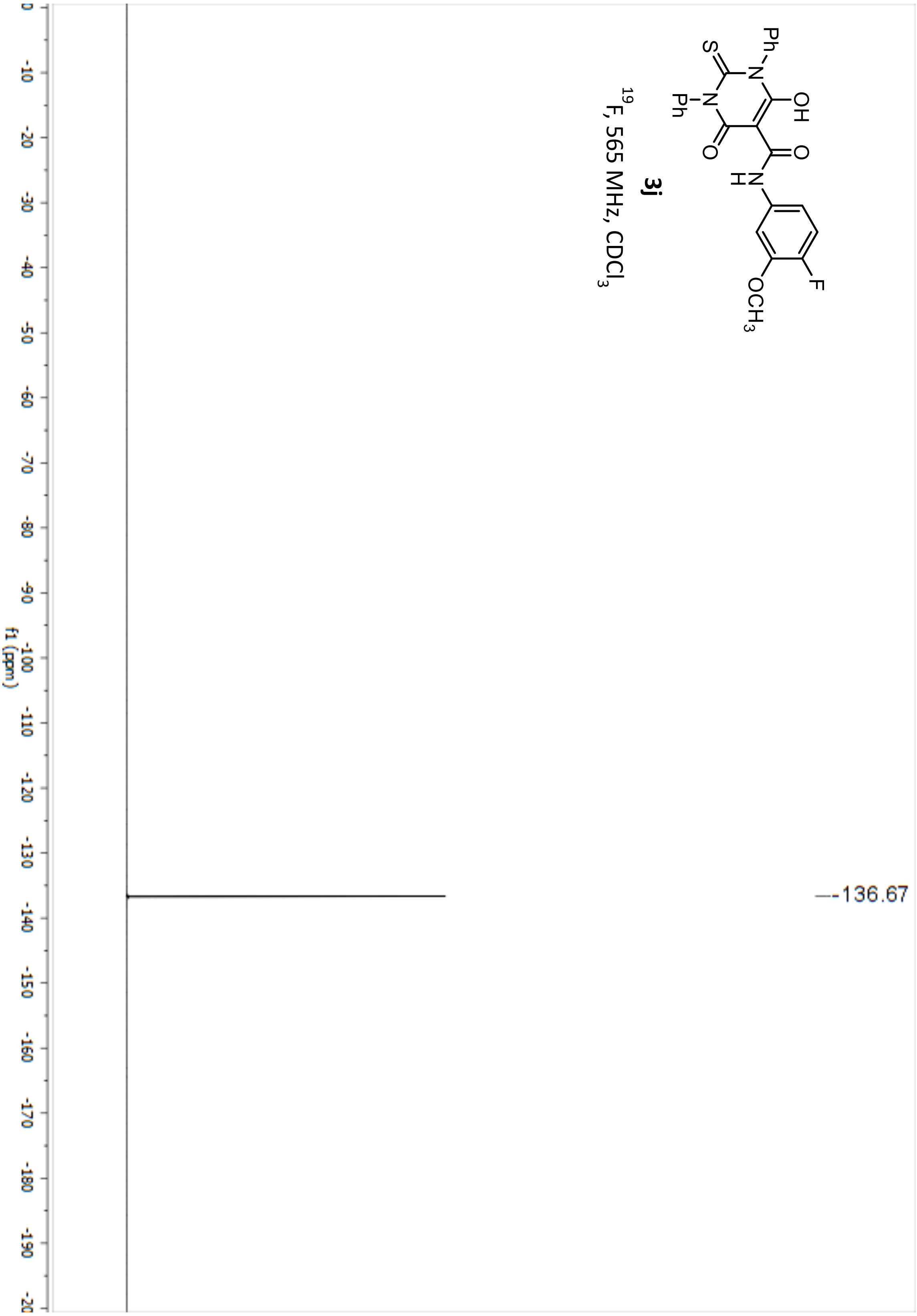
3j
19F, 565 MHz, CDCl3
-136.67
f1 (ppm)

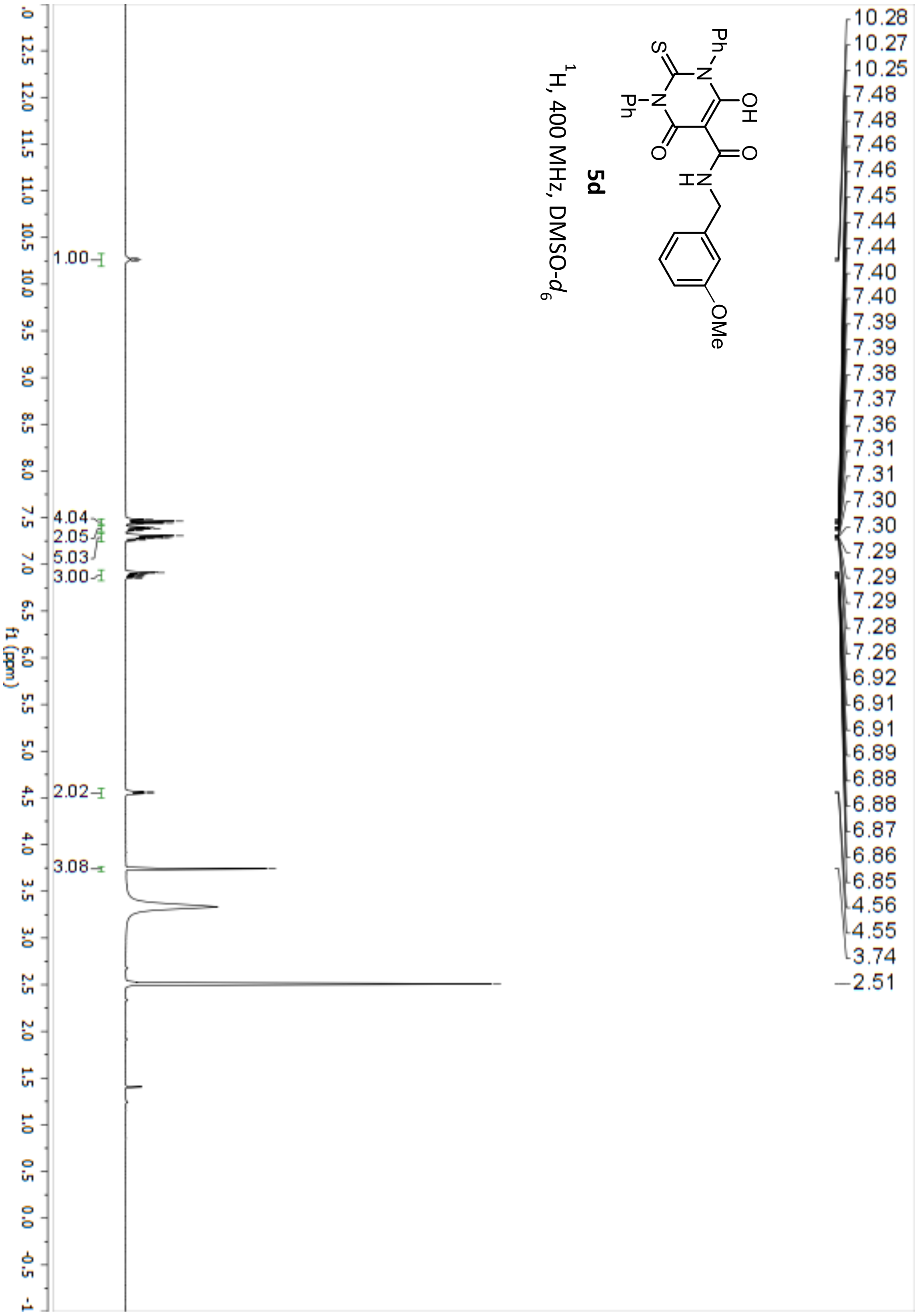
10.28
10.27
10.25
7.48
7.48
7.46
7.46
7.45
7.44
7.44
7.40
7.40
7.39
7.39
7.38
7.37
7.36
7.31
7.31
7.30
7.30
7.29
7.29
7.29
7.28
7.26
6.92
6.91
6.91
6.89
6.88
6.88
6.87
6.86
6.85
4.56
4.55
3.74
2.51
Ph
S
N
OH
O
N
H
OMe
5d
1H, 400 MHz, DMSO-d6
1.00
4.04
2.05
5.03
3.00
2.02
3.08
f1 (ppm)

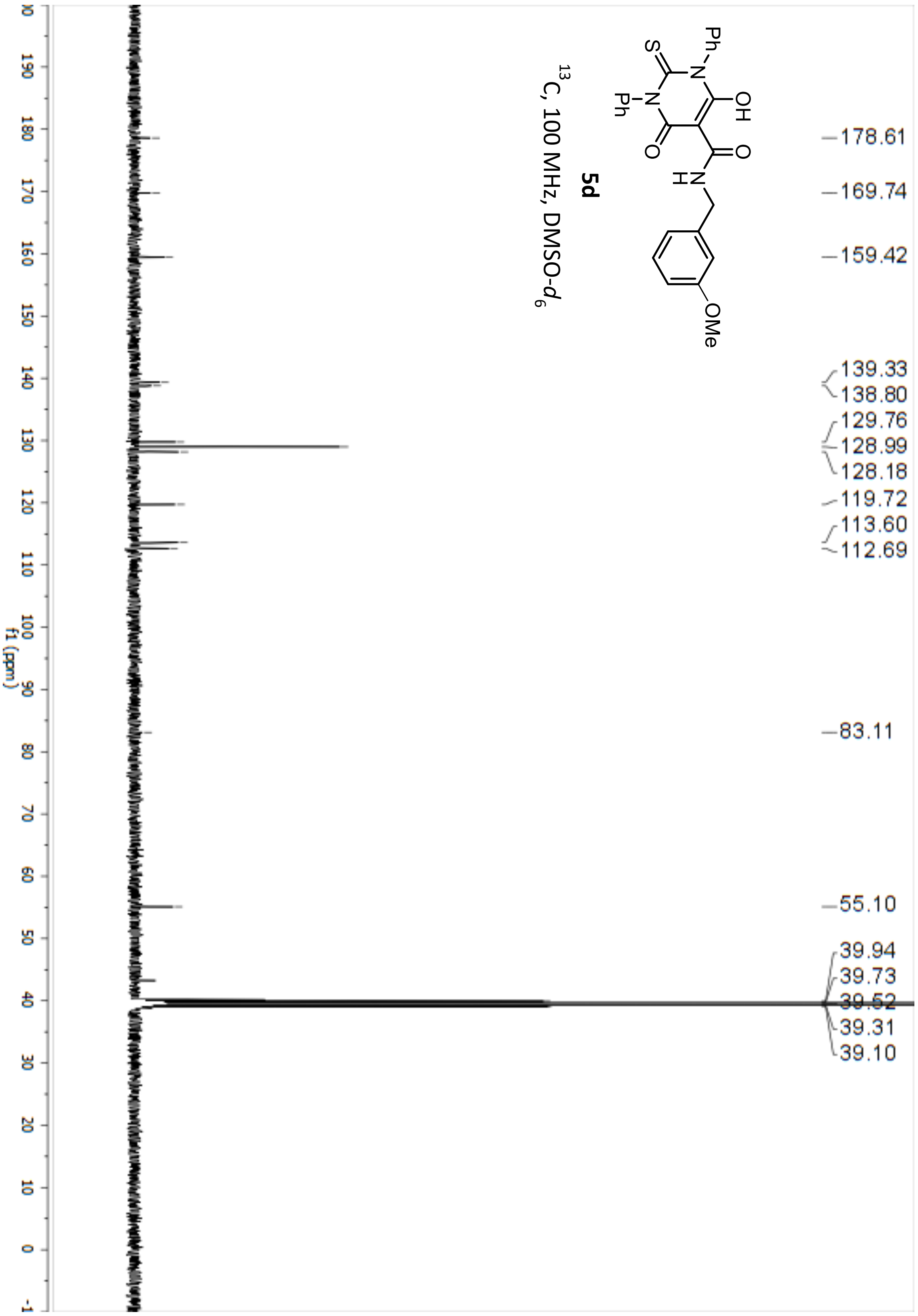

5d
^{13}C, 100 MHz, DMSO-d_6
178.61
169.74
159.42
139.33
138.80
129.76
128.99
128.18
119.72
113.60
112.69
83.11
55.10
39.94
39.73
39.52
39.31
39.10
f1 (ppm)

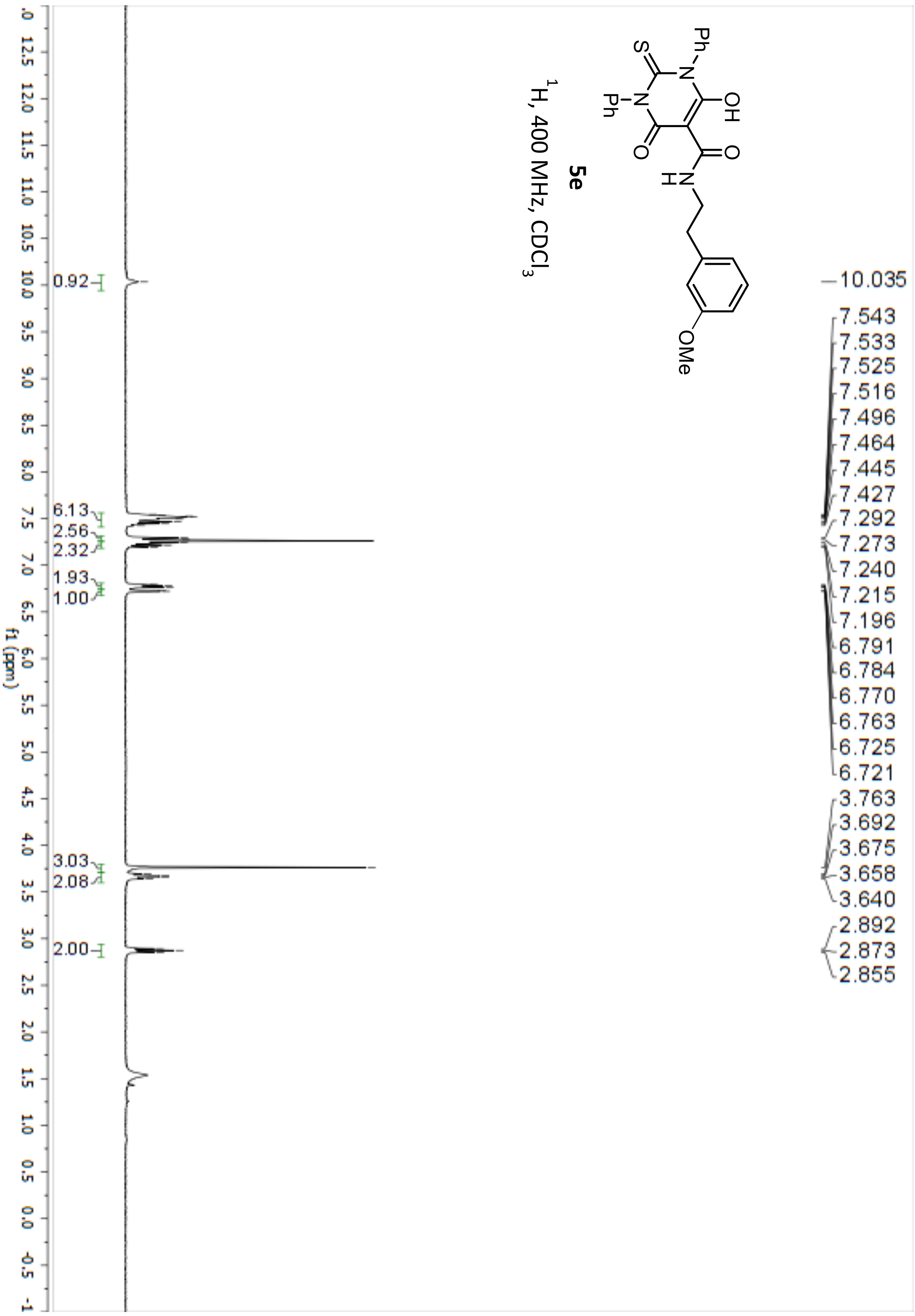
5e
1H, 400 MHz, CDCl3
OH
O
Ph
N
S
N
Ph
O
N
H
OMe
10.035
7.543
7.533
7.525
7.516
7.496
7.464
7.445
7.427
7.292
7.273
7.240
7.215
7.196
6.791
6.784
6.770
6.763
6.725
6.721
3.763
3.692
3.675
3.658
3.640
2.892
2.873
2.855
0.92
6.13
2.56
2.32
1.93
1.00
3.03
2.08
2.00
f1 (ppm)

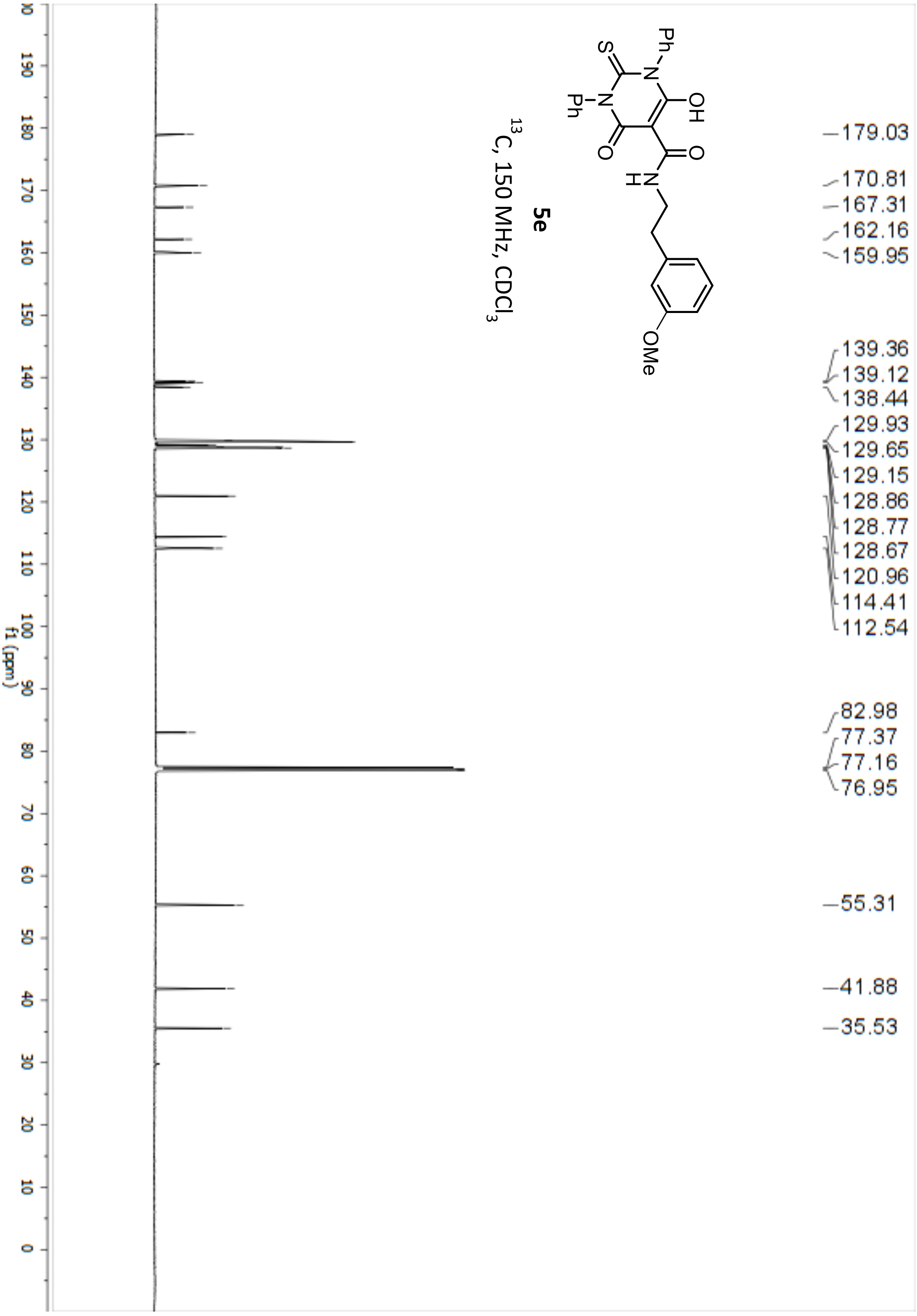
5e
13C, 150 MHz, CDCl3
179.03
170.81
167.31
162.16
159.95
139.36
139.12
138.44
129.93
129.65
129.15
128.86
128.77
128.67
120.96
114.41
112.54
82.98
77.37
77.16
76.95
55.31
41.88
35.53
f1 (ppm)

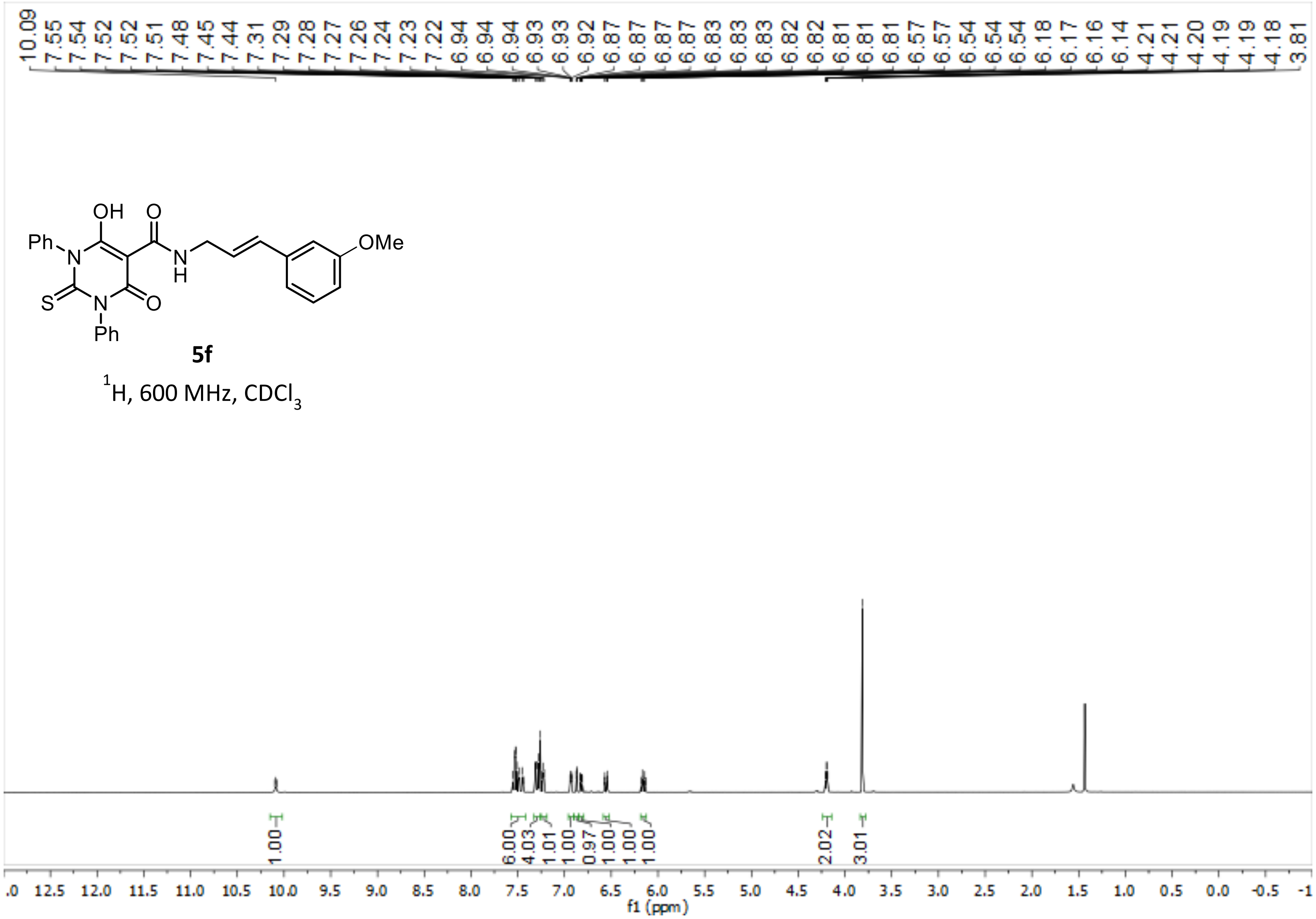
10.09
7.55
7.54
7.52
7.52
7.51
7.48
7.45
7.44
7.31
7.29
7.28
7.27
7.26
7.24
7.23
7.22
6.94
6.94
6.94
6.93
6.93
6.92
6.87
6.87
6.87
6.87
6.83
6.83
6.83
6.82
6.82
6.81
6.81
6.81
6.57
6.57
6.54
6.54
6.54
6.18
6.17
6.16
6.14
4.21
4.21
4.20
4.19
4.19
4.19
4.18
3.81
OH
O
Ph
N
N
H
OMe
S
N
O
Ph
5f
1H, 600 MHz, CDCl3
1.00
6.00
4.03
1.01
1.00
0.97
1.00
1.00
1.00
2.02
3.01
f1 (ppm)

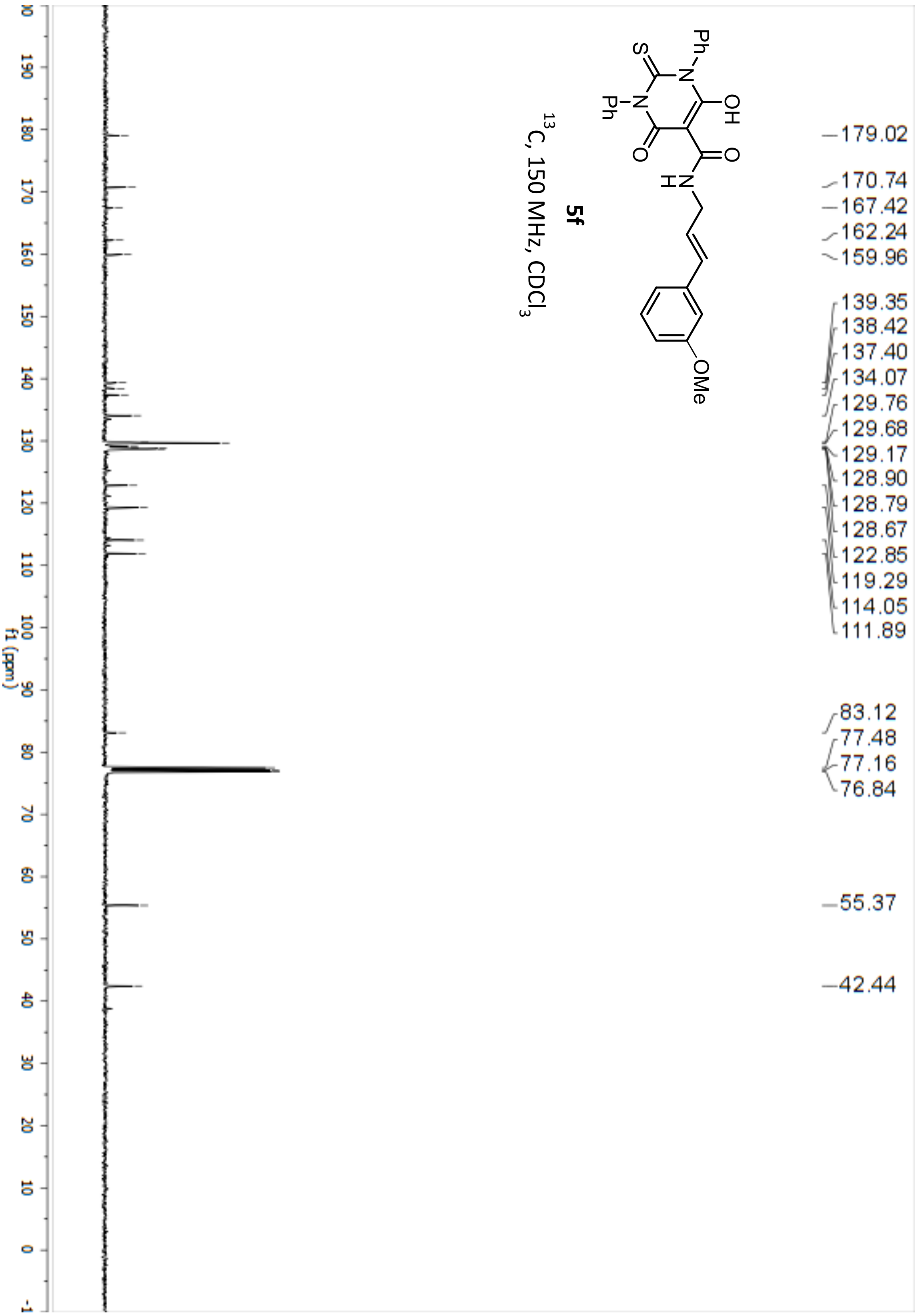
5f
13C, 150 MHz, CDCl3
179.02
170.74
167.42
162.24
159.96
139.35
138.42
137.40
134.07
129.76
129.68
129.17
128.90
128.79
128.67
122.85
119.29
114.05
111.89
83.12
77.48
77.16
76.84
55.37
42.44
f1 (ppm)

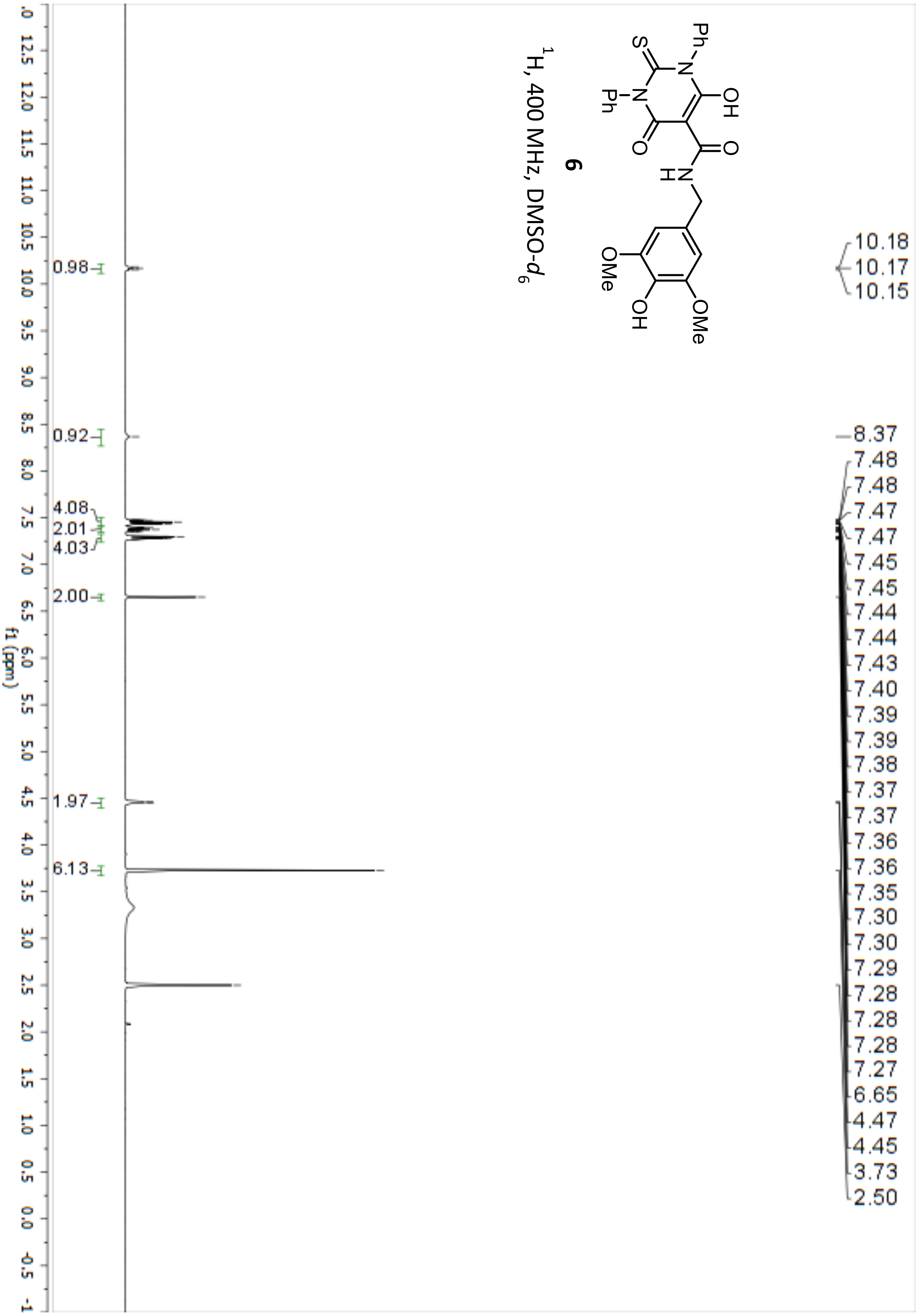
6
1H, 400 MHz, DMSO-d6
10.18
10.17
10.15
8.37
7.48
7.48
7.47
7.47
7.45
7.45
7.44
7.44
7.43
7.40
7.39
7.39
7.38
7.37
7.37
7.36
7.36
7.35
7.30
7.30
7.29
7.28
7.28
7.28
7.27
6.65
4.47
4.45
3.73
2.50
0.98
0.92
4.08
2.01
4.03
2.00
1.97
6.13
f1 (ppm)

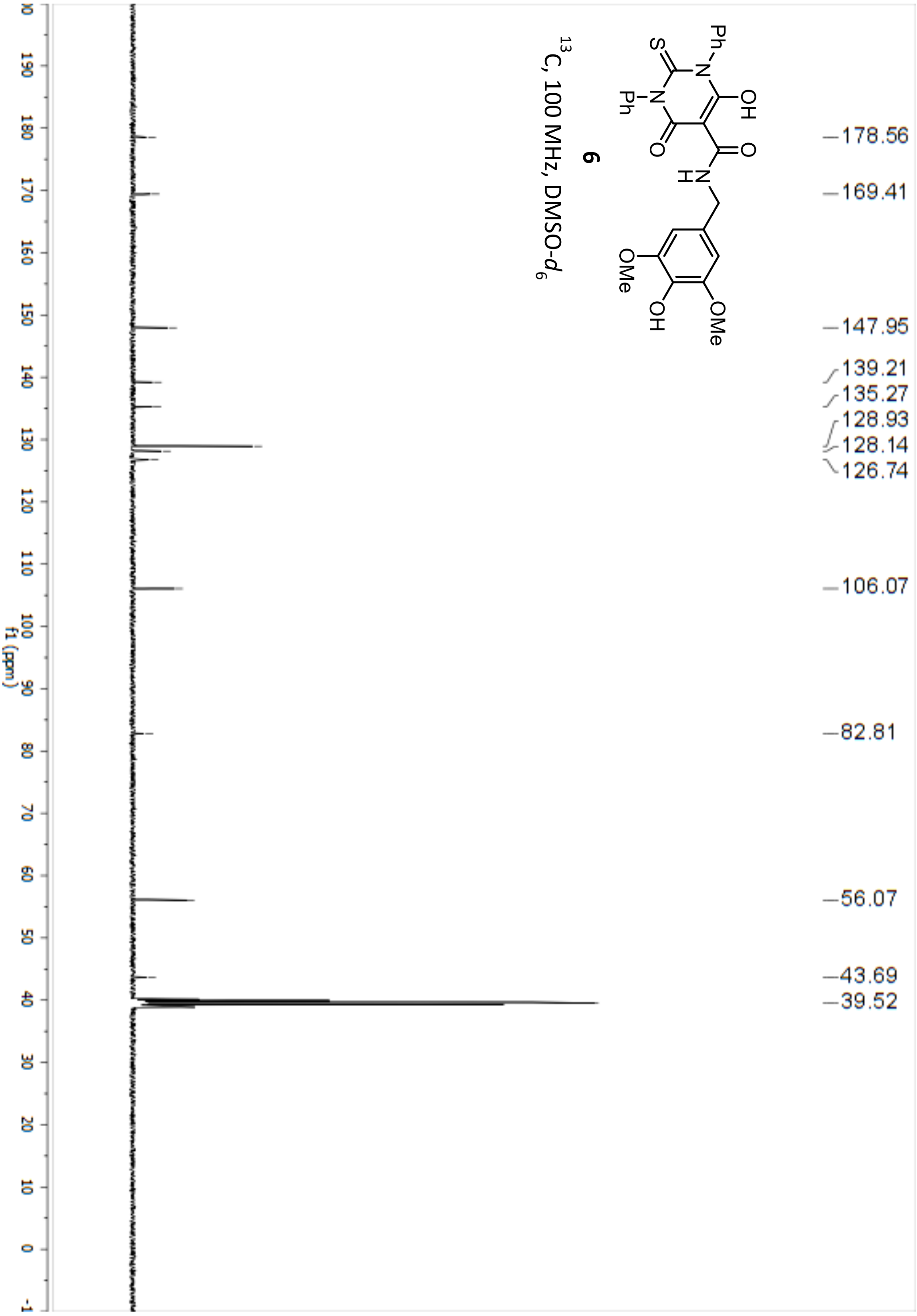
178.56
169.41
147.95
139.21
135.27
128.93
128.14
126.74
106.07
82.81
56.07
43.69
39.52
6
^{13}C, 100 MHz, DMSO-d_6
f1 (ppm)

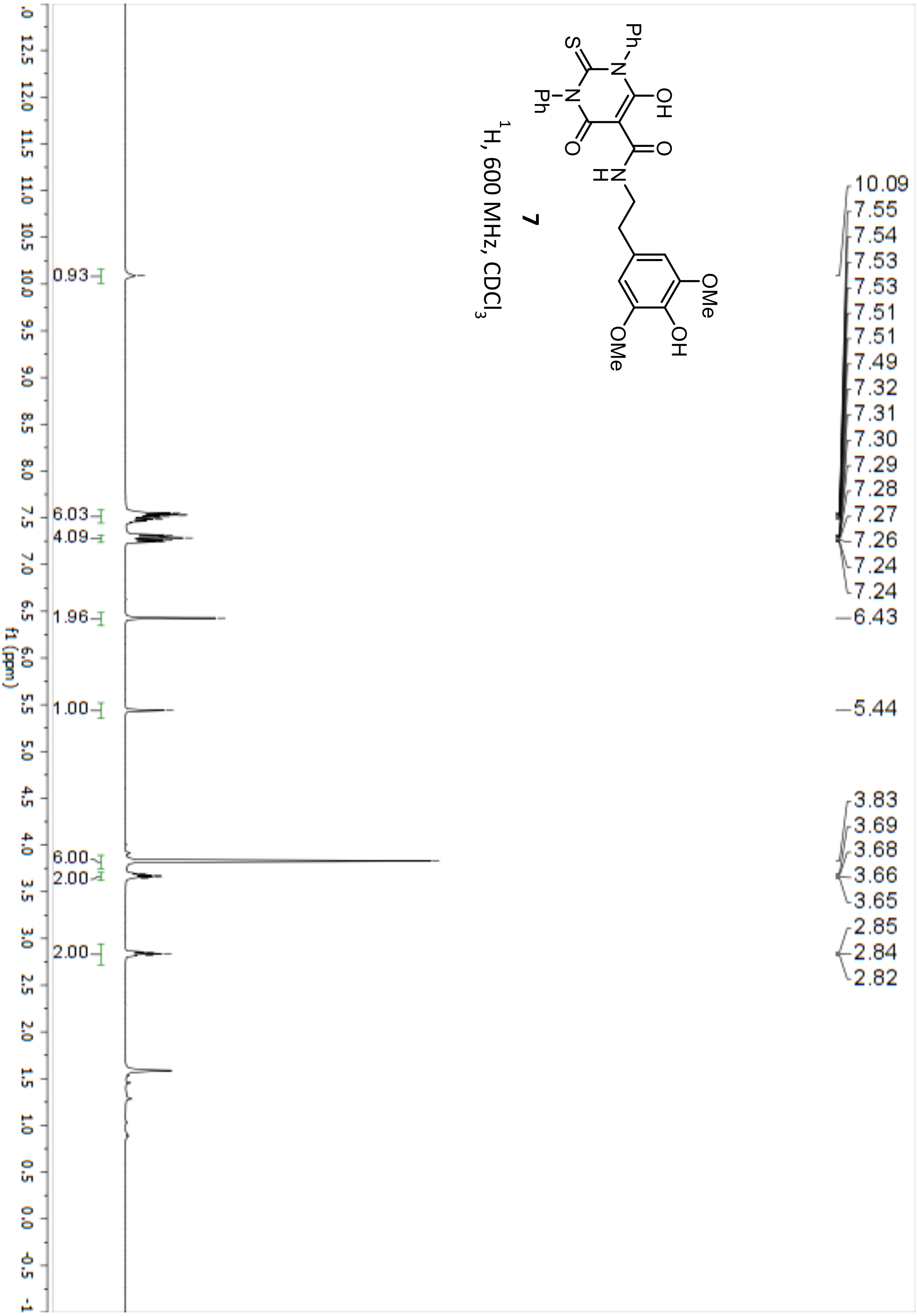
7
1H, 600 MHz, CDCl3
10.09
7.55
7.54
7.53
7.53
7.51
7.51
7.49
7.32
7.31
7.30
7.29
7.28
7.27
7.26
7.24
7.24
6.43
5.44
3.83
3.69
3.68
3.66
3.65
2.85
2.84
2.82
0.93
6.03
4.09
1.96
1.00
6.00
2.00
2.00
f1 (ppm)

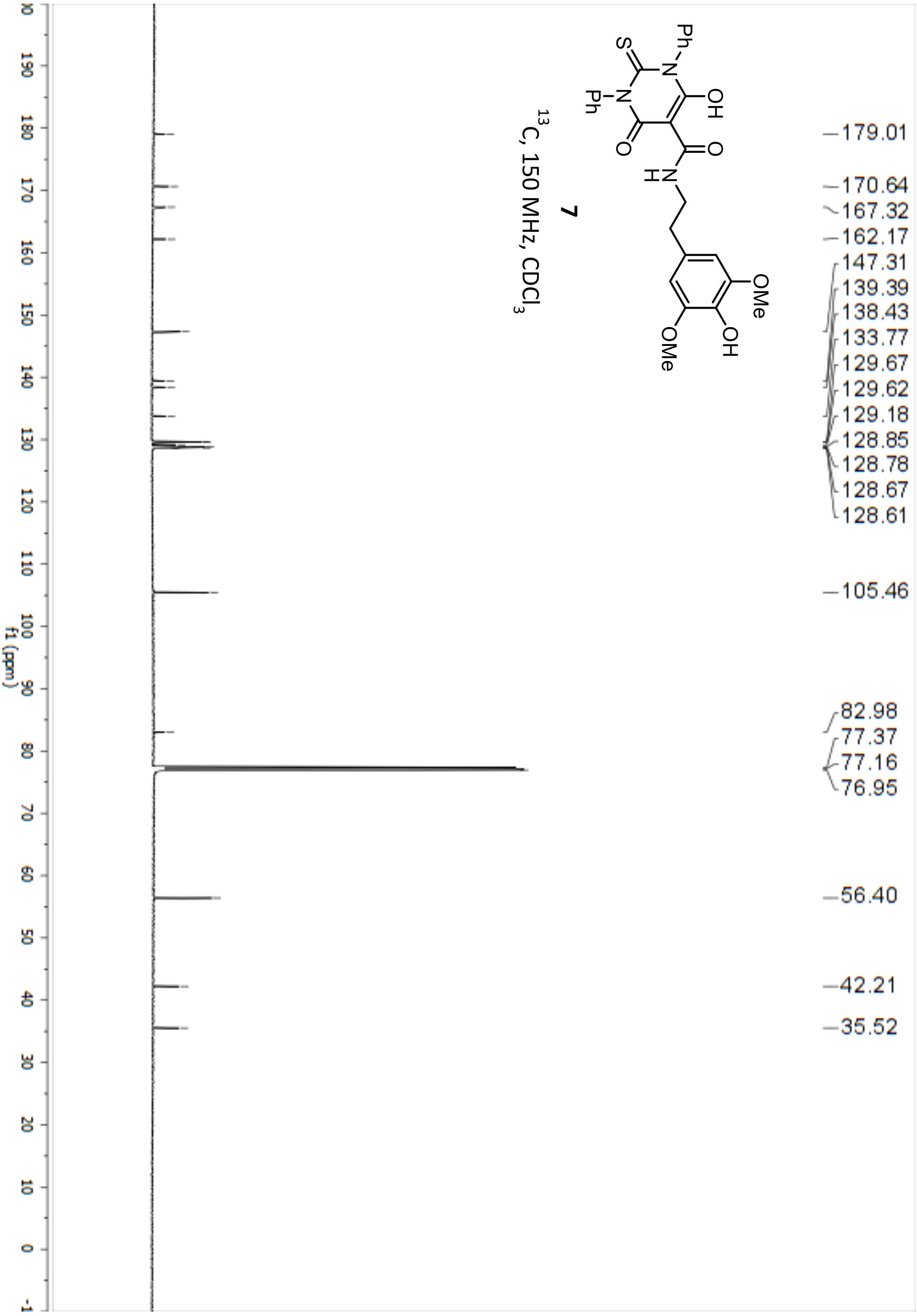
7
^{13}C, 150 MHz, $CDCl_3$
OH
O
N
H
OMe
OH
OMe
Ph
S
Ph
O
179.01
170.64
167.32
162.17
147.31
139.39
138.43
133.77
129.67
129.62
129.18
128.85
128.78
128.67
128.61
105.46
82.98
77.37
77.16
76.95
56.40
42.21
35.52
f1 (ppm)

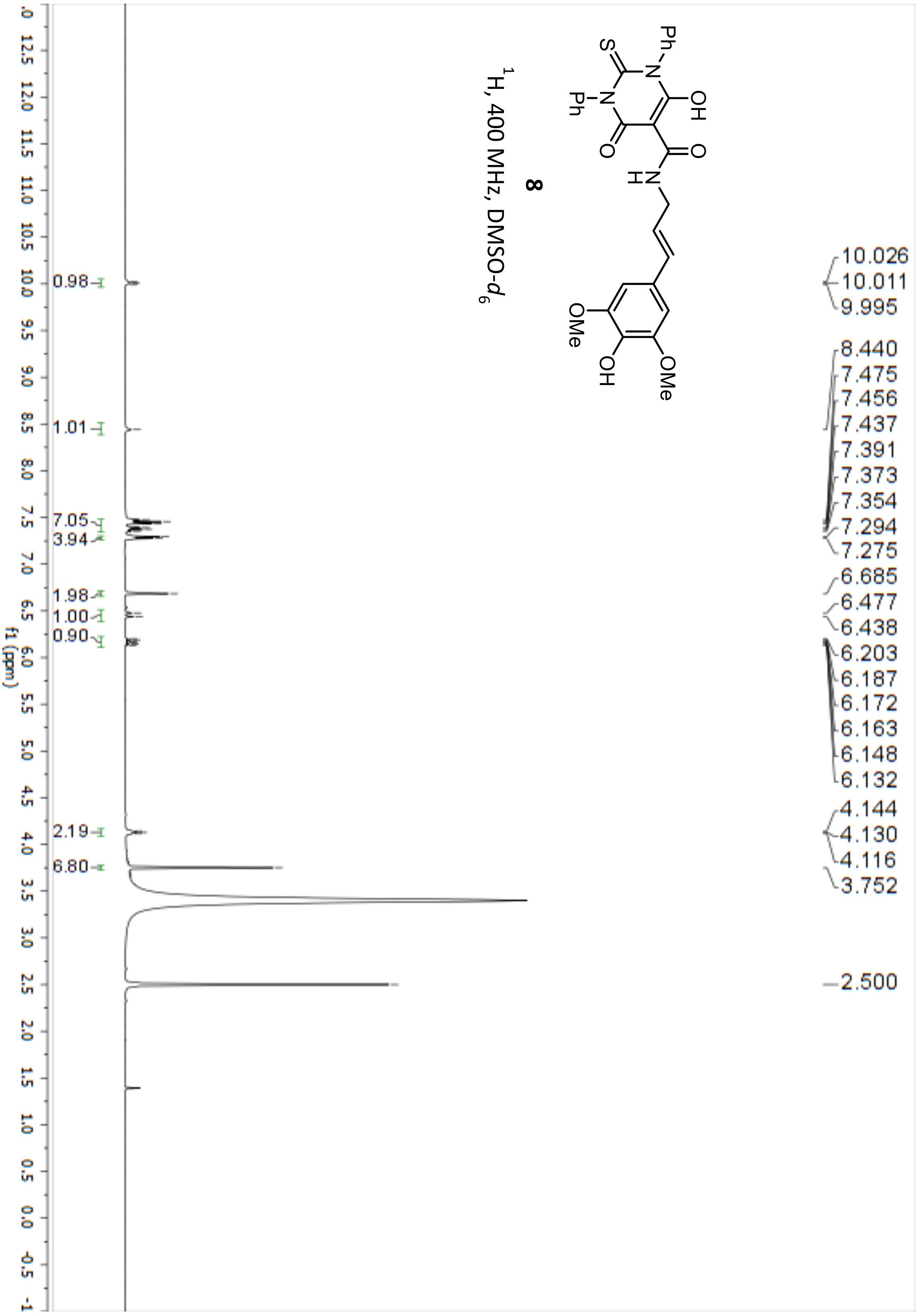
8
^{1}H, 400 MHz, DMSO-d_6
10.026
10.011
9.995
8.440
7.475
7.456
7.437
7.391
7.373
7.354
7.294
7.275
6.685
6.477
6.438
6.203
6.187
6.172
6.163
6.148
6.132
4.144
4.130
4.116
3.752
2.500
0.98
1.01
7.05
3.94
1.98
1.00
0.90
2.19
6.80
f1 (ppm)

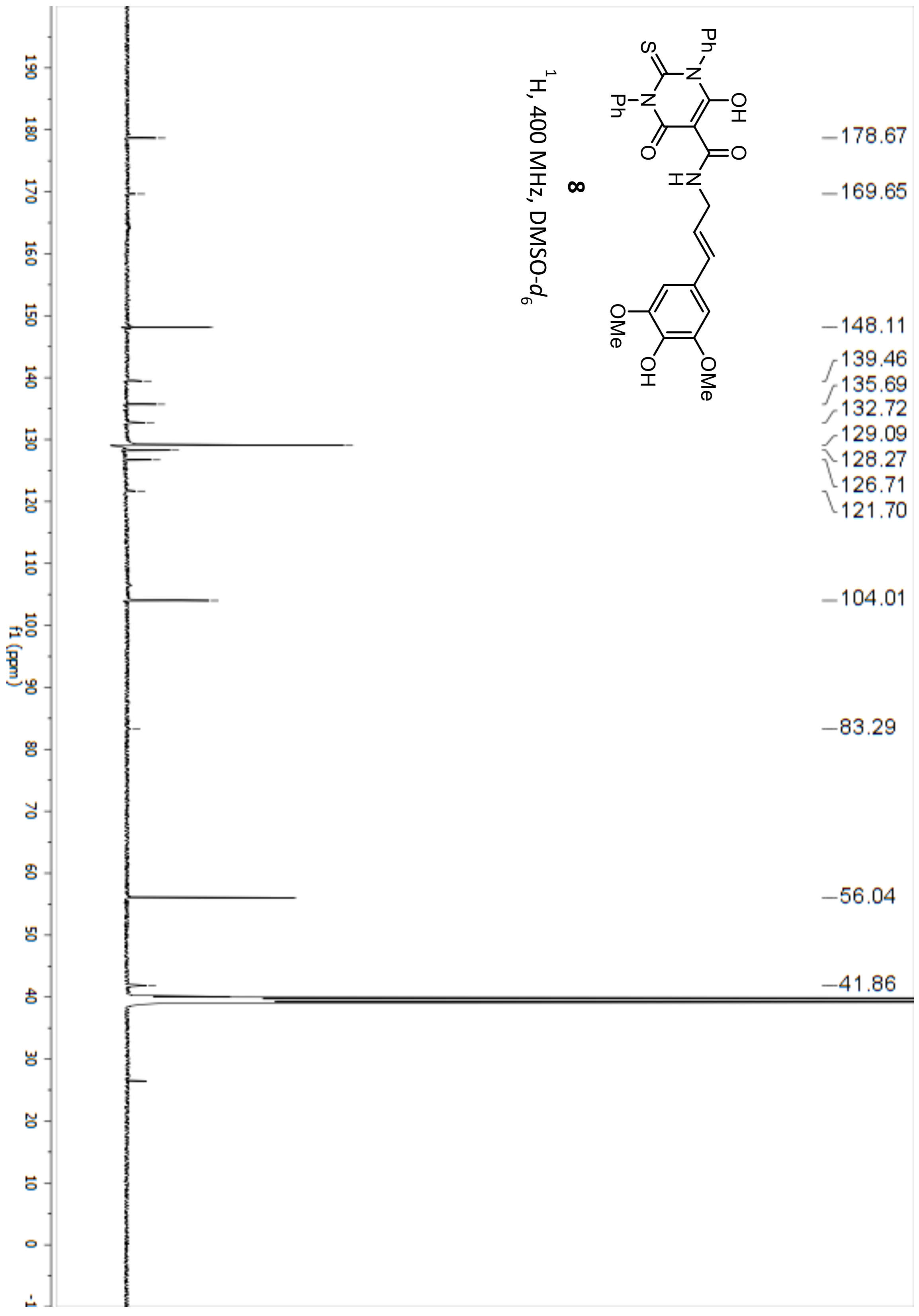

8
1H, 400 MHz, DMSO-d6
178.67
169.65
148.11
139.46
135.69
132.72
129.09
128.27
126.71
121.70
104.01
83.29
56.04
41.86
f1 (ppm)

7. Chromatography analysis of key compounds

The chromatographic analyses were run on an ACQUITY UPLC BEH C18 (50x2.1mmID, particle size 1.7µm) with a VanGuard BEH C18 pre-column (5x2.1mmID, particle size 1.7µm) (LogD>1). The mobile phase was 10mM NH_4OAc in H_2O at pH 5 adjusted with AcOH (A) and 10mM NH_4OAc in CH_3CN-H_2O (95:5) at pH 5 (B). The mobile-phase B proportion increased from 10 % to 90 % in 7 min.

Compound **3a**

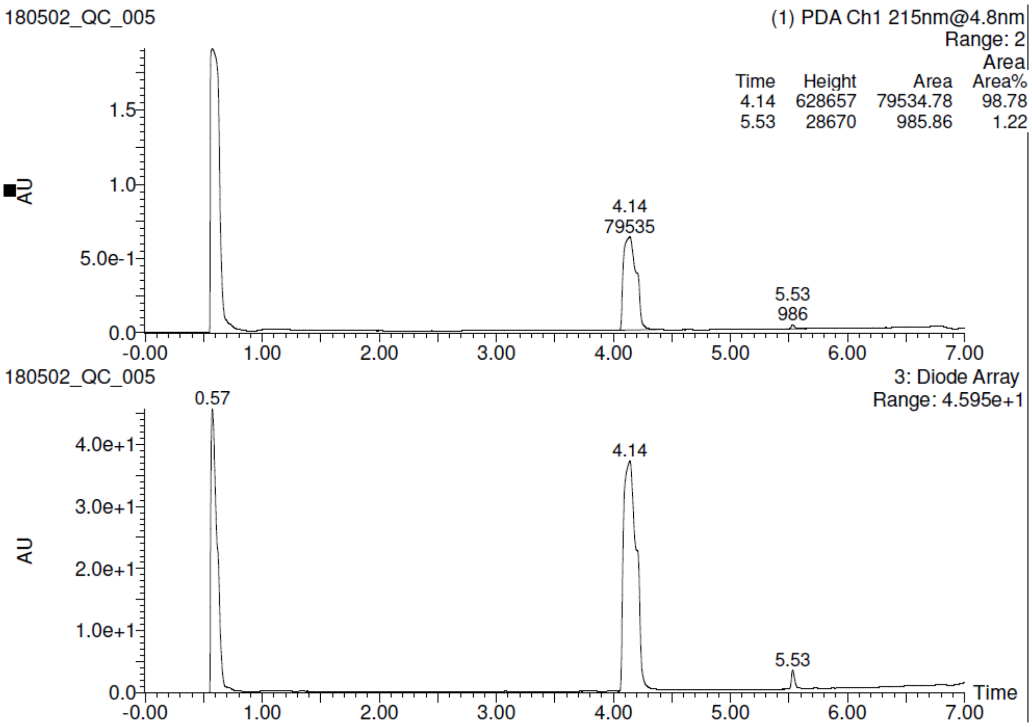

Compound **3b**

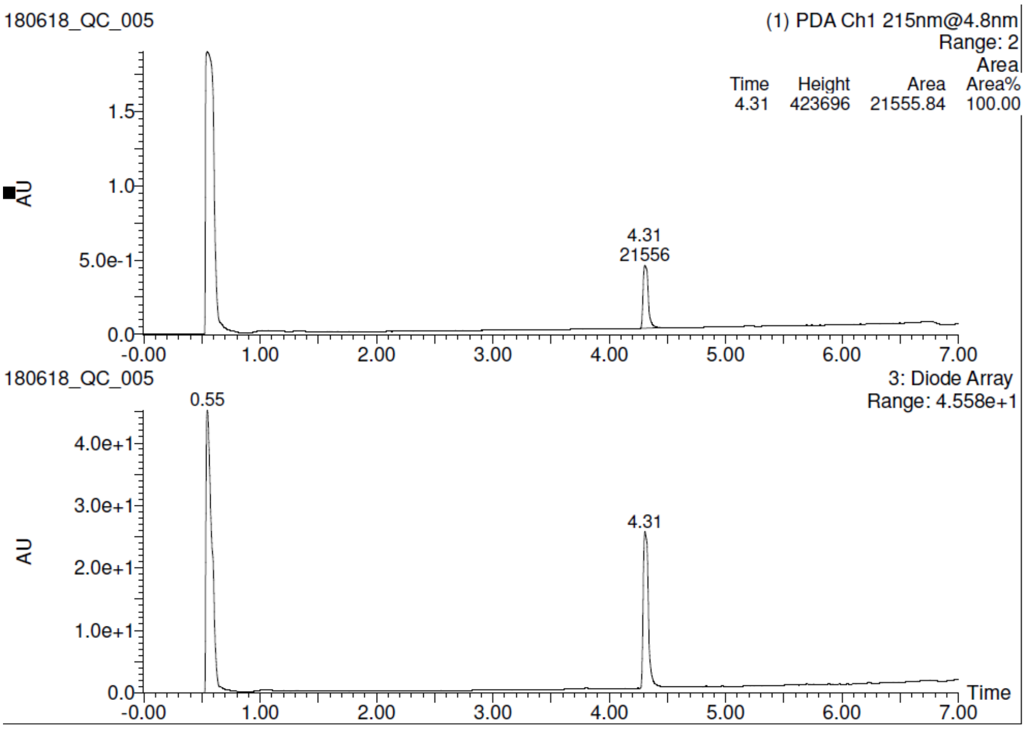

Compound **3c**

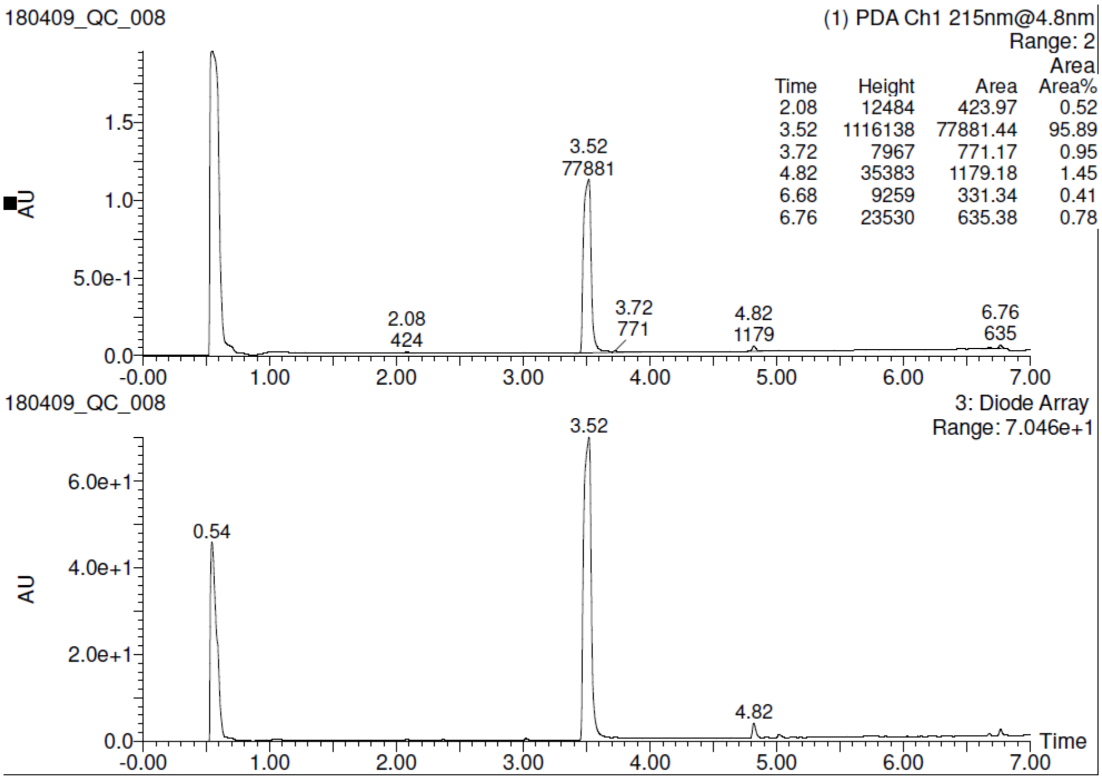

Compound **3e.**

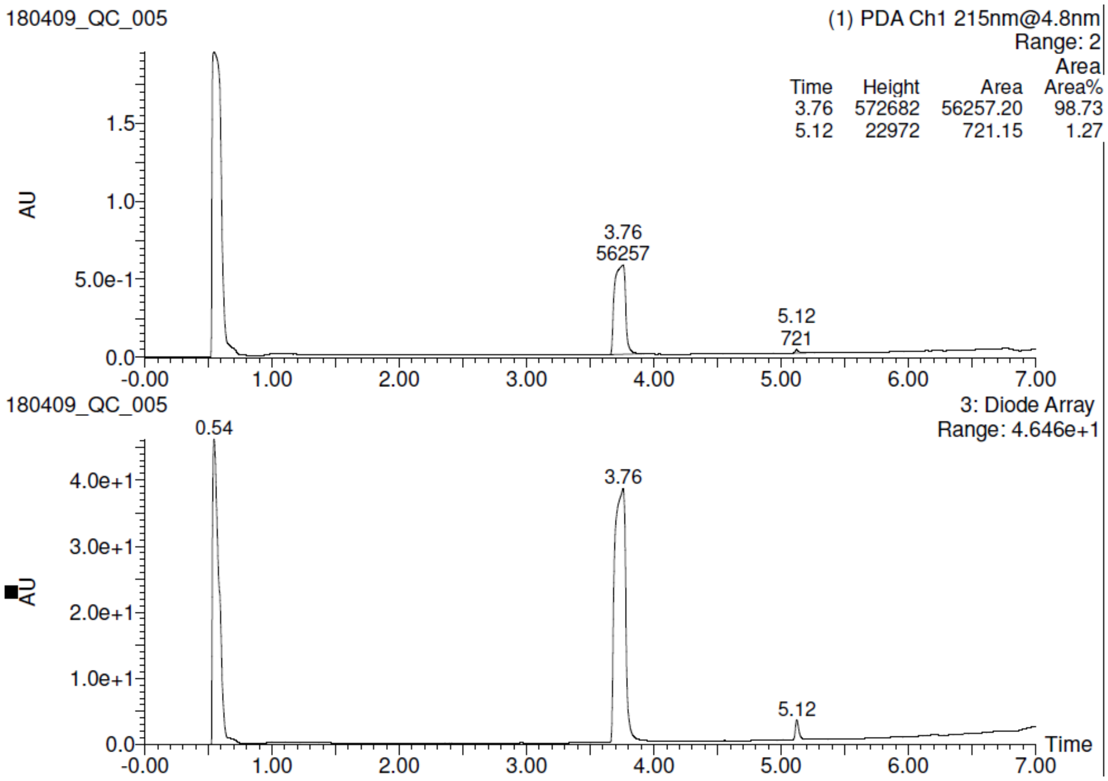

Compound **3h.**

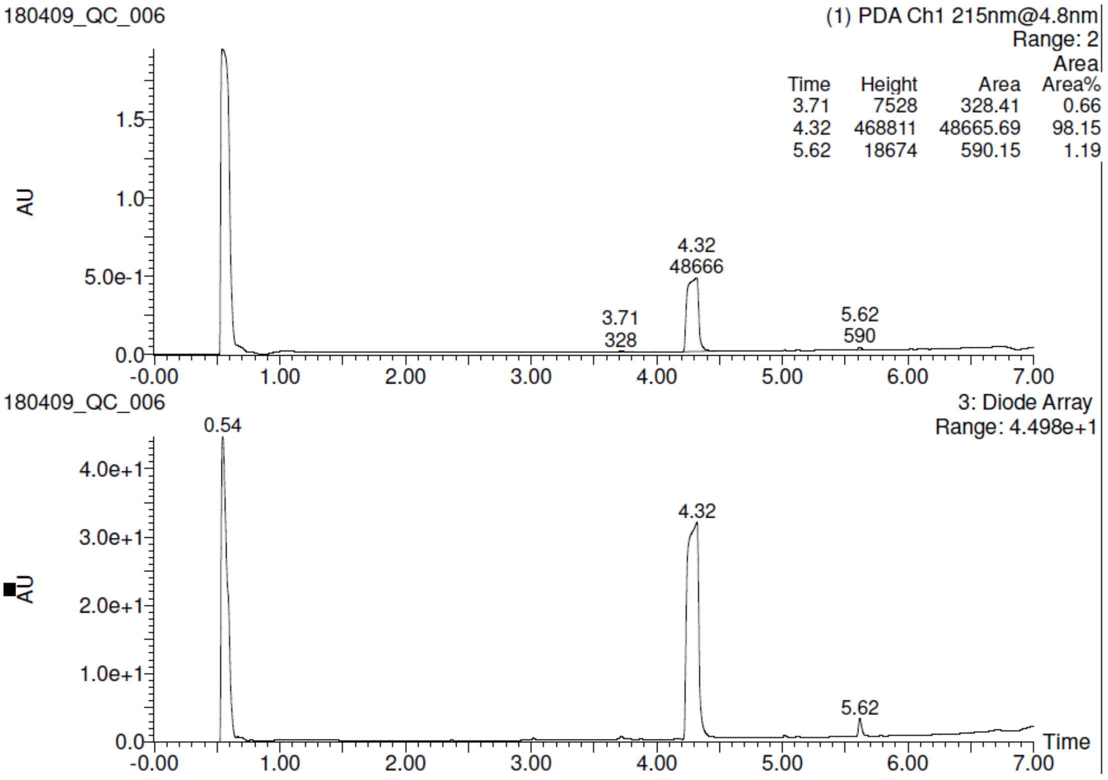

Compound **3g**

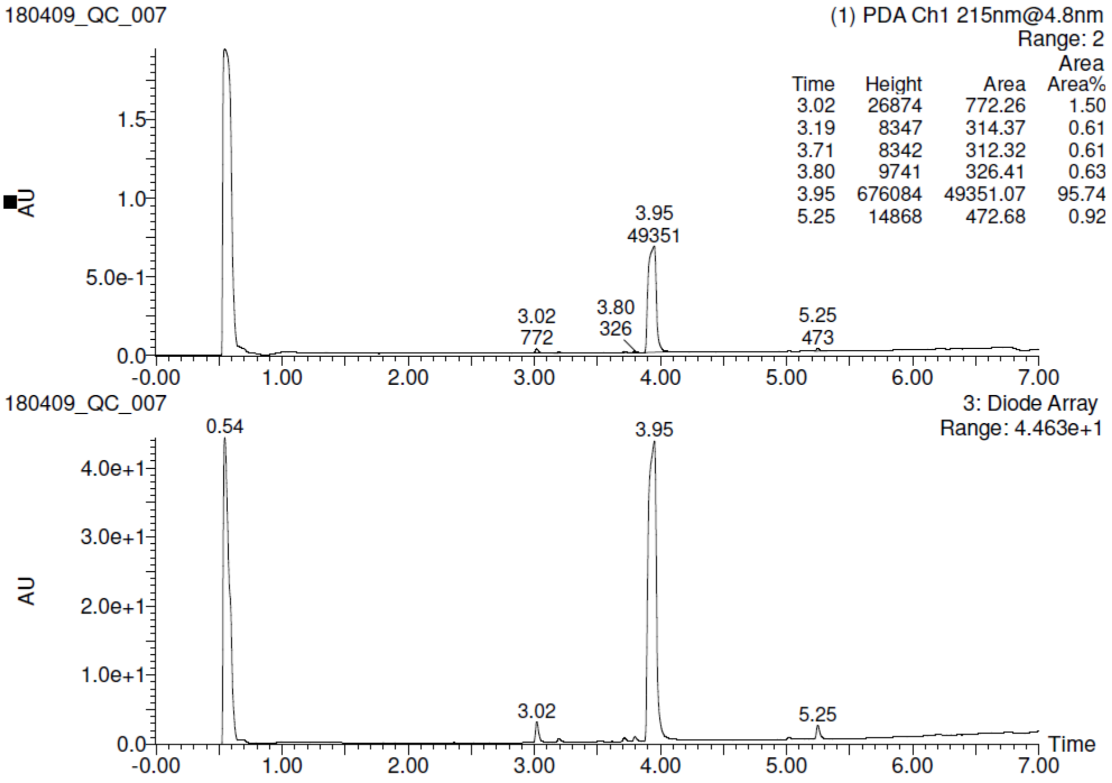

Compound **3h**

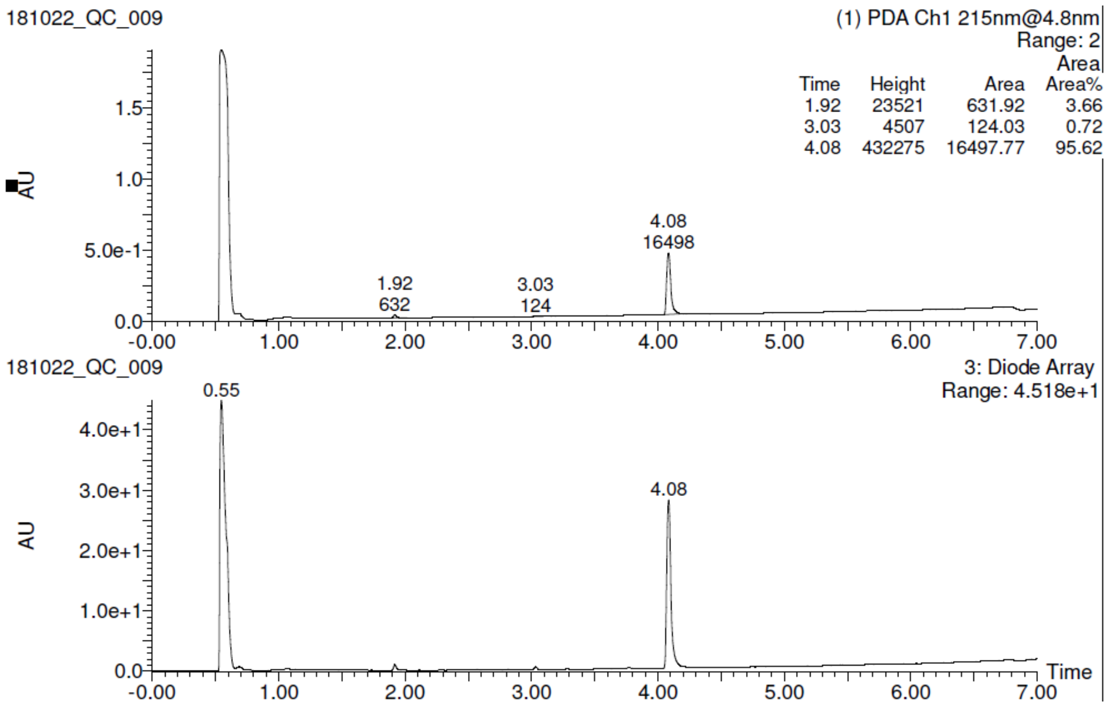

Compound **3i**

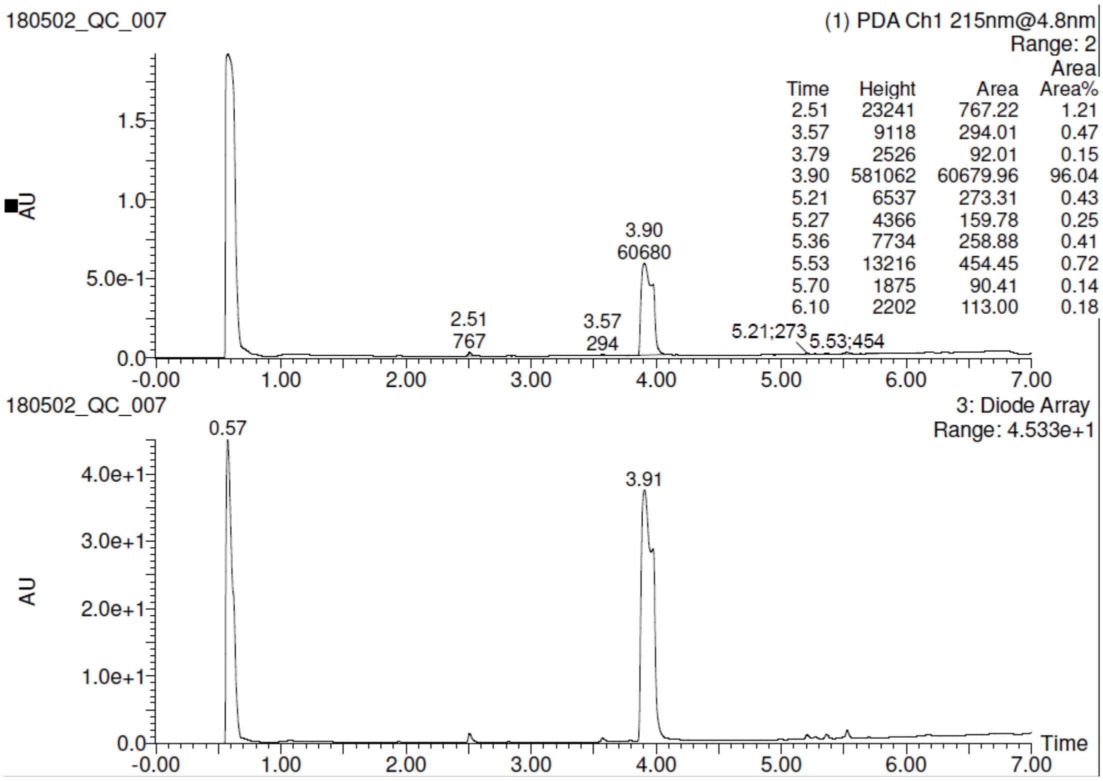

Compound **5d**

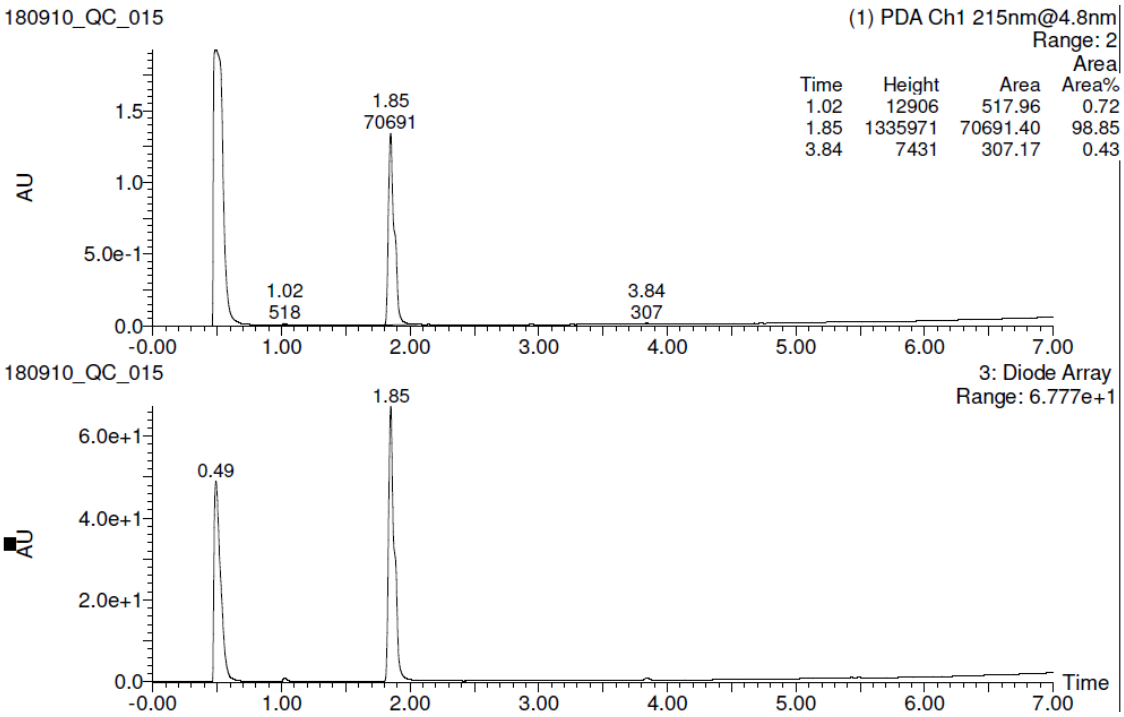

Compound **5e**

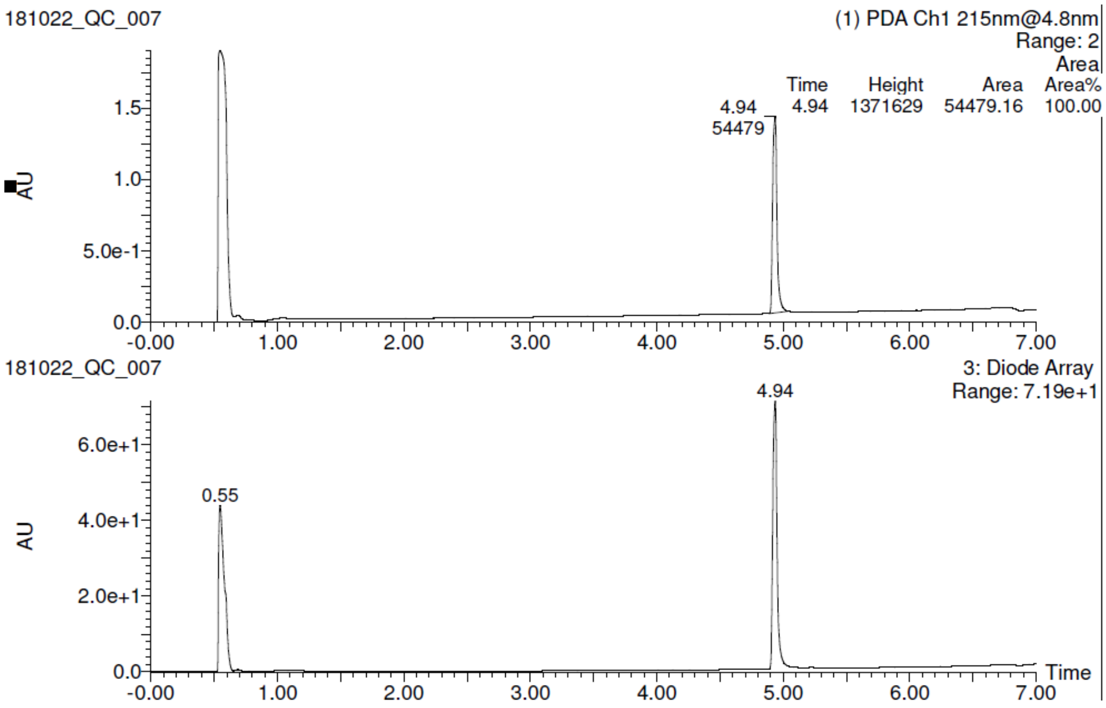

Compound **6**

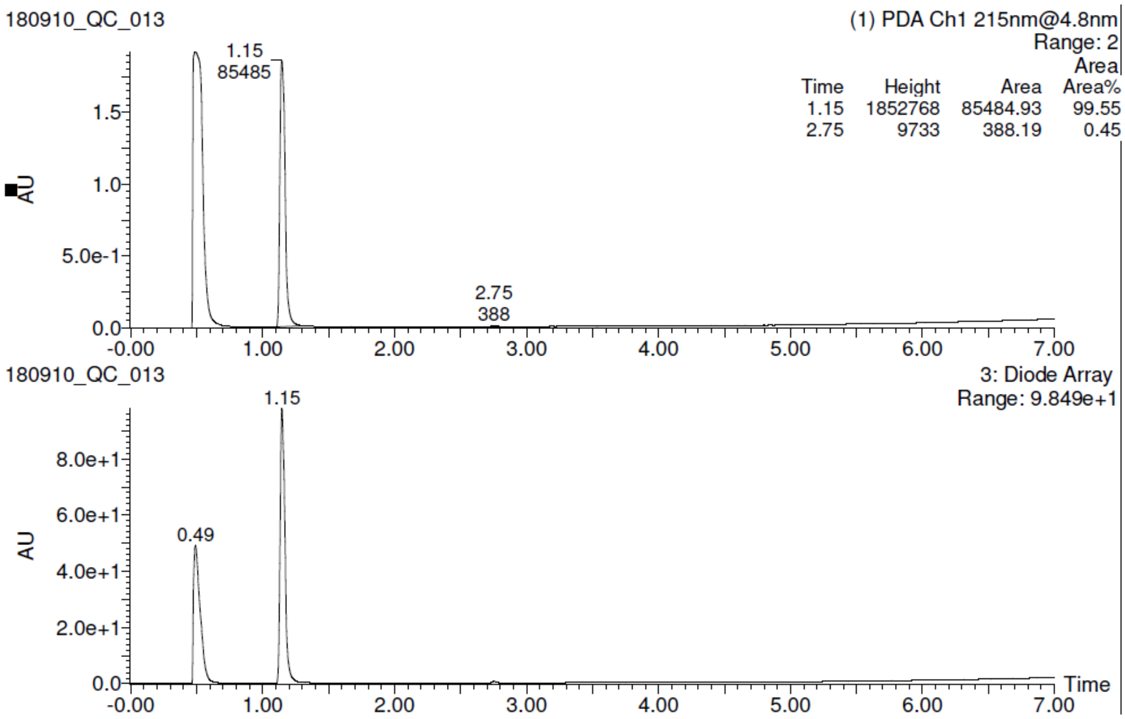

Compound **8**

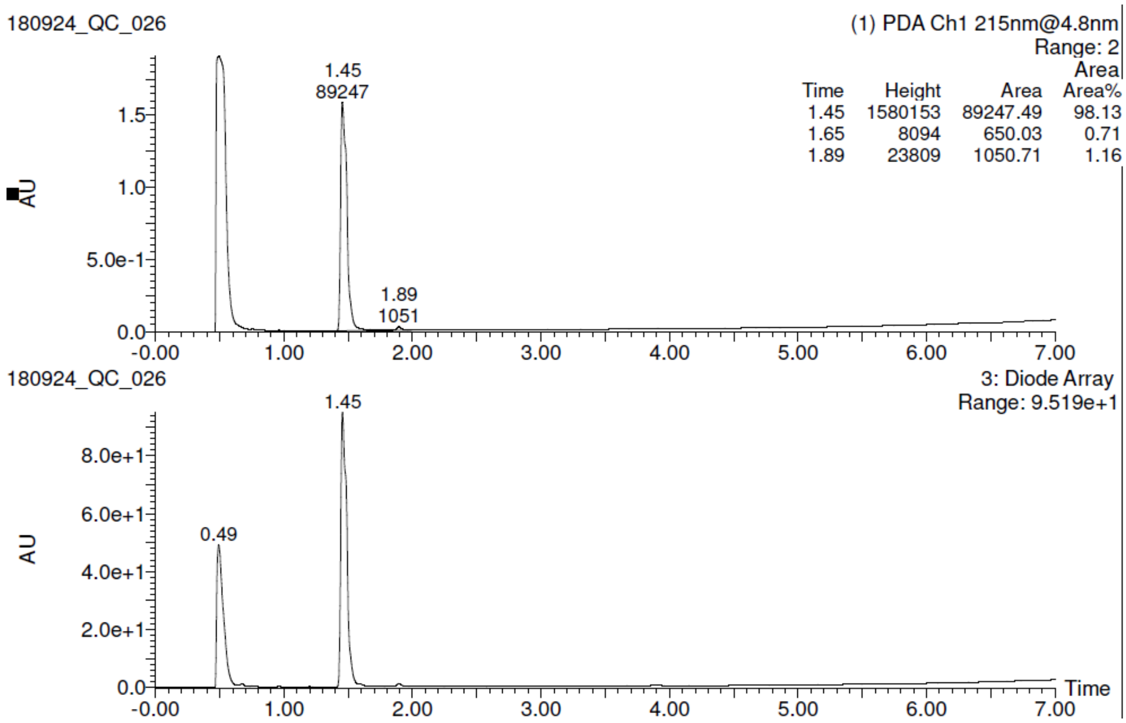

ARRIVE

The ARRIVE Guidelines Checklist

Animal Research: Reporting In Vivo Experiments

Carol Kilkenny[1], William J Browne[2], Innes C Cuthill[3], Michael Emerson[4] and Douglas G Altman[5]

[1]The National Centre for the Replacement, Refinement and Reduction of Animals in Research, London, UK, [2]School of Veterinary Science, University of Bristol, Bristol, UK, [3]School of Biological Sciences, University of Bristol, Bristol, UK, [4]National Heart and Lung Institute, Imperial College London, UK, [5]Centre for Statistics in Medicine, University of Oxford, Oxford, UK.

	ITEM	RECOMMENDATION	Section/ Paragraph
Title	1	Provide as accurate and concise a description of the content of the article as possible.	done
Abstract	2	Provide an accurate summary of the background, research objectives, including details of the species or strain of animal used, key methods, principal findings and conclusions of the study.	done
**INTRODUCTION**			
Background	3	a. Include sufficient scientific background (including relevant references to previous work) to understand the motivation and context for the study, and explain the experimental approach and rationale.<br>b. Explain how and why the animal species and model being used can address the scientific objectives and, where appropriate, the study's relevance to human biology.	done
Objectives	4	Clearly describe the primary and any secondary objectives of the study, or specific hypotheses being tested.	done
**METHODS**			
Ethical statement	5	Indicate the nature of the ethical review permissions, relevant licences (e.g. Animal [Scientific Procedures] Act 1986), and national or institutional guidelines for the care and use of animals, that cover the research.	Supporting Information<br>Paragraph 3
Study design	6	For each experiment, give brief details of the study design including:<br>a. The number of experimental and control groups.<br>b. Any steps taken to minimise the effects of subjective bias when allocating animals to treatment (e.g. randomisation procedure) and when assessing results (e.g. if done, describe who was blinded and when).<br>c. The experimental unit (e.g. a single animal, group or cage of animals).<br>A time-line diagram or flow chart can be useful to illustrate how complex study designs were carried out.	Supporting Information<br>Paragraph 3/4
Experimental procedures	7	For each experiment and each experimental group, including controls, provide precise details of all procedures carried out. For example:<br>a. How (e.g. drug formulation and dose, site and route of administration, anaesthesia and analgesia used [including monitoring], surgical procedure, method of euthanasia). Provide details of any specialist equipment used, including supplier(s).<br>b. When (e.g. time of day).<br>c. Where (e.g. home cage, laboratory, water maze).<br>d. Why (e.g. rationale for choice of specific anaesthetic, route of administration, drug dose used).	Supporting Information<br>Paragraph 3/4

Experimental animals	8	a. Provide details of the animals used, including species, strain, sex, developmental stage (e.g. mean or median age plus age range) and weight (e.g. mean or median weight plus weight range).<br>b. Provide further relevant information such as the source of animals, international strain nomenclature, genetic modification status (e.g. knock-out or transgenic), genotype, health/immune status, drug or test naïve, previous procedures, etc.	Supporting Information<br>Paragraph 3/4

The ARRIVE guidelines. Originally published in *PLoS Biology*, June 2010[1]

Housing and husbandry	9	Provide details of:<br>a. Housing (type of facility e.g. specific pathogen free [SPF]; type of cage or housing; bedding material; number of cage companions; tank shape and material etc. for fish).<br>b. Husbandry conditions (e.g. breeding programme, light/dark cycle, temperature, quality of water etc for fish, type of food, access to food and water, environmental enrichment).<br>c. Welfare-related assessments and interventions that were carried out prior to, during, or after the experiment.	Supporting Information<br>Paragraph 3
Sample size	10	a. Specify the total number of animals used in each experiment, and the number of animals in each experimental group.<br>b. Explain how the number of animals was arrived at. Provide details of any sample size calculation used.<br>c. Indicate the number of independent replications of each experiment, if relevant.	Supporting Information<br>Paragraph 4
Allocating animals to experimental groups	11	a. Give full details of how animals were allocated to experimental groups, including randomisation or matching if done.<br>b. Describe the order in which the animals in the different experimental groups were treated and assessed.	Supporting Information<br>Paragraph 4
Experimental outcomes	12	Clearly define the primary and secondary experimental outcomes assessed (e.g. cell death, molecular markers, behavioural changes).	Supporting Information<br>Paragraph 4
Statistical methods	13	a. Provide details of the statistical methods used for each analysis.<br>b. Specify the unit of analysis for each dataset (e.g. single animal, group of animals, single neuron).<br>c. Describe any methods used to assess whether the data met the assumptions of the statistical approach.	Supporting Information<br>Statistical analysis is not required
RESULTS			
Baseline data	14	For each experimental group, report relevant characteristics and health status of animals (e.g. weight, microbiological status, and drug or test naïve) prior to treatment or testing. (This information can often be tabulated).	done
Numbers analysed	15	a. Report the number of animals in each group included in each analysis. Report absolute numbers (e.g. 10/20, not 50%[2]).<br>b. If any animals or data were not included in the analysis, explain why.	done
Outcomes and estimation	16	Report the results for each analysis carried out, with a measure of precision (e.g. standard error or confidence interval).	done
Adverse events	17	a. Give details of all important adverse events in each experimental group.<br>b. Describe any modifications to the experimental protocols made to reduce adverse events.	No adverse effects.<br>No modifications
DISCUSSION			
Interpretation/ scientific implications	18	a. Interpret the results, taking into account the study objectives and hypotheses, current theory and other relevant studies in the literature.<br>b. Comment on the study limitations including any potential sources of bias, any limitations of the animal model, and the imprecision associated with the results[2].<br>c. Describe any implications of your experimental methods or findings for the replacement, refinement or reduction (the 3Rs) of the use of animals in research.	done
Generalisability/ translation	19	Comment on whether, and how, the findings of this study are likely to translate to other species or systems, including any relevance to human biology.	done

Funding	20	List all funding sources (including grant number) and the role of the funder(s) in the study.	No funding for in vivo studies

References:
1. Kilkenny C, Browne WJ, Cuthill IC, Emerson M, Altman DG (2010) Improving Bioscience Research Reporting: The ARRIVE Guidelines for Reporting Animal Research. *PLoS Biol* 8(6): e1000412. doi:10.1371/journal.pbio.1000412
2. Schulz KF, Altman DG, Moher D, the CONSORT Group (2010) CONSORT 2010 Statement: updated guidelines for reporting parallel group randomised trials. *BMJ* 340:c332.
